# Specificity protein (Sp) transcription factors Sp1, Sp3 and Sp4 are non-oncogene addiction genes in cancer cells

**DOI:** 10.18632/oncotarget.7925

**Published:** 2016-03-05

**Authors:** Erik Hedrick, Yating Cheng, Un-Ho Jin, Kyounghyun Kim, Stephen Safe

**Affiliations:** ^1^ Department of Veterinary Physiology and Pharmacology, Texas A&M University, College Station, TX 77843, USA; ^2^ Environmental Health, University of Cincinnati, Cincinnati, OH 45267, USA

**Keywords:** Sp transcription factors, non-oncogene addiction, cancer

## Abstract

Specificity protein (Sp) transcription factor (TF) Sp1 is overexpressed in multiple tumors and is a negative prognostic factor for patient survival. Sp1 and also Sp3 and Sp4 are highly expressed in cancer cells and in this study, we have used results of RNA interference (RNAi) to show that the three TFs individually play a role in the growth, survival and migration/invasion of breast, kidney, pancreatic, lung and colon cancer cell lines. Moreover, tumor growth in athymic nude mice bearing L3.6pL pancreatic cancer cells as xenografts were significantly decreased in cells depleted for Sp1, Sp3 and Sp4 (combined) or Sp1 alone. Ingenuity Pathway Analysis (IPA) of changes in gene expression in Panc1 pancreatic cancer cells after individual knockdown of Sp1, Sp3 and Sp4 demonstrates that these TFs regulate genes and pathways that correlated with the functional responses observed after knockdown but also some genes and pathways that inversely correlated with the functional responses. However, causal IPA analysis which integrates all pathway-dependent changes in all genes strongly predicted that Sp1-, Sp3- and Sp4-regulated genes were associated with the pro-oncogenic activity. These functional and genomic results coupled with overexpression of Sp transcription factors in tumor vs. non-tumor tissues and decreased Sp1 expression with age indicate that Sp1, Sp3 and Sp4 are non-oncogene addiction (NOA) genes and are attractive drug targets for individual and combined cancer chemotherapies.

## INTRODUCTION

Specificity protein (Sp) transcription factors (TFs) Sp1, Sp3 and Sp4 are members of the Sp/Krüppel-like family (KLF), and results from Sp knockout mouse models demonstrate the importance of Sp genes to embryonic growth and early development [reviewed in 1]. However, expression of Sp1 in humans and rodents decreases with age [2–4]. Moreover, several studies report that high expression of Sp1 and, in some cases, Sp3 in tumor vs. non-tumor tissue are negative prognostic factors for patients with pancreatic, glioma, colon, gastric, head and neck, prostate, lung and breast cancers [5–13]. Studies in cancer cell lines show that Sp1, Sp3 and Sp4 are highly expressed, and RNA interference (RNAi) studies indicate that Sp transcription factors regulate genes associated with cell proliferation, survival and migration/invasion [reviewed in 14]. Although Sp1, Sp3 and Sp4 have similar modular structures and bind GC-rich promoter sequences, these transcription factors also exhibit unique properties including the number of isoforms and DNA binding characteristics [15–17]. Moreover, since Sp1 regulates expression of both pro-oncogenic and tumor suppressor-like genes, it has been suggested that “a more complete understanding of the function of Sp1 in cancer is required to validate its potential as a therapeutic target” [17].

Most studies in cancer cells have focused on Sp1 [17] and there is evidence showing that knockdown of Sp1 by RNA interference (RNAi) in cancer cell lines inhibits cell growth, survival and migration/invasion [18–21]. Although there are a few reports indicating that Sp1, Sp3 and Sp4 differentially regulate some genes and coregulate others [18-20, 22-24], the functional roles of Sp3 and Sp4 compared to Sp1 in cancer cells have not been extensively investigated. In this study, we show that individual knockdown of Sp1, Sp3 and Sp4 by RNAi in SKBR3 and MDA-MB-231 breast, A549 lung, SW480 colon, 786-O kidney, and Panc1, L3.6pL and MiaPaCa2 pancreatic cancer cell lines results in inhibition of cell growth, decreased survival, and inhibition of migration/invasion. Thus, all three Sp transcription factors exhibit pro-oncogenic activity. Using Panc1 cells as a model, a causal Ingenuity Pathway Analysis (IPA) of changes in gene expression after knockdown of Sp1, Sp3 and Sp4 strongly correlated with observed changes in functional responses for the three Sp proteins. Thus, the oncogenic-like activity of Sp1, Sp3 and Sp4 and Sp-regulated genes coupled with their overexpression in tumor vs. non-tumor tissue suggests that Sp1, Sp3 and Sp4 are non-oncogene addiction (NOA) genes that are “attractive drug targets” [25].

## RESULTS

### Knockdown of Sp transcription factors in cancer cell lines: functional effects

**Figure 1:**
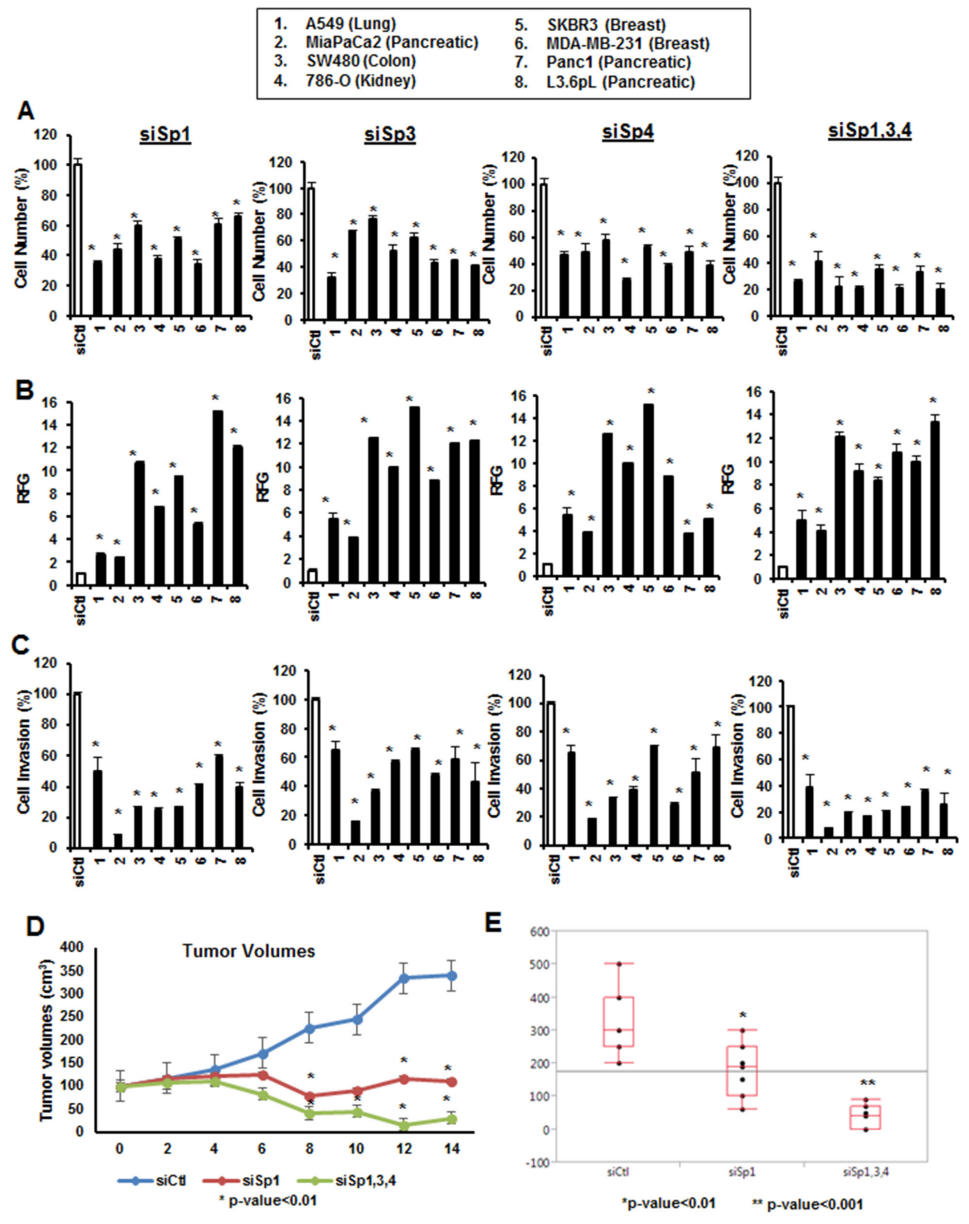
Functional effects of Sp1, Sp3 and Sp4 in A549, MiaPaCa2, SW480, 786-O, SKBR3, MDA-MB-231, Panc1 and L3.6pL cancer cell lines Cells were transfected with siSp1, siSp3 and siSp4 and effects on cell proliferation **A.** Annexin V staining **B.** and invasion in a Boyden chamber assay **C.** were determined as described in the Materials and Methods. Results are expressed as means ± SE for at least 3 biological replicates for each determination, and significant (p<0.05) changes compared to cells transfected with a nonspecific oligonucleotide (siCtl) are indicated (*). Knockdown of Sp1, Sp3 and Sp4 (combined) or Sp1 alone in L3.6pL cells were used in xenografts experiments and changes in tumor volumes **D.** and weights **E.** were determined essentially as described [18–21]. Significant changes (p < 0.05) after Sp knockdown are indicated (*).

The functional and genomic effects of Sp1, Sp3 and Sp4 were investigated by RNAi in several different cancer cell lines. Multiple oligonucleotides for Sp1, Sp3 and Sp4 have previously been used for studying Sp-regulated gene expression and functional responses (Supplementary Figure S1) [18–21], and a single representative oligonucleotide was used for this study. Figure 1 summarizes the effects of knockdown of Sp1, Sp3 and Sp4 in A549, MiaPaCa2, L3.6pL, Panc1, SW480, 786-O, SKBR3 and MDA-MBA-231 cancer cell lines. Decreased individual expression of Sp1, Sp3 and Sp4 in eight different cancer cell lines resulted in significant inhibition of cancer cell proliferation (Figure 1A), induction of Annexin V (a marker of apoptosis) (Figure 1B), and inhibition of cancer cell migration in a Boyden Chamber assay (Figure 1C) and knockdown of all three genes (siSp1, 3, 4) enhanced the observed responses. The magnitude of the effects showed some variability and was dependent on the individual Sp protein and cell context. Most previous functional studies have focused on Sp1; however, results illustrated in Figure 1 clearly demonstrate that both Sp3 and Sp4 also significantly contribute to the growth, survival and migration/invasion of the eight cancer cells lines and comparable effects were observed after individual knockdown of these transcription factors. In a parallel experiment, combined knockdown of Sp1, Sp3 and Sp4 or individual knockdown of Sp1 in L3.6pL cells used in an athymic nude mouse xenograft model showed that loss of Sp TFs resulted in a significant inhibition of tumor growth and tumor weights (Figure 1D and 1E).

### Knockdown of Sp1, Sp3 and Sp4 in cancer cell lines: effects on Sp TFs and Sp-regulated gene products

**Figure 2:**
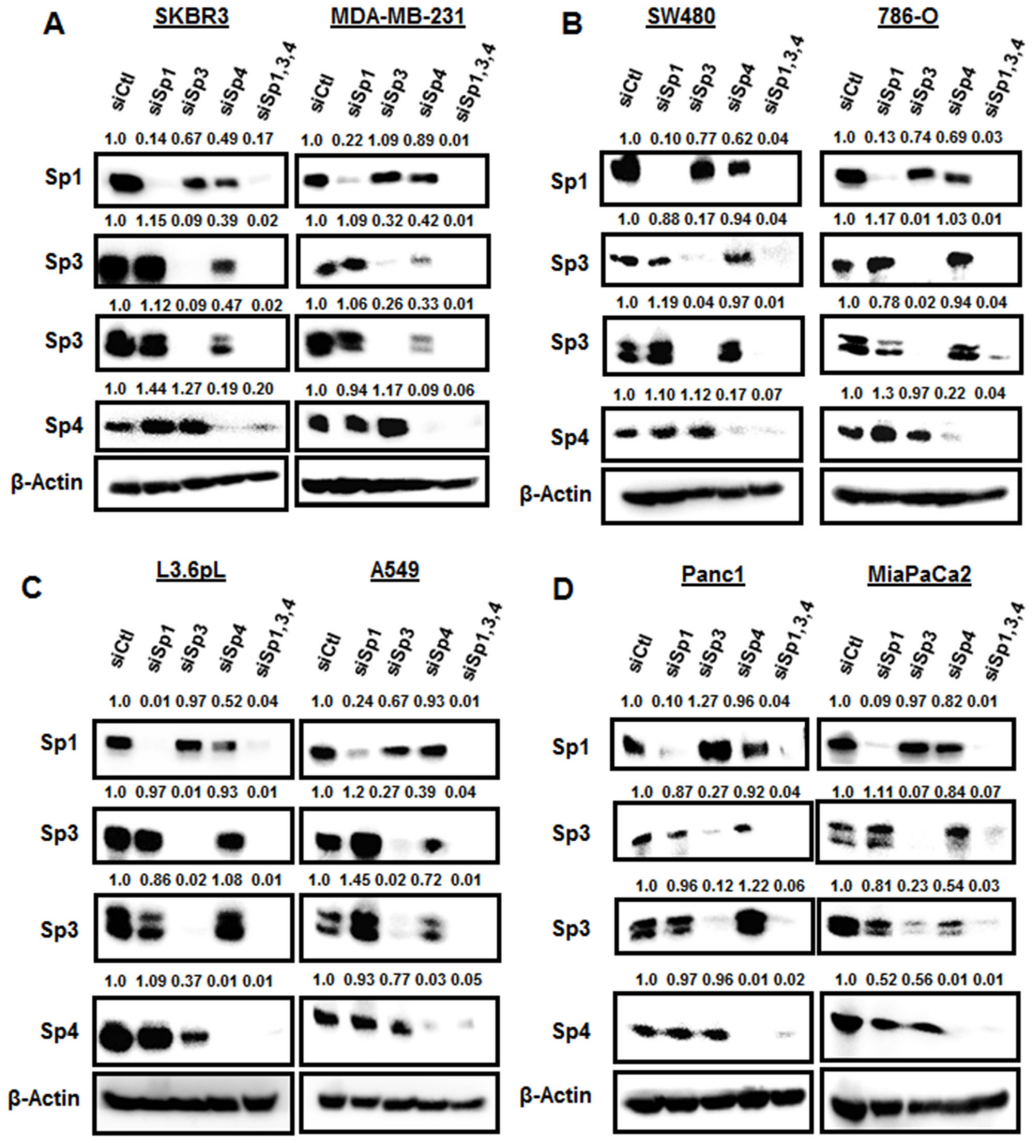
Knockdown of Sp TFs by RNAi **A.** SKBR3 and MDA-MBA-231, **B.** SW480 and 786-O, **C.** L3.6PL and A549, and **D.** Panc1 and MiaPaCa2 cells were transfected with siSp1, siSp3 and siSp4, and whole cell lysates were analyzed by Western blots as outlined in the Materials and Methods.

**Figure 3:**
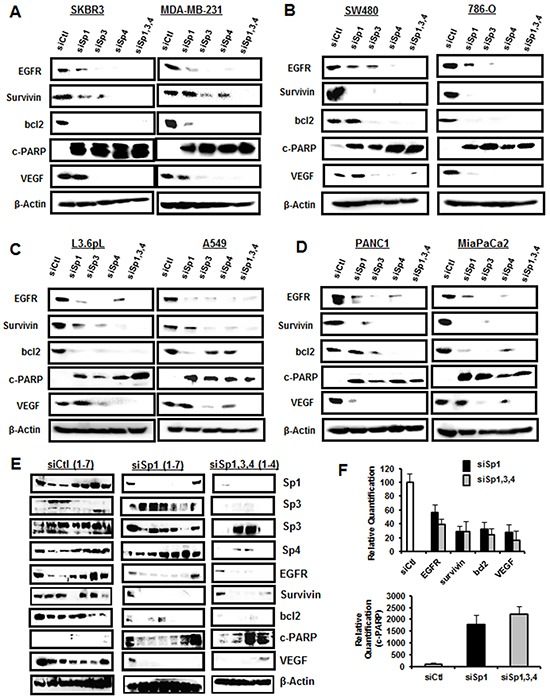
Knockdown of Sp TFs by RNAi decreases expression of Sp-regulated gene products Cell lines **A–B.** were transfected as described in Figure 2 and these same lysates were analyzed for expression of Sp-regulated gene products as outlined in the Materials and Methods. Tumor lysates from mice bearing wild-type or Sp-depleted L3.6pL cells were analyzed by western blots **E.** and band intensities (normalized to β-actin) were quantitated **F.** and are expressed as means ± SE and compared to values from wild-type mice (set at 100%). Significant decreases or increases are indicated (*).

The individual effects of Sp1, Sp3 and Sp4 knockdown could be indirect since the three genes contain GC-rich promoters and they could be self-regulatory [26–28]. Previous studies demonstrated high specificity for knockdown of Sp1, Sp3 and Sp4 by RNAi in RD rhabdomyosarcoma and KU7 bladder cancer cells [20, 24], whereas in other cell lines, knockdown of an individual Sp protein also decreased expression of one or both of the other gene products [18–24]. For example, knockdown of Sp1 (siSp1) or Sp3 (siSp3) in 253JB-V bladder cancer cells decreased expression of Sp4 protein, whereas siSp4 did not affect levels of Sp3 or Sp1 proteins [24]. Western blot analysis of expression of Sp TFs after transfection of the cancer cell lines with siControl (siCtl, non-specific oligonucleotide) or oligonucleotides targeting Sp1 (siSp1), Sp3 (siSp3), Sp4 (siSp4), or their combination (siSp1,3,4) showed that Sp1, Sp3 and Sp4 proteins were highly expressed in the eight cancer cell lines and this includes both the high and low molecular weight bands for Sp3 [15, 29] (Figure 2). Knockdown of Sp1, Sp3 and Sp4 was relatively specific for the individual Sp proteins only in Panc1 cells. In contrast, siSp3 decreased expression of Sp1 (SKBR3, SW480 and A549) and Sp4 (L3.6pL and MiaPaCa2) proteins; siSp4 decreased Sp1 (SKBR3, SW480, 786-O and L3.6pL) and Sp3 (SKBR3, MiaPaCa2 and MDA-MB-231) proteins; and siSp1 decreased Sp3 (786-O) and Sp4 (MiaPaCa2) proteins. These results are quantitated in Supplementary Figure S2 and demonstrate that autoregulation of Sp1, Sp3 and Sp4 was observed in seven of the eight cancer cell lines and primarily involved Sp3 and Sp4 and their regulation of each other or of Sp1. Sp TFs regulate expression of several pro-oncogenic factors including vascular endothelial growth factor (VEGF), epidermal growth factor receptor (EGFR), survivin and bcl2 [14]. In this study, we examined the effects of Sp knockdown on expression of these genes and induction of the apoptotic marker cleaved PARP in the cancer cell lines. Transfection of siSp1, siSp3, siSp4 and siSp1,3,4 induced PARP cleavage in all eight cell lines (Figure 3) and this complemented the increased Annexin V staining (Figure 1B) observed after the same treatments. EGFR, VEGF, bcl-2 and survivin were expressed in the eight cancer cell lines, and siSp1,3,4 downregulated these gene products; however, the effects of siSp1, siSp3 and siSp4 were variable and there were also gene- and cell context-specific differences. For example, expression of EGFR was decreased in the eight cancer cell lines after transfection of siSp1, siSp3 and siSp4 and expression of all of gene products (EGFR, survivin, bcl-2 and VEGF) were decreased in cells transfected with siSp3 and siSp4 but variable responses were observed for siSp1 (Figures 3A–3D). Western blot analysis of tumor lysates (derived from L3.6pL cells as xenografts) also showed that the loss of Sp TFs resulted in decreased expression of Sp-regulated gene products, survivin, bcl-2 and EGFR and also decreased expression of Sp TFs (Figure 3E). The knockdown of Sp TFs persisted throughout the experiment except for the increased expression of the lower molecular weight Sp3 band in tumors from 2/4 mice in which L3.6pL cells were transfected with siSp1,3,4. These results further demonstrate the effective silencing of Sp transcription factors by RNAi using oligonucleotides over the relatively short (14 day) duration of the xenograft study due to the rapid growth of L3.6pL-derived tumors [30, 31]. Thus, the *in vivo* results complemented *in vitro* studies and confirmed the pro-oncogenic functions of Sp TFs.

### Analysis of gene expression changes in Panc1 cells after knockdown of Sp1, Sp3 and Sp4

**Figure 4:**
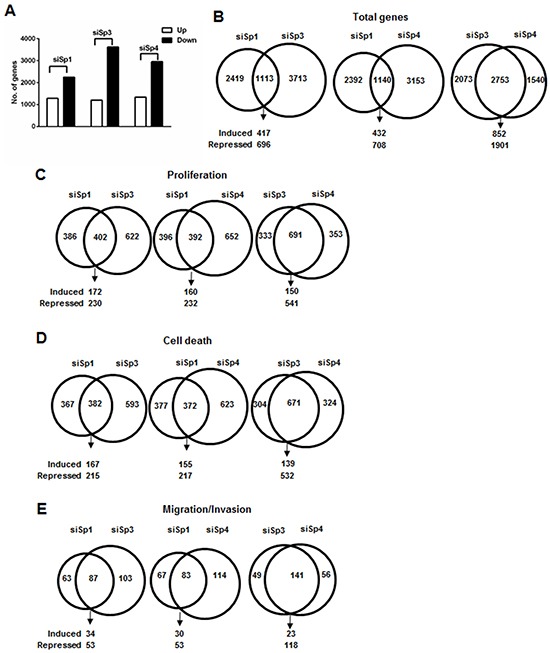
Analysis of changes in gene expression after knockdown of Sp1, Sp3 and Sp4 in Panc1 cells **A.** Panc1 cells were transfected with siSp1, siSp3 or siSp4, and changes in gene expression were determined using Human HT-12 V4 expression bead chip arrays. The overlap of total genes **B.** and growth inhibition **C.** cell death **D.** and inhibition of migration/invasion **E.** genes coregulated by Sp1/Sp3, Sp1, Sp4 and Sp3/Sp4 in Panc1 cells after RNAi was determined by IPA.

**Figure 5:**
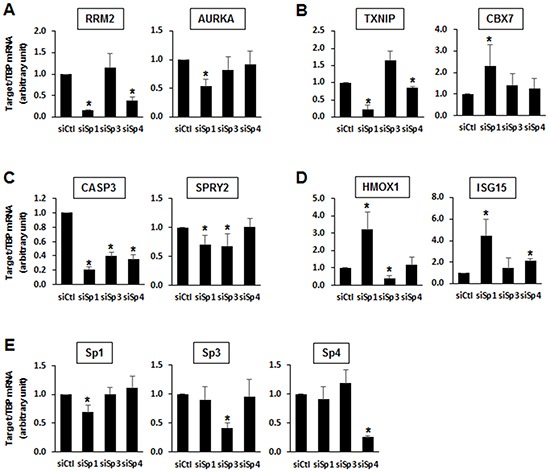
Changes in expression of specific genes after Sp knockdown in Panc1 cells Panc1 cells were transfected with siSp1, siSp3 or siSp4, and real time PCR analysis was used to determine changes in expression of **A.** RRM2 and AURKA, **B.** TXNIP and CBX7, **C.** CASP3 and SPRY2, **D.** HMOX1 and ISG15, and **E.** Sp1, Sp3 and Sp4. Results are expressed as means ± SE for at least 3 replicates for each treatment group, and significantly (p<0.05) decreased changes in gene expression are indicated (*).

Results of RNAi studies show that Sp1, Sp3 and Sp4 exhibited pro-oncogenic activity and regulated pro-oncogenic factors (Figures 1 and 3), and this was further investigated in gene array studies using Panc1 cells as a model. Transfection of Panc1 cells with siSp1, siSp3 and siSp4 and analysis of gene expression using arrays resulted in inhibition or induction of 3,532, 4,826 and 4,293 genes, respectively (Figure 4A). After knockdown of Sp1, Sp3 and Sp4, Venn diagrams show considerable overlap of genes commonly regulated by Sp1:Sp3 (1,113); Sp1:Sp4 (1,114) and Sp3:Sp4 (2,753) with the most pronounced gene overlap observed for Sp3 and Sp4 (Figure 4B). IPA was used to investigate common and differentially expressed genes after knockdown of Sp1, Sp3 and Sp4 associated with cell proliferation, survival and migration/invasion and there were significant changes in total gene expression associated with cell proliferation (788, 1,204 and 1,044 genes, respectively), survival (759, 975 and 995 genes, respectively) and migration/invasion (150, 190 and 197 genes, respectively) (Figure 4C–4E). Venn diagrams also showed that there was a considerable overlap of common genes coregulated by Sp1:Sp3, Sp1:Sp4 and Sp3:Sp4 associated with cell proliferation (Figure 4C), survival (Figure 4D) and migration/invasion (Figure 4E). For example, after knockdown of Sp3 and Sp4 by RNAi, there was a 60-70% overlap of genes associated with Panc1 cell proliferation, survival and migration/invasion and this correlated with their common regulation of total genes (Figure 4B). Examination of the changes in gene expression after RNAi showed that there were Sp1-, Sp3- and Sp4-regulated genes that both correlated or inversely correlated with the observed functional responses induced by knockdown of Sp TFs (Figure 1). This was confirmed by real time PCR analysis (Figure 5) showing that one or more Sp TFs decreased expression of the tumor promoting genes ribonucleotide reductase M2 (RRM2) and Aurora kinase A (AURKA) (Figure 5A) and increases expression of the tumor suppressor-like genes such as thioredoxin-interacting protein (TXNIP) and the polycomb CBX7 genes (Figure 5B) [32–35]. However, knockdown of one or more Sp TFs also decreased expression of caspase 3 (CASP3) and Sprouty2 (SPRY2) that inhibit pancreatic tumorigenesis (Figure 5C) and increased expression of genes such as heme oxygenase 1 (HMOX1) and interferon-stimulated gene 15 (ISG15) that promote carcinogenesis (Figure 5D) [36–39]. These results are consistent with the IPA of array data showing that Sp TFs regulate genes that both correlate and inversely correlate with the results of functional studies (Supplementary Tables S1–S3).

Many transcription factors also regulate genes that correlate or inversely correlate with their functional responses and therefore we used causal IPA which is a quantitative approach that integrates all of the changes in expression of genes and pathways in large data sets to predict biologic function [40]. Table 1 summarizes the analysis of the total changes in gene expression after knockdown of Sp1, Sp3 and Sp4 in Panc1 cells. The low p-values and activation score values (>2 or <-2, respectively) obtained from this analysis strongly predicted that Sp1, Sp3 and Sp4 were associated with Panc1 cell proliferation, survival and migration/invasion and were consistent with the functional results illustrated in Figure 1. These functional and quantitative genomic data coupled with the high expression of Sp transcription factors in tumor vs. non-tumor tissue suggests that Sp1, Sp3 and Sp4 are NOA genes and attractive drug targets.

**Table 1:** Causal IPA analysis gene functions of Sp knockdown by RNAi in Panc1 cells

siSp	Categories	Diseases or Functions Annotation	p-Value	Predicted Activation State	Activation z-Score	# of Molecules
siSp1	Cell death and survival	Cell death	1.11E-43	Increased	2.821	749
	Cellular growth and proliferation	Proliferation of cells	2.66E-39	Decreased	−3.240	788
	Cellular movement	Migration of tumor cell lines	6.40E-11	Decreased	−2.063	150
siSp3	Cell death and survival	Cell death	1.19E-32	Increased	2.526	975
	Cellular growth and proliferation	Proliferation of cells	8.07E-27	Decreased	−5.410	1024
	Cellular movement	Migration of tumor cell lines	2.54E-08	Decreased	−6.346	190
siSp4	Cell death and survival	Cell death	2.62E-34	Increased	3.809	995
	Cellular growth and proliferation	Proliferation of cells	4.41E-28	Decreased	−6.222	1044
	Cellular movement	Migration of tumor cell lines	2.42E-09	Decreased	−6.411	197

## DISCUSSION

The concept of NOA highlights the fact that the cancer genotype and hallmarks of cancer are maintained by both oncogenes and NOA genes which are also important targets for mechanism-based anticancer agonists [25, 41]. Among Sp/KLF transcription factors, Sp1 has been most extensively investigated and fulfills many of the criteria for an NOA gene. Sp1 levels decrease with age in rodents and humans [2–4] and several studies show that Sp1 levels are high in tumor vs. non-tumor tissue [5–13]. The differential expression of Sp1 has also been observed in human fibroblasts where carcinogen- or oncogene-induced transformation resulted in an 8- to 18-fold increase in Sp1 levels [42]. Moreover, in xenograft experiments, the loss of Sp1 in fibrosarcoma cells decreased their ability to form tumors [42] and the role of Sp1 in tumor growth, survival and migration/invasion has been confirmed in other reports [14, 18–21]. Our results clearly demonstrate for the first time that not only Sp1 but also Sp3 and Sp4 play a role in cancer cell growth, survival and migration/invasion of multiple cancer cell lines (Figure 1) and regulate expression of gene products (Figure 3) consistent with these observations. We also observed that tumor growth in mice bearing L3.6pL pancreatic cancer cells depleted of Sp1 or Sp1, Sp3 and Sp4 (combined) was significantly lower than observed in studies using wild-type cells expressing these TFs (Figure 3E). Moreover, transfection of Panc1 cells with siSp1, siSp3 or siSp4 and IPA of changes in gene expression by arrays showed that all three transcription factors regulated genes that enhance cell proliferation, survival and migration/invasion (Supplementary Tables S1–S3). Despite overlap in their regulation of common genes (Figure 4), RNAi studies on functional effects of Sp TFs (Figure 1) showed that individual loss of Sp1, Sp3 or Sp4 was sufficient to decrease growth, survival and invasion and compensation by the other two Sp genes was not observed, suggesting unique gene regulatory functions for each of these transcription factors and this is currently being investigated.

Sp1 regulates expression of genes that both enhance and inhibit carcinogenesis as indicated in the analysis of our array data (Supplementary Tables S1–S3) and results, illustrated in Figure 5 and this has been raised as a possible cautionary consideration for clinical development of anticancer drugs that specifically target Sp proteins [17]. However, causal IPA approaches which weigh contributions of individual genes to various networks/pathways (Table 1) showed that after knockdown of Sp1, Sp3 or Sp4, changes in expression of all genes/pathways associated with cancer cell proliferation, survival and migration/invasion strongly correlated with the observed functional responses (Figure 1).

Previous studies have reported differences in the prognostic value of Sp1 overexpression in breast and lung cancer patients and also differences in the pro- and anti-carcinogenic role of Sp1 in MDA-MB-231 breast and A549 lung cancer cell lines [10, 43–47]. Using an RNAi approach (Figure 1), our results show that not only Sp1 but also Sp3 and Sp4 exhibit pro-oncogenic activities in MDA-MB-231 and A549 cells. Some of the differences between studies may be due to the methods used to modulate Sp expression since overexpression of Sp1 and Sp3 in some cancer cell lines induces apoptosis and inhibits growth [48–51]. It is possible that overexpressing Sp1, Sp3 and Sp4 may not always be ideal for probing the “constitutive” functions of these transcription factors since high intracellular levels of Sp1, Sp3 and Sp4 resulting from overexpression may activate genes with GC-rich promoters that are not normally expressed, and this is currently being investigated.

In summary, this study indicates that Sp1, Sp3 and Sp4 are NOA genes that are highly expressed in tumor vs. non-tumor tissue and regulate expression of pro-oncogenic factors that contribute to cancer cell growth, survival and migration/invasion. Although many transcription factors are difficult to target, several different classes of antineoplastic agents downregulate Sp transcription factors and these include natural products and their derivatives, metformin, non-steroidal anti-inflammatory drugs, and ROS-inducing anticancer agents, including isothiocyanates, piperlongumine and arsenic trioxide [14, 18–21]. Moreover, drugs, such as ascorbate, tolfenamic acid and betulinic acid that downregulate Sp proteins, are highly effective in drug combinations for inhibiting tumor growth in laboratory animal studies [52–54]. The choice of a specific drug for targeting Sp TFs will be tumor-specific and dependent on pharmacokinetics and efficient delivery of the specific agent to the tumor site. Important advantages for development and clinical applications of anticancer agents that target Sp1, Sp3 and Sp4 include: (a) Sp protein expression in non-tumor tissue is relatively low; (b) in cancer cells, these compounds decrease Sp-regulated genes such as EGFR, VEGF, cMET and other tyrosine kinases that are themselves individual drug targets; and (c) these agents also decrease expression of drug resistance genes (survivin, MDR1) [14] and are ideal for drug combination therapies. Since Sp3 and Sp4 exhibit pro-oncogenic activities and are highly expressed in cancer cells, we are currently investigating the prognostic significance of Sp3 and Sp4 and comparing the results with previous studies on Sp1 to determine which Sp transcription factors (individual or combined) are the most accurate for patient prognosis.

## MATERIALS AND METHODS

### Cell lines and antibodies

Breast (SKBR3, MDA-MB-231), kidney (786-O), colorectal cancer (SW480), lung (A549), and pancreatic (Panc1, L3.6pL, MiaPaCa2) cancer cell lines were purchased from American Type Culture Collection (Manassas, VA). Cells were maintained 37°C in the presence of 5% CO_2_ in Dulbecco's modified Eagle's medium/Ham's F-12 medium with 10% fetal bovine serum with antibiotic or RPMI-1640 Medium with 10% fetal bovine serum and antibiotic. b-Actin antibody, Dulbecco's Modified Eagle's Medium, and RPMI-1640 Medium, and 36% formaldehyde were purchased from Sigma-Aldrich (St. Louis, MO). Hematoxylin was purchased from Vector Laboratories (Burlingame, CA). Sp1 antibody from Millipore (Temecula, CA); Sp3, Sp4, EGFR, bcl2 antibodies from Santa Cruz Biotech (Santa Cruz, CA); survivin antibody from Cell Signaling Technologies (Danvers, MA); VEGF antibody from GeneTex (Irvine, CA). Apoptotic, Necrotic, and Healthy Cells Quantification Kit was purchased from Biotium (Hayward, CA). Cells were visualized as described previously [21].

### Cell proliferation assay and Annexin V staining

Cell proliferation assays were carried out as described previously [18–21], and changes in cell number were determined by Coulter Z1 cell counter. Annexin V staining used the Vybrant apoptosis kit according to the manufacturer's protocol [21].

### Boyden chamber assay

SKBR3, MDA-MB-231, 786-O, SW480, A549, Panc1, L3.6pL, and MiaPaCa2 cancer cells (3.0 × 10^5^ per well) were seeded in Dulbecco's modified Eagle's medium/Ham's F-12 medium supplemented with 2.5% charcoal-stripped fetal bovine serum and were allowed to attach for 24 hr. Cells were seeded and subsequently treated with varying concentrations of panobinostat or vorinostat for 24 hr (± GSH 3 hr prior to treatment) or with 100 nm of siSp1, siSp3, siSp4 for 48 hr. Cells were trypsinized, counted and then placed in 12-well 8.0 mm pore ThinCerts from Greiner Bio-one (Monroe, NC), allowed to migrate for 24 hr, fixed with formaldehyde, and then stained with hematoxylin. Cells that migrated through the pores were then counted as described [21].

### Western blot analysis

SKBR3, MDA-MB-231, 786-O, SW480, A549, Panc1, L3.6pL, and MiaPaCa2 cancer cells (3.0 × 10^5^ per well) were seeded in Dulbecco's modified Eagle's medium/Ham's F-12 medium supplemented with 2.5% charcoal-stripped fetal bovine serum and were allowed to attach for 24 hr. Cells were transfected with 100 nm of siSp1, siSp3 or siSp4 for 72 hr. Cells were analyzed by western blot as described previously [18–21].

### Small interfering RNA interference assay

siRNA experiments were conducted as described previously [18, 19]. The siRNA complexes used in the study are as follows.

siGL2-5′: CGU ACG CGG AAU ACU UCG A
siSp1: SASI_Hs02_00333289 [1] SASI_Hs01_ 00140198 [2] SASI_Hs01_00070995 [3]
siSp3: SASI_Hs01_00211941 [1] SASI_Hs01_ 00211942 [2] SASI_Hs01_00211943 [3]
siSp4: SASI_Hs01_00114420 [1] SASI_Hs01_ 00114421 [2] SASI_Hs01_00114420 [3]

Supplemental Figure 1 shows the comparative effects of Sp knockdown by the various oligonucleotides in L3.6pL cells; with few exceptions, comparable knockdown was observed for the different oligonucleotides against Sp1, Sp3, Sp4 and the combinations of Sp1, Sp3 and Sp4.

### Xenograft studies

Female athymic nude mice (4-6 weeks old) were purchased as previously described [21]. L3.6pL cells in culture were transfected with 100 nM of siCtl (7 mice), siSp1 (7 mice), or siSp1, 3, 4 (7 mice). After 48 hr, 1.0×10^6^ cells were suspended in Matrigel (1:1 ratio) and injected into the right flank of athymic nude mice. Tumor volumes, tumor weights, and tumor lysates were determined and analyzed as previously described [21]. L3.6pL mice rapidly develop tumors in a xenograft model (10-14 days) and within this time period, we observed efficient and persistent decreased expression of Sp1, Sp3 and Sp4 proteins using oligonucleotides compared to shRNAs.

### Microarray and IPA analysis

After knockdown by RNAi total RNA was extracted using a mirVanaTM miRNA Isolation Labeling Kit (Ambion, Austin, TX) and used for microarray analysis with a HumanHT-12 v4 expression beadchip (Illumina, San Diego, CA) according to the manufactures' protocol. Microarray data were normalized and results from replicate (3X) experiments were used to identify differentially expressed genes with a ≥ 1.5-fold change. Function and pathways analysis of Sp-regulated genes was determined using Ingenuity Pathways Analysis (IPA) database (Invitrogen, Carlsbad, CA).

### Statistical analysis

Statistical significance of differences between the treatment groups was determined as previously described [21].

## SUPPLEMENTARY FIGURES AND TABLES








