# Supplementary material for: Specificity protein (Sp) transcription factors Sp1, Sp3 and Sp4 are non-oncogene addiction genes in cancer cells
Source: Oncotarget. 2016 Mar 5;7(16):22245–56. doi: 10.18632/oncotarget.7925 (PMC5008359; doi:10.18632/oncotarget.7925)
Supplement: Supplementary file 1 [file oncotarget-07-22245-s001.pdf]

SUPPLEMENTARY FIGURES AND TABLES

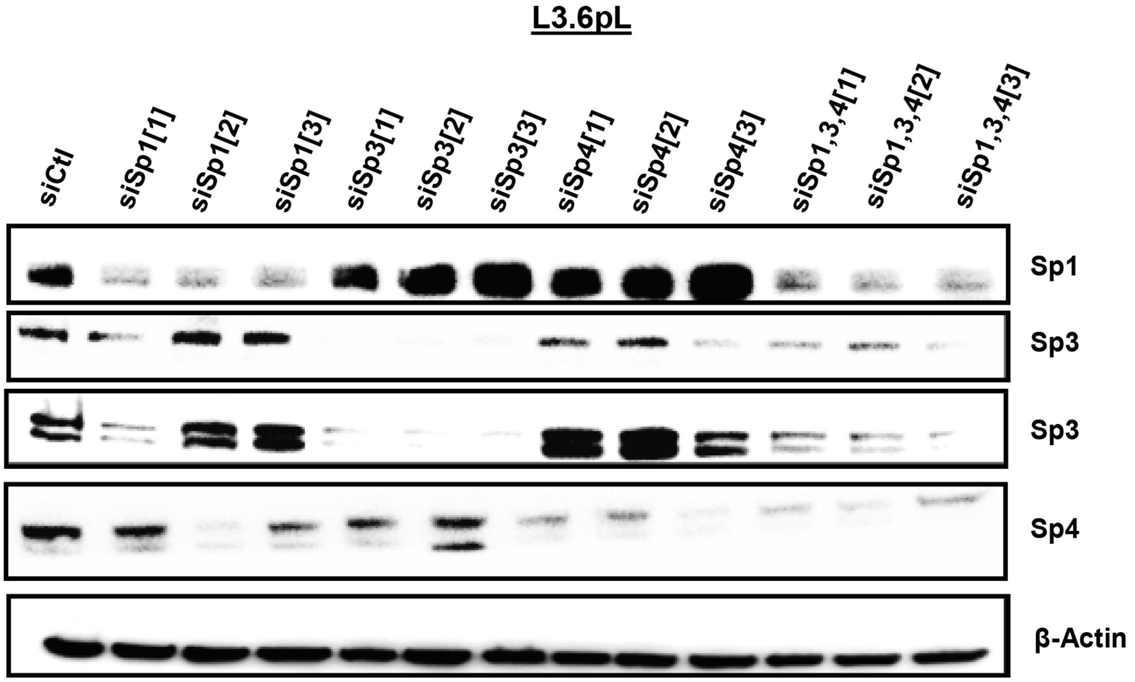

Supplementary Figure S1: Effects of different Sp oligonucleotides. Multiple Sp oligonucleotides targeted against Sp1,Sp3, and Sp4 were transfected into L3.6pL cells and whole cell lysates were analyzed by western blots. Sp1(1), Sp3(1), and Sp4(1) were used in subsequent knockdown experiments.

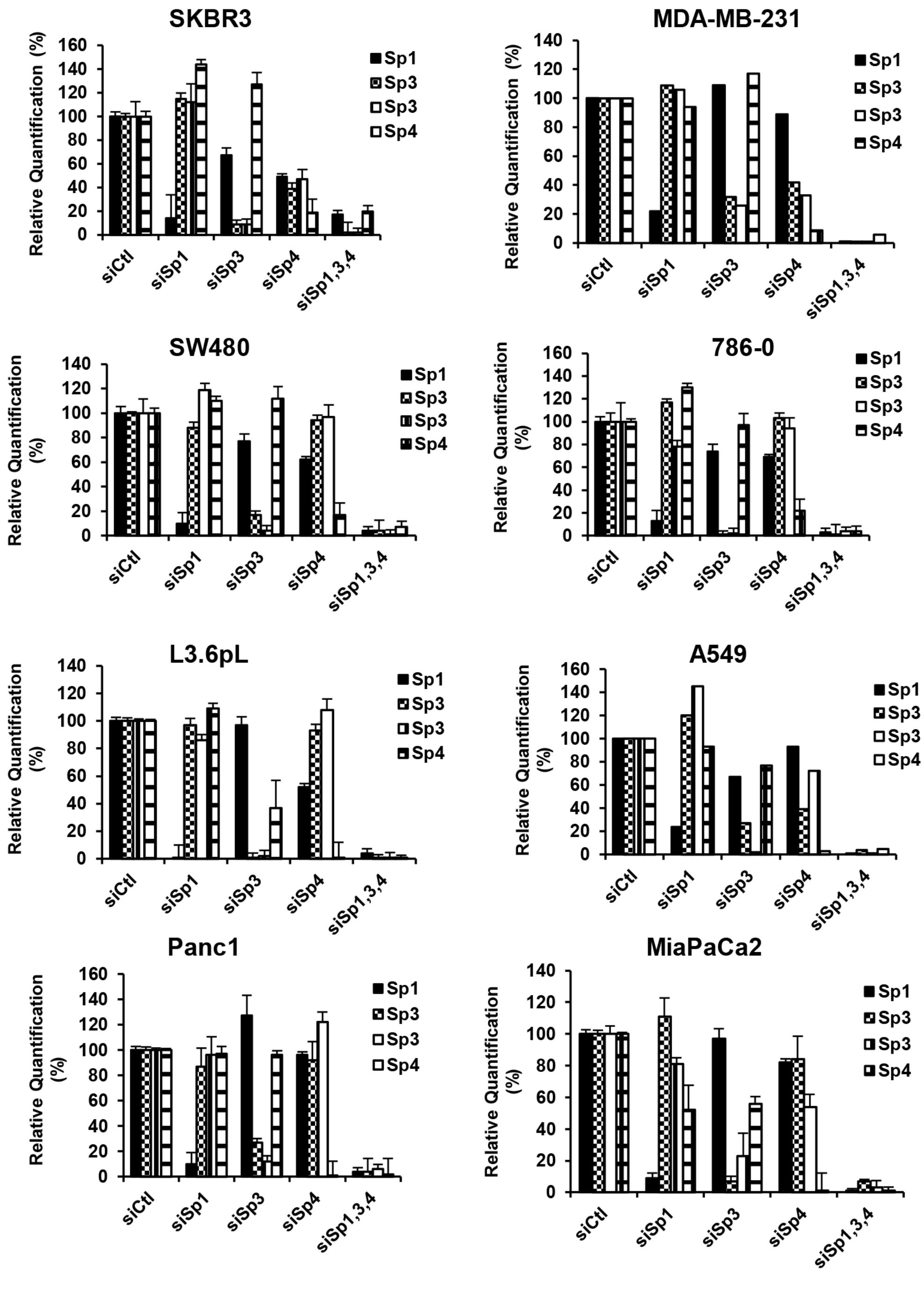

Supplementary Figure S2: Quantitation of Sp downregulation by RNAi. The downregulation of Sp1, Sp3 and Sp4 by RNAi (Figure 1) was quantitated by determining blot densities relative to β-actin control (set at 100%). Values for various proteins are means ±SE for 3 replicate determinations except for SKBR3 and A549 cells where the values are means of 2 determinations.

Supplementary Table S1: Sp1-regulated associated with growth inhibition, cell death and inhibition of migration/ invasion after Sp1 knockdown: expected and inversely regulated genes.

See Supplementary File 1

Supplementary Table S2: Sp3-regulated associated with growth inhibition, cell death and inhibition of migration/ invasion after Sp3 knockdown: expected and inversely regulated genes.

See Supplementary File 2

Supplementary Table S3: Sp4-regulated associated with growth inhibition, cell death and inhibition of migration/ invasion after Sp4 knockdown: expected and inversely regulated genes.

See Supplementary File 3
