# Supplementary material for: Specificity protein (Sp) transcription factors Sp1, Sp3 and Sp4 are non-oncogene addiction genes in cancer cells
Source: Oncotarget. 2016 Mar 5;7(16):22245–56. doi: 10.18632/oncotarget.7925 (PMC5008359; doi:10.18632/oncotarget.7925)
Supplement: Supplementary file 2 [file oncotarget-07-22245-s002.docx]

Supplemental Table S3. Sp4-regulated associated with growth inhibition, cell death and inhibition of migration/invasion after Sp4 knockdown: expected and inversely regulated genes.

A.

| **Expected** | | | | **Inversely related** | | | |
| --- | --- | --- | --- | --- | --- | --- | --- |
| **decreased cell proliferation** | | | | **increased cell proliferation** | | | |
| **up-genes (68)** | fold change | **down genes (447)** | fold change | **up-genes (131)** | fold change | **down genes (221)** | fold change |
| **TXNIP** | 4.646 | **EGR1** | -13.207 | **LMNA** | 10.206 | **DCBLD2** | -6.867 |
| **CDCA4** | 3.479 | **RRM2** | -10.183 | **NCOR2** | 3.634 | **STAT1** | -5.293 |
| **RNH1** | 3.269 | **FOS** | -5.995 | **HMGB1** | 3.379 | **PLOD2** | -4.87 |
| **HMOX1** | 3.189 | **PDE5A** | -5.765 | **C1QBP** | 3.093 | **LIPA** | -4.506 |
| **NUMA1** | 2.972 | **CTGF** | -5.348 | **HMGB2** | 3.081 | **ARRDC3** | -3.84 |
| **EI24** | 2.961 | **BIRC3** | -4.833 | **RCE1** | 3.003 | **EIF4G2** | -3.785 |
| **TCF3** | 2.595 | **NCSTN** | -4.492 | **H2AFX** | 2.91 | **ITGA6** | -3.665 |
| **MSX1** | 2.469 | **DKK1** | -4.291 | **CBX7** | 2.91 | **TGFBR3** | -3.607 |
| **MXD4** | 2.454 | **DUSP6** | -4.027 | **SERPINH1** | 2.644 | **PNPT1** | -3.293 |
| **UCP2** | 2.453 | **LIN28B** | -3.856 | **PTPMT1** | 2.629 | **TRIB1** | -3.286 |
| **EIF1** | 2.369 | **GOLM1** | -3.835 | **SUMO2** | 2.39 | **AKAP12** | -3.279 |
| **TIMP2** | 2.349 | **RPS6KA3** | -3.8 | **LRPAP1** | 2.336 | **LIFR** | -3.193 |
| **RALBP1** | 2.288 | **BIRC2** | -3.744 | **NOLC1** | 2.264 | **TFPI** | -3.143 |
| **GPR56** | 2.251 | **IL6ST** | -3.727 | **CDCA5** | 2.226 | **SPRY1** | -3.016 |
| **GEMIN2** | 2.233 | **AFAP1** | -3.709 | **NOP58** | 2.209 | **EGR2** | -3.01 |
| **SH2B3** | 2.221 | **DDX58** | -3.697 | **ARF1** | 2.207 | **CHEK2** | -2.983 |
| **DNAJA3** | 2.179 | **CCNB1** | -3.663 | **ACIN1** | 2.203 | **B3GNT5** | -2.962 |
| **ACTN4** | 2.107 | **CXCL8** | -3.59 | **EMP1** | 2.173 | **ABCC5** | -2.869 |
| **SREBF1** | 2.099 | **TFRC** | -3.539 | **CDC25B** | 2.173 | **ASPH** | -2.85 |
| **MED25** | 2.021 | **B4GALT6** | -3.49 | **CSF2RA** | 2.166 | **UBE2D3** | -2.821 |
| **NFKBIB** | 2.012 | **PPP3CB** | -3.478 | **ATP5G1** | 2.148 | **STX2** | -2.795 |
| **NUPR1** | 1.962 | **ADAR** | -3.472 | **DEPTOR** | 2.125 | **ATG5** | -2.746 |
| **GADD45A** | 1.958 | **FNDC3B** | -3.381 | **ANTXR1** | 2.124 | **PROS1** | -2.711 |
| **SIGIRR** | 1.929 | **IER3** | -3.343 | **MCM8** | 2.087 | **CASP7** | -2.665 |
| **NUP62** | 1.926 | **AKIRIN1** | -3.304 | **ETS1** | 2.086 | **CRLF3** | -2.598 |
| **HSPA1A/HSPA1B** | 1.919 | **AGO2** | -3.198 | **EWSR1** | 2.075 | **SESN1** | -2.596 |
| **PCBP4** | 1.892 | **CYR61** | -3.158 | **MED1** | 2.069 | **FRMD6** | -2.595 |
| **DAXX** | 1.89 | **PLAU** | -3.072 | **TBP** | 2.041 | **CTNNA1** | -2.582 |
| **MUL1** | 1.885 | **MCL1** | -3.012 | **SET** | 2.028 | **TGFBR2** | -2.574 |
| **CHD5** | 1.876 | **DICER1** | -2.978 | **RPS15A** | 2.014 | **RRAS** | -2.572 |
| **ATF5** | 1.867 | **SERPINE1** | -2.973 | **RARA** | 2.005 | **KRIT1** | -2.557 |
| **DNAJB6** | 1.859 | **LBR** | -2.959 | **YWHAG** | 1.998 | **NDFIP1** | -2.509 |
| **BCCIP** | 1.849 | **F2R** | -2.95 | **H3F3A/H3F3B** | 1.971 | **ULK2** | -2.479 |
| **TSPYL2** | 1.838 | **CTNNB1** | -2.941 | **TBC1D8** | 1.968 | **SPRY2** | -2.451 |
| **DIABLO** | 1.82 | **VCL** | -2.927 | **NACA** | 1.966 | **SPRY4** | -2.435 |
| **DACT3** | 1.801 | **ERCC1** | -2.888 | **MKL1** | 1.964 | **WIPF1** | -2.423 |
| **BMF** | 1.763 | **F2RL1** | -2.884 | **ISG15** | 1.962 | **BMPR2** | -2.396 |
| **UXT** | 1.762 | **E2F5** | -2.873 | **CREB1** | 1.947 | **WTAP** | -2.382 |
| **COMMD5** | 1.759 | **CD44** | -2.866 | **TPD52** | 1.936 | **RHOB** | -2.37 |
| **MTA1** | 1.752 | **DDAH1** | -2.769 | **CDK6** | 1.935 | **ANXA7** | -2.367 |
| **EEF1B2** | 1.731 | **ZNF451** | -2.743 | **ULK1** | 1.915 | **PPP2R1B** | -2.359 |
| **SSTR2** | 1.72 | **PRKCA** | -2.726 | **S100A4** | 1.906 | **YWHAZ** | -2.351 |
| **GPS2** | 1.703 | **GCNT2** | -2.721 | **CBS/LOC102724560** | 1.897 | **PCYOX1** | -2.344 |
| **FBLN1** | 1.69 | **ERO1L** | -2.695 | **PIP4K2B** | 1.893 | **H2AFY** | -2.337 |
| **STRA13** | 1.684 | **PTHLH** | -2.695 | **CORO1B** | 1.875 | **RBL2** | -2.325 |
| **ERF** | 1.673 | **GFM1** | -2.678 | **MPRIP** | 1.865 | **KIFAP3** | -2.307 |
| **FBXO2** | 1.671 | **DOCK1** | -2.674 | **E2F4** | 1.863 | **CTH** | -2.295 |
| **BNIPL** | 1.653 | **GCH1** | -2.667 | **TFF3** | 1.851 | **SEC23A** | -2.293 |
| **HRASLS** | 1.646 | **NBN** | -2.657 | **UBE2C** | 1.839 | **TAP1** | -2.289 |
| **FKTN** | 1.635 | **ERO1LB** | -2.639 | **HNRNPA2B1** | 1.838 | **RASA1** | -2.286 |
| **ABCC3** | 1.625 | **P4HA1** | -2.623 | **CRCP** | 1.833 | **CAT** | -2.286 |
| **DNAJB2** | 1.616 | **STT3B** | -2.621 | **LEP** | 1.833 | **TBK1** | -2.28 |
| **GABPB2** | 1.591 | **CITED2** | -2.617 | **HOXC6** | 1.821 | **NFE2L2** | -2.278 |
| **EMP3** | 1.587 | **C1GALT1** | -2.611 | **RANBP1** | 1.82 | **PHLDA1** | -2.25 |
| **ARID1A** | 1.575 | **ARFGAP3** | -2.61 | **FBRS** | 1.816 | **FAM188A** | -2.249 |
| **PDCD4** | 1.573 | **HSPA4** | -2.6 | **ID3** | 1.814 | **ERRFI1** | -2.248 |
| **TSPO** | 1.561 | **TXNDC5** | -2.591 | **TXLNA** | 1.81 | **ROCK1** | -2.233 |
| **TOP3A** | 1.56 | **RAN** | -2.569 | **POLR2L** | 1.804 | **P4HA2** | -2.217 |
| **RPL26** | 1.555 | **PLK2** | -2.565 | **PFDN5** | 1.804 | **CAPRIN1** | -2.198 |
| **CCDC85B** | 1.553 | **SGK1** | -2.554 | **DOHH** | 1.788 | **RB1** | -2.193 |
| **LILRB1** | 1.548 | **LAMC1** | -2.526 | **DCLRE1A** | 1.777 | **RND3** | -2.173 |
| **SRM** | 1.548 | **JAK1** | -2.514 | **NDEL1** | 1.764 | **PLXNA1** | -2.152 |
| **ATPIF1** | 1.54 | **NUP98** | -2.507 | **NUDT1** | 1.759 | **RNASEL** | -2.147 |
| **NRG2** | 1.538 | **PIGN** | -2.499 | **IL18** | 1.758 | **CAST** | -2.143 |
| **RBM10** | 1.531 | **DDX5** | -2.487 | **RPS9** | 1.749 | **FBXO11** | -2.14 |
| **TNFRSF25** | 1.529 | **ITGB1** | -2.481 | **PPL** | 1.749 | **IFNL2** | -2.139 |
| **MXI1** | 1.525 | **SPTBN1** | -2.478 | **STX3** | 1.739 | **G3BP1** | -2.137 |
| **DBI** | 1.501 | **MUC1** | -2.466 | **CEP131** | 1.737 | **TRIM24** | -2.135 |
|  |  | **ITGAV** | -2.466 | **NSMF** | 1.72 | **JUNB** | -2.12 |
|  |  | **STAT5B** | -2.464 | **MCM10** | 1.718 | **RBBP4** | -2.111 |
|  |  | **TNS3** | -2.454 | **AHNAK** | 1.712 | **GEM** | -2.102 |
|  |  | **TDG** | -2.451 | **NAA38** | 1.7 | **AKTIP** | -2.1 |
|  |  | **ICAM1** | -2.449 | **SRC** | 1.692 | **ROCK2** | -2.096 |
|  |  | **NFKB1** | -2.435 | **SPTAN1** | 1.69 | **NFIB** | -2.09 |
|  |  | **PIK3CA** | -2.434 | **BASP1** | 1.688 | **SERINC3** | -2.087 |
|  |  | **CDC16** | -2.431 | **MCM4** | 1.683 | **B3GNT2** | -2.08 |
|  |  | **FZD7** | -2.431 | **DUSP12** | 1.67 | **BTG2** | -2.047 |
|  |  | **RYK** | -2.422 | **ARHGEF1** | 1.658 | **MXD1** | -2.047 |
|  |  | **MFN1** | -2.409 | **DTL** | 1.647 | **BHLHE40** | -2.044 |
|  |  | **MAP3K7** | -2.4 | **LGALS1** | 1.644 | **PARG** | -2.039 |
|  |  | **RECK** | -2.393 | **CD320** | 1.643 | **TFPI2** | -2.025 |
|  |  | **HIF1A** | -2.374 | **FEN1** | 1.635 | **DDX3X** | -2.022 |
|  |  | **TP53BP1** | -2.37 | **GJC1** | 1.634 | **ASNS** | -2.018 |
|  |  | **DLGAP5** | -2.37 | **DAPP1** | 1.633 | **RAD17** | -2.016 |
|  |  | **NFYA** | -2.367 | **PPP1R15A** | 1.632 | **CUL2** | -2.005 |
|  |  | **EOMES** | -2.36 | **POLR2F** | 1.628 | **SMYD2** | -1.999 |
|  |  | **NASP** | -2.349 | **IGBP1** | 1.626 | **CASP3** | -1.996 |
|  |  | **HMGCR** | -2.348 | **PTP4A2** | 1.624 | **RNF14** | -1.995 |
|  |  | **CYP2J2** | -2.339 | **THRAP3** | 1.622 | **RCHY1** | -1.971 |
|  |  | **PHIP** | -2.336 | **MDK** | 1.619 | **ZMAT3** | -1.967 |
|  |  | **CD2AP** | -2.323 | **SP1** | 1.601 | **ENPP1** | -1.966 |
|  |  | **ARFGEF1** | -2.293 | **EEF1D** | 1.591 | **PKP2** | -1.924 |
|  |  | **EXTL3** | -2.291 | **CDC20** | 1.59 | **PHF14** | -1.919 |
|  |  | **KRAS** | -2.288 | **ARHGEF2** | 1.588 | **ADAMTS1** | -1.918 |
|  |  | **CCPG1** | -2.286 | **CDT1** | 1.582 | **TRIB3** | -1.914 |
|  |  | **EPCAM** | -2.282 | **FANCD2** | 1.58 | **TOM1L1** | -1.906 |
|  |  | **ACER3** | -2.262 | **PSMA4** | 1.579 | **SMAD4** | -1.903 |
|  |  | **DNM1L** | -2.261 | **SHC1** | 1.578 | **HIAT1** | -1.899 |
|  |  | **CSNK1G3** | -2.261 | **MXD3** | 1.577 | **PPP2CA** | -1.896 |
|  |  | **PRKAA1** | -2.258 | **DIXDC1** | 1.577 | **PEX2** | -1.883 |
|  |  | **NRG1** | -2.255 | **PTMA** | 1.576 | **SMARCA1** | -1.882 |
|  |  | **LGR4** | -2.254 | **HSF1** | 1.576 | **TJP1** | -1.882 |
|  |  | **USO1** | -2.249 | **PTGDS** | 1.568 | **SYNJ2BP** | -1.879 |
|  |  | **PDE4D** | -2.248 | **CDK4** | 1.568 | **CCNG2** | -1.87 |
|  |  | **ARL5A** | -2.235 | **S100A6** | 1.563 | **MAP3K2** | -1.87 |
|  |  | **SLC20A1** | -2.213 | **PPIB** | 1.563 | **DYRK1A** | -1.869 |
|  |  | **BTRC** | -2.198 | **RALGDS** | 1.553 | **CSE1L** | -1.869 |
|  |  | **HIF1AN** | -2.189 | **TNFSF14** | 1.553 | **NACC2** | -1.866 |
|  |  | **MAP4K4** | -2.187 | **PCK2** | 1.55 | **DNER** | -1.863 |
|  |  | **TOP2A** | -2.183 | **HGS** | 1.548 | **DLG1** | -1.861 |
|  |  | **EPAS1** | -2.164 | **PIDD1** | 1.546 | **TP53INP1** | -1.847 |
|  |  | **DSG2** | -2.162 | **SNX12** | 1.546 | **MERTK** | -1.842 |
|  |  | **USP47** | -2.16 | **CYBA** | 1.544 | **KIF23** | -1.839 |
|  |  | **EIF5A2** | -2.158 | **TBX1** | 1.542 | **MORC3** | -1.834 |
|  |  | **NAMPT** | -2.151 | **mir-130** | 1.539 | **DYNC1H1** | -1.833 |
|  |  | **USP18** | -2.15 | **AKIRIN2** | 1.539 | **CAPRIN2** | -1.826 |
|  |  | **TPR** | -2.15 | **CNTFR** | 1.537 | **KLF10** | -1.825 |
|  |  | **MAPK13** | -2.149 | **CISD1** | 1.537 | **VLDLR** | -1.817 |
|  |  | **YES1** | -2.148 | **SLC7A5** | 1.533 | **EIF2AK2** | -1.814 |
|  |  | **PIK3CB** | -2.146 | **SLC44A4** | 1.53 | **STARD13** | -1.81 |
|  |  | **DDX17** | -2.142 | **CKS1B** | 1.526 | **PTPN14** | -1.791 |
|  |  | **FAT4** | -2.128 | **ACACA** | 1.525 | **ARID4A** | -1.791 |
|  |  | **SLC39A6** | -2.126 | **PIN1** | 1.519 | **IFNL3** | -1.79 |
|  |  | **ILK** | -2.118 | **TIMELESS** | 1.513 | **TES** | -1.785 |
|  |  | **PIK3C2A** | -2.107 | **MFGE8** | 1.512 | **PTPN12** | -1.782 |
|  |  | **CD46** | -2.106 | **NSA2** | 1.512 | **GPNMB** | -1.779 |
|  |  | **XRCC5** | -2.091 | **COPS2** | 1.511 | **ARID4B** | -1.773 |
|  |  | **HSPD1** | -2.089 | **ORAI1** | 1.508 | **CSNK1A1** | -1.772 |
|  |  | **KIF11** | -2.088 | **SRF** | 1.503 | **USP16** | -1.77 |
|  |  | **TNFRSF10B** | -2.08 | **SH2B1** | 1.502 | **HOXA5** | -1.757 |
|  |  | **PRPS2** | -2.077 | **SHFM1** | 1.501 | **CTSV** | -1.756 |
|  |  | **TNFAIP8** | -2.074 |  |  | **CPEB1** | -1.755 |
|  |  | **ZMYM2** | -2.063 |  |  | **CYLD** | -1.742 |
|  |  | **RGPD4 (includes others)** | -2.061 |  |  | **NAB1** | -1.733 |
|  |  | **RAB28** | -2.06 |  |  | **DUSP5** | -1.729 |
|  |  | **ANGPTL4** | -2.057 |  |  | **RHOH** | -1.729 |
|  |  | **MYOF** | -2.056 |  |  | **PDS5B** | -1.72 |
|  |  | **RPA1** | -2.034 |  |  | **APBB2** | -1.714 |
|  |  | **ITGA2** | -2.025 |  |  | **CDK8** | -1.71 |
|  |  | **CXCL1** | -2.023 |  |  | **MCRS1** | -1.705 |
|  |  | **BMPR1A** | -2.011 |  |  | **LIMK1** | -1.704 |
|  |  | **CDC14B** | -2.011 |  |  | **CAV1** | -1.702 |
|  |  | **RALA** | -2.005 |  |  | **TOP1** | -1.702 |
|  |  | **DIDO1** | -2.004 |  |  | **SGPL1** | -1.701 |
|  |  | **SUV420H1** | -2.003 |  |  | **BACH2** | -1.697 |
|  |  | **AMD1** | -2 |  |  | **AJUBA** | -1.693 |
|  |  | **GAD1** | -1.997 |  |  | **DVL1** | -1.693 |
|  |  | **TADA3** | -1.997 |  |  | **CD83** | -1.691 |
|  |  | **REL** | -1.995 |  |  | **SPRED1** | -1.69 |
|  |  | **ASCC3** | -1.993 |  |  | **ITPR1** | -1.689 |
|  |  | **PRKAR1A** | -1.993 |  |  | **ATG7** | -1.687 |
|  |  | **CUL4A** | -1.987 |  |  | **RNF111** | -1.684 |
|  |  | **ROR1** | -1.987 |  |  | **ABLIM1** | -1.683 |
|  |  | **PLS3** | -1.983 |  |  | **VMP1** | -1.683 |
|  |  | **HNRNPK** | -1.978 |  |  | **PAQR3** | -1.679 |
|  |  | **SSR1** | -1.975 |  |  | **PHC3** | -1.672 |
|  |  | **TSNAX** | -1.971 |  |  | **PPM1A** | -1.669 |
|  |  | **STAT4** | -1.968 |  |  | **RGN** | -1.664 |
|  |  | **MINA** | -1.96 |  |  | **ST7L** | -1.662 |
|  |  | **NFKBIA** | -1.955 |  |  | **FOXN3** | -1.658 |
|  |  | **TGIF1** | -1.951 |  |  | **CASK** | -1.656 |
|  |  | **ACVR1** | -1.951 |  |  | **ABCC1** | -1.655 |
|  |  | **ATP7A** | -1.948 |  |  | **NOTCH2** | -1.649 |
|  |  | **ADAM17** | -1.945 |  |  | **TP53BP2** | -1.644 |
|  |  | **SDCBP** | -1.94 |  |  | **APPL1** | -1.637 |
|  |  | **KDM5B** | -1.939 |  |  | **IFIT3** | -1.613 |
|  |  | **ARID3A** | -1.939 |  |  | **BAX** | -1.608 |
|  |  | **OPTN** | -1.936 |  |  | **ATP6V0A2** | -1.606 |
|  |  | **KIF2A** | -1.931 |  |  | **ADIPOR1** | -1.601 |
|  |  | **SEMA4D** | -1.931 |  |  | **TSG101** | -1.595 |
|  |  | **VEGFC** | -1.925 |  |  | **IFNAR1** | -1.595 |
|  |  | **OPA1** | -1.923 |  |  | **FBXL2** | -1.594 |
|  |  | **WWTR1** | -1.923 |  |  | **BCAN** | -1.591 |
|  |  | **ILF3** | -1.919 |  |  | **ZFP36** | -1.591 |
|  |  | **CENPF** | -1.915 |  |  | **KDM3B** | -1.591 |
|  |  | **ADAM9** | -1.909 |  |  | **KLK3** | -1.59 |
|  |  | **C1GALT1C1** | -1.908 |  |  | **RANBP9** | -1.589 |
|  |  | **DVL3** | -1.905 |  |  | **RAP1GAP** | -1.588 |
|  |  | **NFAT5** | -1.904 |  |  | **STEAP3** | -1.586 |
|  |  | **MAPK1** | -1.901 |  |  | **LATS1** | -1.58 |
|  |  | **NAE1** | -1.898 |  |  | **WARS** | -1.579 |
|  |  | **MEMO1** | -1.896 |  |  | **HAS3** | -1.579 |
|  |  | **IL13RA1** | -1.895 |  |  | **MEN1** | -1.579 |
|  |  | **ELMO2** | -1.885 |  |  | **PMEPA1** | -1.574 |
|  |  | **NEDD4L** | -1.883 |  |  | **CREG1** | -1.572 |
|  |  | **NFIA** | -1.882 |  |  | **TANK** | -1.571 |
|  |  | **EXOC5** | -1.881 |  |  | **CD40** | -1.568 |
|  |  | **TEAD2** | -1.88 |  |  | **CCNL2** | -1.566 |
|  |  | **BPNT1** | -1.878 |  |  | **STK3** | -1.563 |
|  |  | **UBE2A** | -1.878 |  |  | **PLSCR1** | -1.562 |
|  |  | **CSRP1** | -1.874 |  |  | **LAT2** | -1.561 |
|  |  | **MSH2** | -1.867 |  |  | **RPS6KA2** | -1.56 |
|  |  | **PAK2** | -1.864 |  |  | **FGFRL1** | -1.556 |
|  |  | **PTPN3** | -1.864 |  |  | **PLCL2** | -1.556 |
|  |  | **RCAN1** | -1.858 |  |  | **TCF12** | -1.551 |
|  |  | **KPNA2** | -1.855 |  |  | **USP10** | -1.55 |
|  |  | **ABI2** | -1.855 |  |  | **HERC2** | -1.55 |
|  |  | **MALT1** | -1.853 |  |  | **TEX11** | -1.549 |
|  |  | **PTPN1** | -1.85 |  |  | **IQGAP1** | -1.548 |
|  |  | **BMI1** | -1.85 |  |  | **DAB2** | -1.548 |
|  |  | **APP** | -1.846 |  |  | **RASL10A** | -1.543 |
|  |  | **GLDC** | -1.843 |  |  | **CDK11B** | -1.539 |
|  |  | **MET** | -1.834 |  |  | **IFT88** | -1.535 |
|  |  | **WAPAL** | -1.832 |  |  | **PARK2** | -1.534 |
|  |  | **LCN2** | -1.83 |  |  | **SIX5** | -1.533 |
|  |  | **CCDC88A** | -1.827 |  |  | **RBBP7** | -1.531 |
|  |  | **API5** | -1.825 |  |  | **OAS3** | -1.528 |
|  |  | **LDHA** | -1.823 |  |  | **CXADR** | -1.527 |
|  |  | **FBXW11** | -1.823 |  |  | **KCND2** | -1.527 |
|  |  | **AHR** | -1.82 |  |  | **STRN** | -1.527 |
|  |  | **RASGRP3** | -1.818 |  |  | **PHLDA2** | -1.525 |
|  |  | **IMPACT** | -1.816 |  |  | **KLF13** | -1.52 |
|  |  | **PTPN13** | -1.816 |  |  | **CTBP2** | -1.512 |
|  |  | **NR2C2** | -1.813 |  |  | **FBXW7** | -1.511 |
|  |  | **CTSB** | -1.812 |  |  | **NPDC1** | -1.511 |
|  |  | **RUVBL1** | -1.811 |  |  | **EEF1A1** | -1.506 |
|  |  | **STAT3** | -1.806 |  |  | **MIB1** | -1.506 |
|  |  | **KMT2C** | -1.792 |  |  | **WDR48** | -1.506 |
|  |  | **ANXA2** | -1.791 |  |  | **IFNGR1** | -1.504 |
|  |  | **NPC1** | -1.791 |  |  | **IFT74** | -1.502 |
|  |  | **C8orf44-SGK3/SGK3** | -1.79 |  |  | **TLR6** | -1.501 |
|  |  | **BCAT1** | -1.789 |  |  |  |  |
|  |  | **UFL1** | -1.789 |  |  |  |  |
|  |  | **STEAP2** | -1.784 |  |  |  |  |
|  |  | **FUT4** | -1.782 |  |  |  |  |
|  |  | **FOXQ1** | -1.778 |  |  |  |  |
|  |  | **ALCAM** | -1.776 |  |  |  |  |
|  |  | **SPAST** | -1.775 |  |  |  |  |
|  |  | **NOC3L** | -1.773 |  |  |  |  |
|  |  | **PDPK1** | -1.769 |  |  |  |  |
|  |  | **PRMT6** | -1.769 |  |  |  |  |
|  |  | **KIAA1429** | -1.766 |  |  |  |  |
|  |  | **STK38L** | -1.765 |  |  |  |  |
|  |  | **PFKM** | -1.761 |  |  |  |  |
|  |  | **IDE** | -1.76 |  |  |  |  |
|  |  | **WLS** | -1.756 |  |  |  |  |
|  |  | **AURKA** | -1.755 |  |  |  |  |
|  |  | **ARNTL** | -1.749 |  |  |  |  |
|  |  | **TTC5** | -1.748 |  |  |  |  |
|  |  | **ERBB2** | -1.74 |  |  |  |  |
|  |  | **LEPR** | -1.739 |  |  |  |  |
|  |  | **SLC12A2** | -1.739 |  |  |  |  |
|  |  | **PPIA** | -1.736 |  |  |  |  |
|  |  | **ALDOA** | -1.736 |  |  |  |  |
|  |  | **STAM2** | -1.734 |  |  |  |  |
|  |  | **mir-21** | -1.733 |  |  |  |  |
|  |  | **ADM** | -1.732 |  |  |  |  |
|  |  | **IGF1R** | -1.729 |  |  |  |  |
|  |  | **MSI2** | -1.729 |  |  |  |  |
|  |  | **MAP2K5** | -1.728 |  |  |  |  |
|  |  | **POT1** | -1.727 |  |  |  |  |
|  |  | **UBR5** | -1.723 |  |  |  |  |
|  |  | **GNAQ** | -1.722 |  |  |  |  |
|  |  | **FAIM** | -1.72 |  |  |  |  |
|  |  | **EPS8** | -1.716 |  |  |  |  |
|  |  | **FGFR1OP** | -1.716 |  |  |  |  |
|  |  | **GLB1** | -1.715 |  |  |  |  |
|  |  | **STK39** | -1.713 |  |  |  |  |
|  |  | **NRAS** | -1.713 |  |  |  |  |
|  |  | **CCT2** | -1.711 |  |  |  |  |
|  |  | **CHUK** | -1.708 |  |  |  |  |
|  |  | **CTTN** | -1.707 |  |  |  |  |
|  |  | **AMACR** | -1.699 |  |  |  |  |
|  |  | **RBBP9** | -1.699 |  |  |  |  |
|  |  | **AKR1C3** | -1.698 |  |  |  |  |
|  |  | **YAP1** | -1.696 |  |  |  |  |
|  |  | **CDK1** | -1.693 |  |  |  |  |
|  |  | **HOXB3** | -1.691 |  |  |  |  |
|  |  | **LTB** | -1.691 |  |  |  |  |
|  |  | **PTPN11** | -1.688 |  |  |  |  |
|  |  | **RECQL** | -1.687 |  |  |  |  |
|  |  | **FDFT1** | -1.687 |  |  |  |  |
|  |  | **MSH3** | -1.686 |  |  |  |  |
|  |  | **HGF** | -1.685 |  |  |  |  |
|  |  | **NFKB2** | -1.685 |  |  |  |  |
|  |  | **HSP90B1** | -1.683 |  |  |  |  |
|  |  | **BIRC6** | -1.682 |  |  |  |  |
|  |  | **CDK13** | -1.679 |  |  |  |  |
|  |  | **ACTB** | -1.678 |  |  |  |  |
|  |  | **PALLD** | -1.676 |  |  |  |  |
|  |  | **FKBP1A** | -1.676 |  |  |  |  |
|  |  | **SMC3** | -1.675 |  |  |  |  |
|  |  | **PLD1** | -1.675 |  |  |  |  |
|  |  | **BUB3** | -1.674 |  |  |  |  |
|  |  | **TGIF2** | -1.674 |  |  |  |  |
|  |  | **CD164** | -1.673 |  |  |  |  |
|  |  | **CUL1** | -1.673 |  |  |  |  |
|  |  | **MDM2** | -1.672 |  |  |  |  |
|  |  | **ADNP2** | -1.671 |  |  |  |  |
|  |  | **CCNA2** | -1.67 |  |  |  |  |
|  |  | **NCOA2** | -1.666 |  |  |  |  |
|  |  | **KIF3A** | -1.666 |  |  |  |  |
|  |  | **ABCB7** | -1.665 |  |  |  |  |
|  |  | **TASP1** | -1.664 |  |  |  |  |
|  |  | **TNF** | -1.663 |  |  |  |  |
|  |  | **TIRAP** | -1.662 |  |  |  |  |
|  |  | **FUT8** | -1.661 |  |  |  |  |
|  |  | **NUMB** | -1.66 |  |  |  |  |
|  |  | **HDAC8** | -1.659 |  |  |  |  |
|  |  | **RIPK1** | -1.655 |  |  |  |  |
|  |  | **CASP8** | -1.652 |  |  |  |  |
|  |  | **RAB1A** | -1.648 |  |  |  |  |
|  |  | **EXOC4** | -1.647 |  |  |  |  |
|  |  | **HLA-DMB** | -1.646 |  |  |  |  |
|  |  | **PRKX** | -1.642 |  |  |  |  |
|  |  | **COPS4** | -1.639 |  |  |  |  |
|  |  | **MAP2K1** | -1.639 |  |  |  |  |
|  |  | **SLC30A6** | -1.638 |  |  |  |  |
|  |  | **OSBPL1A** | -1.637 |  |  |  |  |
|  |  | **HEYL** | -1.637 |  |  |  |  |
|  |  | **CDK2** | -1.635 |  |  |  |  |
|  |  | **DNAJA2** | -1.635 |  |  |  |  |
|  |  | **PSMC2** | -1.634 |  |  |  |  |
|  |  | **CD151** | -1.634 |  |  |  |  |
|  |  | **VGF** | -1.633 |  |  |  |  |
|  |  | **EIF4A1** | -1.632 |  |  |  |  |
|  |  | **PKM** | -1.63 |  |  |  |  |
|  |  | **RAB11A** | -1.629 |  |  |  |  |
|  |  | **EPHB2** | -1.628 |  |  |  |  |
|  |  | **CDK5** | -1.627 |  |  |  |  |
|  |  | **EBI3** | -1.623 |  |  |  |  |
|  |  | **TAF9B** | -1.622 |  |  |  |  |
|  |  | **MAPK6** | -1.62 |  |  |  |  |
|  |  | **CCNE1** | -1.619 |  |  |  |  |
|  |  | **PIK3R1** | -1.619 |  |  |  |  |
|  |  | **RAD50** | -1.617 |  |  |  |  |
|  |  | **PGK1** | -1.616 |  |  |  |  |
|  |  | **RHOQ** | -1.616 |  |  |  |  |
|  |  | **RAB5A** | -1.615 |  |  |  |  |
|  |  | **FGF2** | -1.613 |  |  |  |  |
|  |  | **IL11RA** | -1.611 |  |  |  |  |
|  |  | **CCND1** | -1.609 |  |  |  |  |
|  |  | **USP1** | -1.608 |  |  |  |  |
|  |  | **TTLL7** | -1.608 |  |  |  |  |
|  |  | **DMTF1** | -1.607 |  |  |  |  |
|  |  | **ARFGEF2** | -1.607 |  |  |  |  |
|  |  | **AGO4** | -1.606 |  |  |  |  |
|  |  | **KIF20B** | -1.605 |  |  |  |  |
|  |  | **MAP3K4** | -1.604 |  |  |  |  |
|  |  | **SYNM** | -1.604 |  |  |  |  |
|  |  | **ALG14** | -1.603 |  |  |  |  |
|  |  | **MYD88** | -1.602 |  |  |  |  |
|  |  | **MAPK9** | -1.601 |  |  |  |  |
|  |  | **SLC52A1** | -1.601 |  |  |  |  |
|  |  | **BTN3A1** | -1.601 |  |  |  |  |
|  |  | **KIF3C** | -1.6 |  |  |  |  |
|  |  | **EEA1** | -1.599 |  |  |  |  |
|  |  | **GLCE** | -1.597 |  |  |  |  |
|  |  | **EXT1** | -1.596 |  |  |  |  |
|  |  | **ECT2** | -1.596 |  |  |  |  |
|  |  | **DDR1** | -1.595 |  |  |  |  |
|  |  | **HUWE1** | -1.594 |  |  |  |  |
|  |  | **TACC2** | -1.593 |  |  |  |  |
|  |  | **SRSF5** | -1.589 |  |  |  |  |
|  |  | **TRAF5** | -1.589 |  |  |  |  |
|  |  | **RAP1B** | -1.588 |  |  |  |  |
|  |  | **CDH1** | -1.587 |  |  |  |  |
|  |  | **ARHGAP24** | -1.587 |  |  |  |  |
|  |  | **SS18** | -1.585 |  |  |  |  |
|  |  | **PRDX3** | -1.584 |  |  |  |  |
|  |  | **PTPRA** | -1.583 |  |  |  |  |
|  |  | **SPP1** | -1.581 |  |  |  |  |
|  |  | **ATF6** | -1.579 |  |  |  |  |
|  |  | **LATS2** | -1.579 |  |  |  |  |
|  |  | **CALCRL** | -1.578 |  |  |  |  |
|  |  | **TCP1** | -1.577 |  |  |  |  |
|  |  | **MBP** | -1.575 |  |  |  |  |
|  |  | **AGGF1** | -1.574 |  |  |  |  |
|  |  | **CNOT6** | -1.572 |  |  |  |  |
|  |  | **FZD4** | -1.571 |  |  |  |  |
|  |  | **NEK2** | -1.57 |  |  |  |  |
|  |  | **FER** | -1.569 |  |  |  |  |
|  |  | **ASPM** | -1.567 |  |  |  |  |
|  |  | **METAP2** | -1.567 |  |  |  |  |
|  |  | **BMP6** | -1.566 |  |  |  |  |
|  |  | **STAM** | -1.566 |  |  |  |  |
|  |  | **E2F6** | -1.565 |  |  |  |  |
|  |  | **KCNMA1** | -1.565 |  |  |  |  |
|  |  | **PLAG1** | -1.565 |  |  |  |  |
|  |  | **HK2** | -1.563 |  |  |  |  |
|  |  | **TRIB2** | -1.563 |  |  |  |  |
|  |  | **MORF4L1** | -1.561 |  |  |  |  |
|  |  | **TEAD4** | -1.56 |  |  |  |  |
|  |  | **CLCN3** | -1.558 |  |  |  |  |
|  |  | **STK17A** | -1.558 |  |  |  |  |
|  |  | **CSNK2A1** | -1.557 |  |  |  |  |
|  |  | **CHKA** | -1.557 |  |  |  |  |
|  |  | **MAP1B** | -1.557 |  |  |  |  |
|  |  | **RALB** | -1.556 |  |  |  |  |
|  |  | **ACSL4** | -1.555 |  |  |  |  |
|  |  | **SHMT1** | -1.554 |  |  |  |  |
|  |  | **SMO** | -1.553 |  |  |  |  |
|  |  | **FASN** | -1.553 |  |  |  |  |
|  |  | **TNFRSF12A** | -1.55 |  |  |  |  |
|  |  | **LIG4** | -1.55 |  |  |  |  |
|  |  | **E2F3** | -1.549 |  |  |  |  |
|  |  | **TRPC1** | -1.549 |  |  |  |  |
|  |  | **CAPN2** | -1.548 |  |  |  |  |
|  |  | **ITGB4** | -1.547 |  |  |  |  |
|  |  | **PDGFC** | -1.546 |  |  |  |  |
|  |  | **ASAH1** | -1.545 |  |  |  |  |
|  |  | **CASP6** | -1.545 |  |  |  |  |
|  |  | **AGO3** | -1.545 |  |  |  |  |
|  |  | **RLIM** | -1.544 |  |  |  |  |
|  |  | **MCFD2** | -1.544 |  |  |  |  |
|  |  | **ID4** | -1.543 |  |  |  |  |
|  |  | **PLCE1** | -1.543 |  |  |  |  |
|  |  | **PRKD3** | -1.54 |  |  |  |  |
|  |  | **SLC30A1** | -1.539 |  |  |  |  |
|  |  | **HOXB4** | -1.538 |  |  |  |  |
|  |  | **PTGER4** | -1.538 |  |  |  |  |
|  |  | **ACER2** | -1.536 |  |  |  |  |
|  |  | **EGFR** | -1.535 |  |  |  |  |
|  |  | **CCNE2** | -1.535 |  |  |  |  |
|  |  | **CUL4B** | -1.533 |  |  |  |  |
|  |  | **PREP** | -1.532 |  |  |  |  |
|  |  | **DUSP10** | -1.531 |  |  |  |  |
|  |  | **JAK2** | -1.53 |  |  |  |  |
|  |  | **CCNDBP1** | -1.53 |  |  |  |  |
|  |  | **GOLPH3** | -1.529 |  |  |  |  |
|  |  | **UGCG** | -1.527 |  |  |  |  |
|  |  | **ARRB1** | -1.526 |  |  |  |  |
|  |  | **UBE2N** | -1.525 |  |  |  |  |
|  |  | **RBM3** | -1.524 |  |  |  |  |
|  |  | **RAC1** | -1.523 |  |  |  |  |
|  |  | **SLC12A4** | -1.522 |  |  |  |  |
|  |  | **KIF18A** | -1.522 |  |  |  |  |
|  |  | **COPS6** | -1.521 |  |  |  |  |
|  |  | **MMP25** | -1.521 |  |  |  |  |
|  |  | **LAMB1** | -1.521 |  |  |  |  |
|  |  | **VAPA** | -1.519 |  |  |  |  |
|  |  | **PRKCE** | -1.518 |  |  |  |  |
|  |  | **LCK** | -1.516 |  |  |  |  |
|  |  | **PRKD1** | -1.515 |  |  |  |  |
|  |  | **TIMP1** | -1.514 |  |  |  |  |
|  |  | **KDM2B** | -1.513 |  |  |  |  |
|  |  | **MSRA** | -1.512 |  |  |  |  |
|  |  | **CUX1** | -1.51 |  |  |  |  |
|  |  | **PDF** | -1.51 |  |  |  |  |
|  |  | **STK24** | -1.509 |  |  |  |  |
|  |  | **ARHGAP5** | -1.508 |  |  |  |  |
|  |  | **TPP2** | -1.505 |  |  |  |  |
|  |  | **AKAP13** | -1.503 |  |  |  |  |
|  |  | **EIF4E** | -1.502 |  |  |  |  |

B.

| **Expected** | | | | **Inversely related** | | | |
| --- | --- | --- | --- | --- | --- | --- | --- |
| **increased cell death** | | | | **decreased cell death** | | | |
| **up-genes (91)** | fold change | **down genes (401)** | fold change | **up-genes (101)** | fold change | **down genes (278)** | fold change |
| **TXNIP** | 4.646 | **RRM2** | -10.183 | **ARID3B** | 3.667 | **EGR1** | -13.207 |
| **NCOR2** | 3.634 | **SLC4A7** | -5.397 | **HMOX1** | 3.189 | **CD69** | -10.009 |
| **SMARCC1** | 3.407 | **CTGF** | -5.348 | **EI24** | 2.961 | **FOS** | -5.995 |
| **HMGB1** | 3.379 | **BIRC3** | -4.833 | **RGS10** | 2.924 | **PDE5A** | -5.765 |
| **HMGB2** | 3.081 | **NCSTN** | -4.492 | **STX8** | 2.918 | **STAT1** | -5.293 |
| **NUMA1** | 2.972 | **SCARB2** | -4.157 | **MEF2D** | 2.859 | **LIPA** | -4.506 |
| **H2AFX** | 2.91 | **DCAF7** | -4.08 | **SERPINH1** | 2.644 | **DKK1** | -4.291 |
| **HS1BP3** | 2.75 | **RRM2B** | -3.882 | **PTPMT1** | 2.629 | **DUSP6** | -4.027 |
| **TCF3** | 2.595 | **RPS6KA3** | -3.8 | **MSX1** | 2.469 | **ANTXR2** | -3.813 |
| **ITGB3BP** | 2.534 | **EIF4G2** | -3.785 | **LMO4** | 2.36 | **AFAP1** | -3.709 |
| **BOK** | 2.277 | **NEK6** | -3.76 | **TIMP2** | 2.349 | **DDX58** | -3.697 |
| **IL17RD** | 2.252 | **BIRC2** | -3.744 | **NUAK1** | 2.346 | **ITGA6** | -3.665 |
| **MYH9** | 2.208 | **IL6ST** | -3.727 | **LRPAP1** | 2.336 | **CCNB1** | -3.663 |
| **ACIN1** | 2.203 | **CXCL8** | -3.59 | **NOLC1** | 2.264 | **TGFBR3** | -3.607 |
| **DNAJA3** | 2.179 | **NEK7** | -3.481 | **SIP1** | 2.233 | **TFRC** | -3.539 |
| **EMP1** | 2.173 | **PPP3CB** | -3.478 | **CREBBP** | 2.207 | **IER3** | -3.343 |
| **CSF2RA** | 2.166 | **ADAR** | -3.472 | **DMC1** | 2.184 | **IFIH1** | -3.322 |
| **DPM3** | 2.15 | **TRIB1** | -3.286 | **DAG1** | 2.155 | **PNPT1** | -3.293 |
| **ANTXR1** | 2.124 | **AKAP12** | -3.279 | **DEPDC6** | 2.125 | **OLR1** | -3.236 |
| **SREBF1** | 2.099 | **AGO2** | -3.198 | **ACO2** | 2.101 | **TFPI** | -3.143 |
| **ETS1** | 2.086 | **NSF** | -3.186 | **EWSR1** | 2.075 | **GULP1** | -3.08 |
| **PPARBP** | 2.069 | **CYR61** | -3.158 | **RPS24** | 2.067 | **CHEK2** | -2.983 |
| **SET** | 2.028 | **CCT6A** | -3.12 | **TBP** | 2.041 | **F2R** | -2.95 |
| **BRMS1** | 2.019 | **PLAU** | -3.072 | **KRT8** | 2.034 | **SLC25A24** | -2.885 |
| **NFKBIB** | 2.012 | **MCL1** | -3.012 | **NME2** | 2.011 | **F2RL1** | -2.884 |
| **PTRH2** | 2.006 | **EGR2** | -3.01 | **RARA** | 2.005 | **GFPT1** | -2.835 |
| **IL10** | 1.979 | **LDLR** | -2.978 | **BCL2L12** | 2.002 | **SCP2** | -2.828 |
| **ISG15** | 1.962 | **DICER1** | -2.978 | **YWHAG** | 1.998 | **PPP2R5A** | -2.777 |
| **NUPR1** | 1.962 | **SERPINE1** | -2.973 | **PLEC1** | 1.99 | **ATG5** | -2.746 |
| **GADD45A** | 1.958 | **WEE1** | -2.951 | **TAOK3** | 1.972 | **ZAK** | -2.735 |
| **CCDC86** | 1.944 | **TBL1XR1** | -2.941 | **INTS1** | 1.966 | **ATG4A** | -2.686 |
| **CDK6** | 1.935 | **CTNNB1** | -2.941 | **MKL1** | 1.964 | **DOCK1** | -2.674 |
| **DAXX** | 1.89 | **VCL** | -2.927 | **CREB1** | 1.947 | **GCH1** | -2.667 |
| **MPRIP** | 1.865 | **ERCC1** | -2.888 | **TPD52** | 1.936 | **CASP7** | -2.665 |
| **PRELID1** | 1.864 | **ABCC5** | -2.869 | **SIGIRR** | 1.929 | **ZFYVE16** | -2.644 |
| **DIABLO** | 1.82 | **CD44** | -2.866 | **NUP62** | 1.926 | **ATP2A2** | -2.641 |
| **ID3** | 1.814 | **MTM1** | -2.838 | **HSPA1B** | 1.919 | **ZMYND11** | -2.627 |
| **E2F2** | 1.782 | **CDCP1** | -2.796 | **S100A4** | 1.906 | **FRMD6** | -2.595 |
| **TGFBI** | 1.774 | **APH1B** | -2.791 | **CAMK2G** | 1.902 | **CTNNA1** | -2.582 |
| **BMF** | 1.763 | **PRKCA** | -2.726 | **CBS** | 1.897 | **CTSL1** | -2.581 |
| **LAMP1** | 1.76 | **PROS1** | -2.711 | **ATF5** | 1.867 | **DRAM1** | -2.533 |
| **MBD4** | 1.758 | **PTHLH** | -2.695 | **E2F4** | 1.863 | **JAK1** | -2.514 |
| **IL18** | 1.758 | **NBN** | -2.657 | **DNAJB6** | 1.859 | **CCNC** | -2.508 |
| **RASSF6** | 1.755 | **BBS2** | -2.643 | **TRIAP1** | 1.858 | **DDX5** | -2.487 |
| **FAU** | 1.747 | **CITED2** | -2.617 | **TFF3** | 1.851 | **ITGB1** | -2.481 |
| **ADRM1** | 1.734 | **HSPA4** | -2.6 | **EBAG9** | 1.847 | **ULK2** | -2.479 |
| **MRPL41** | 1.733 | **TXNDC5** | -2.591 | **UBE2C** | 1.839 | **SPTBN1** | -2.478 |
| **UBE2L3** | 1.725 | **TGFBR2** | -2.574 | **LEP** | 1.833 | **ICAM1** | -2.449 |
| **SSTR2** | 1.72 | **RRAS** | -2.572 | **HOXC6** | 1.821 | **PDCD6IP** | -2.435 |
| **GPS2** | 1.703 | **PLK2** | -2.565 | **NCOA6** | 1.814 | **WASPIP** | -2.423 |
| **TNFAIP8L1** | 1.667 | **KIF14** | -2.559 | **CDR2L** | 1.812 | **RECK** | -2.393 |
| **NRGN** | 1.663 | **SGK1** | -2.554 | **MAD2L2** | 1.799 | **WTAP** | -2.382 |
| **BNIPL** | 1.653 | **FANCL** | -2.533 | **DDIT4** | 1.791 | **RHOB** | -2.37 |
| **CAMLG** | 1.651 | **MUC1** | -2.466 | **NDEL1** | 1.764 | **MX1** | -2.358 |
| **HRASLS** | 1.646 | **ITGAV** | -2.466 | **UXT** | 1.762 | **LGALS8** | -2.322 |
| **LGALS1** | 1.644 | **STAT5B** | -2.464 | **NUDT1** | 1.759 | **PRKDC** | -2.306 |
| **AKAP1** | 1.637 | **APIP** | -2.458 | **HSPBP1** | 1.751 | **PDCD2** | -2.3 |
| **PPP1R15A** | 1.632 | **SPRY2** | -2.451 | **TUB** | 1.73 | **CTH** | -2.295 |
| **EGLN2** | 1.628 | **NCEH1** | -2.44 | **MCM10** | 1.718 | **KRAS** | -2.288 |
| **CCAR1** | 1.618 | **NFKB1** | -2.435 | **CENPB** | 1.703 | **RASA1** | -2.286 |
| **DEDD2** | 1.611 | **PIK3CA** | -2.434 | **LSMD1** | 1.7 | **DNM1L** | -2.261 |
| **SIVA1** | 1.605 | **MFN1** | -2.409 | **SRC** | 1.692 | **HS.551128** | -2.255 |
| **STX1A** | 1.6 | **MAP3K7** | -2.4 | **NUF2** | 1.692 | **PHLDA1** | -2.25 |
| **ATN1** | 1.594 | **LIN7C** | -2.395 | **FBLN1** | 1.69 | **XPR1** | -2.241 |
| **CDC20** | 1.59 | **VAMP3** | -2.388 | **WDR4** | 1.685 | **MAP4K4** | -2.187 |
| **EMP3** | 1.587 | **HSD17B4** | -2.386 | **TNRC6A** | 1.684 | **TOP2A** | -2.183 |
| **CACNB3** | 1.582 | **SORL1** | -2.379 | **SCG5** | 1.681 | **PARP14** | -2.165 |
| **CDT1** | 1.582 | **UBA3** | -2.374 | **ERF** | 1.673 | **DSG2** | -2.162 |
| **SHC1** | 1.578 | **HIF1A** | -2.374 | **FBXO2** | 1.671 | **MAPK13** | -2.149 |
| **PDCD4** | 1.573 | **TOP2B** | -2.371 | **ARHGEF1** | 1.658 | **YES1** | -2.148 |
| **HIST1H1C** | 1.57 | **TP53BP1** | -2.37 | **FEN1** | 1.635 | **RNASEL** | -2.147 |
| **PTGDS** | 1.568 | **PLS1** | -2.369 | **GJC1** | 1.634 | **GPR37** | -2.137 |
| **CDK4** | 1.568 | **ANXA7** | -2.367 | **STXBP1** | 1.632 | **AP1G1** | -2.128 |
| **S100A6** | 1.563 | **NFYA** | -2.367 | **ABCC3** | 1.625 | **RFK** | -2.125 |
| **PPIB** | 1.563 | **LRP8** | -2.361 | **CHMP4B** | 1.625 | **RBBP4** | -2.111 |
| **TNFSF14** | 1.553 | **EOMES** | -2.36 | **PTP4A2** | 1.624 | **QKI** | -2.096 |
| **BRCA1** | 1.549 | **PPP2R1B** | -2.359 | **MDK** | 1.619 | **ROCK2** | -2.096 |
| **LILRB1** | 1.548 | **YWHAZ** | -2.351 | **CCBE1** | 1.617 | **SERINC3** | -2.087 |
| **LRDD** | 1.546 | **HMGCR** | -2.348 | **DNAJB2** | 1.616 | **TNFRSF10B** | -2.08 |
| **BNIP3L** | 1.545 | **ABCE1** | -2.342 | **TAF10** | 1.614 | **SEC23B** | -2.071 |
| **CYBA** | 1.544 | **CYP2J2** | -2.339 | **DAD1** | 1.602 | **AIFM1** | -2.053 |
| **MIR130A** | 1.539 | **PHIP** | -2.336 | **SP1** | 1.601 | **BPTF** | -2.043 |
| **DFFA** | 1.538 | **RBL2** | -2.325 | **EEF1D** | 1.591 | **SH3RF1** | -2.042 |
| **TNFRSF25** | 1.529 | **CD2AP** | -2.323 | **ARHGEF2** | 1.588 | **PARG** | -2.039 |
| **PIN1** | 1.519 | **FBXL5** | -2.308 | **APRT** | 1.582 | **TFPI2** | -2.025 |
| **FAM162A** | 1.519 | **KIFAP3** | -2.307 | **FANCD2** | 1.58 | **DDX3X** | -2.022 |
| **NME4** | 1.512 | **CAT** | -2.286 | **PRDX5** | 1.58 | **PKN2** | -2.021 |
| **ORAI1** | 1.508 | **EPCAM** | -2.282 | **MXD3** | 1.577 | **GNA12** | -2.02 |
| **PRPS1** | 1.507 | **MBTPS1** | -2.282 | **PTMA** | 1.576 | **SOAT1** | -2.018 |
| **MUTYH** | 1.506 | **TBK1** | -2.28 | **UBQLN1** | 1.576 | **PSEN2** | -2.009 |
| **KIAA0831** | 1.505 | **VPS13A** | -2.28 | **HSF1** | 1.576 | **RFWD2** | -2.005 |
|  |  | **NFE2L2** | -2.278 | **ARID1A** | 1.575 | **CASP3** | -1.996 |
|  |  | **CPEB4** | -2.26 | **PCK2** | 1.55 | **WDR19** | -1.995 |
|  |  | **PRKAA1** | -2.258 | **NRG2** | 1.538 | **PRKAR1A** | -1.993 |
|  |  | **PEX11B** | -2.256 | **CNTFR** | 1.537 | **IL1R1** | -1.993 |
|  |  | **TXNRD1** | -2.255 | **FANCE** | 1.536 | **ING3** | -1.992 |
|  |  | **LGR4** | -2.254 | **GADD45GIP1** | 1.527 | **CCDC109A** | -1.978 |
|  |  | **PDE4D** | -2.248 | **TNPO2** | 1.526 | **STAT4** | -1.968 |
|  |  | **STK40** | -2.243 | **MFGE8** | 1.512 | **ZMAT3** | -1.967 |
|  |  | **BCL2L13** | -2.23 | **NEDD8** | 1.51 | **SP110** | -1.952 |
|  |  | **SLC20A1** | -2.213 | **SRF** | 1.503 | **GSR** | -1.919 |
|  |  | **SERP1** | -2.21 |  |  | **ADAMTS1** | -1.918 |
|  |  | **BTRC** | -2.198 |  |  | **GMFB** | -1.917 |
|  |  | **CAPRIN1** | -2.198 |  |  | **TRIB3** | -1.914 |
|  |  | **ATP2B4** | -2.195 |  |  | **SMAD4** | -1.903 |
|  |  | **SIAH2** | -2.195 |  |  | **NAE1** | -1.898 |
|  |  | **RB1** | -2.193 |  |  | **PPP2CA** | -1.896 |
|  |  | **CERS6** | -2.187 |  |  | **ALDH1A3** | -1.89 |
|  |  | **IPPK** | -2.185 |  |  | **MCOLN3** | -1.888 |
|  |  | **FIGNL1** | -2.183 |  |  | **TLR1** | -1.876 |
|  |  | **RND3** | -2.173 |  |  | **CSE1L** | -1.869 |
|  |  | **EPAS1** | -2.164 |  |  | **MSH2** | -1.867 |
|  |  | **USP47** | -2.16 |  |  | **HS.531457** | -1.866 |
|  |  | **NAMPT** | -2.151 |  |  | **PAK2** | -1.864 |
|  |  | **USP18** | -2.15 |  |  | **MALT1** | -1.853 |
|  |  | **TPM3** | -2.15 |  |  | **PTPN1** | -1.85 |
|  |  | **TPR** | -2.15 |  |  | **TP53INP1** | -1.847 |
|  |  | **GRB10** | -2.147 |  |  | **ZFAND5** | -1.846 |
|  |  | **PIK3CB** | -2.146 |  |  | **APP** | -1.846 |
|  |  | **CAST** | -2.143 |  |  | **SNAP25** | -1.843 |
|  |  | **TMED10** | -2.141 |  |  | **OAS1** | -1.841 |
|  |  | **IFNL2** | -2.139 |  |  | **MLKL** | -1.84 |
|  |  | **TRIM24** | -2.135 |  |  | **KIF1B** | -1.837 |
|  |  | **SUN1** | -2.135 |  |  | **CREBL2** | -1.837 |
|  |  | **FAT4** | -2.128 |  |  | **IRF7** | -1.831 |
|  |  | **BRE** | -2.127 |  |  | **LCN2** | -1.83 |
|  |  | **MTMR6** | -2.126 |  |  | **CCDC88A** | -1.827 |
|  |  | **JUNB** | -2.12 |  |  | **CAPRIN2** | -1.826 |
|  |  | **ILK** | -2.118 |  |  | **LDHA** | -1.823 |
|  |  | **IREB2** | -2.113 |  |  | **FAF1** | -1.82 |
|  |  | **CD46** | -2.106 |  |  | **PTPN13** | -1.816 |
|  |  | **GEM** | -2.102 |  |  | **EIF2AK2** | -1.814 |
|  |  | **PDE4B** | -2.098 |  |  | **CTSB** | -1.812 |
|  |  | **CALM1 (includes others)** | -2.093 |  |  | **TRMT11** | -1.812 |
|  |  | **XRCC5** | -2.091 |  |  | **HTRA1** | -1.806 |
|  |  | **NFIB** | -2.09 |  |  | **FUT4** | -1.782 |
|  |  | **HSPD1** | -2.089 |  |  | **TIA1** | -1.781 |
|  |  | **KIF11** | -2.088 |  |  | **UACA** | -1.768 |
|  |  | **SLC2A3** | -2.08 |  |  | **IDE** | -1.76 |
|  |  | **TNFAIP8** | -2.074 |  |  | **HOXA5** | -1.757 |
|  |  | **HLTF** | -2.069 |  |  | **TTC5** | -1.748 |
|  |  | **ZMYM2** | -2.063 |  |  | **CYLD** | -1.742 |
|  |  | **SPINT1** | -2.061 |  |  | **SGPP1** | -1.742 |
|  |  | **RGPD4 (includes others)** | -2.061 |  |  | **TRPS1** | -1.736 |
|  |  | **RAB28** | -2.06 |  |  | **STAM2** | -1.734 |
|  |  | **MSRB3** | -2.059 |  |  | **TSC22D2** | -1.734 |
|  |  | **ANGPTL4** | -2.057 |  |  | **RHOH** | -1.729 |
|  |  | **NR2C1** | -2.055 |  |  | **MSI2** | -1.729 |
|  |  | **DHX9** | -2.054 |  |  | **MAP2K5** | -1.728 |
|  |  | **PIGA** | -2.048 |  |  | **HS.4988** | -1.725 |
|  |  | **BTG2** | -2.047 |  |  | **HMOX2** | -1.723 |
|  |  | **SORBS2** | -2.047 |  |  | **GNAQ** | -1.722 |
|  |  | **MXD1** | -2.047 |  |  | **STAP2** | -1.71 |
|  |  | **BHLHE40** | -2.044 |  |  | **TOP1** | -1.702 |
|  |  | **RPA1** | -2.034 |  |  | **ZMYM3** | -1.702 |
|  |  | **DPYD** | -2.033 |  |  | **SGPL1** | -1.701 |
|  |  | **HIPK3** | -2.029 |  |  | **PRKRA** | -1.699 |
|  |  | **MTMR2** | -2.026 |  |  | **AKR1C3** | -1.698 |
|  |  | **ITGA2** | -2.025 |  |  | **BACH2** | -1.697 |
|  |  | **SRI** | -2.024 |  |  | **USP12** | -1.697 |
|  |  | **CXCL1** | -2.023 |  |  | **CDC2** | -1.693 |
|  |  | **ASNS** | -2.018 |  |  | **LTB** | -1.691 |
|  |  | **UBR2** | -2.017 |  |  | **ITPR1** | -1.689 |
|  |  | **CUL2** | -2.005 |  |  | **PSMD6** | -1.687 |
|  |  | **DIDO1** | -2.004 |  |  | **ATG7** | -1.687 |
|  |  | **REL** | -1.995 |  |  | **ERCC3** | -1.682 |
|  |  | **SOX21** | -1.989 |  |  | **NSMAF** | -1.68 |
|  |  | **GLO1** | -1.987 |  |  | **FKBP1A** | -1.676 |
|  |  | **ROR1** | -1.987 |  |  | **KLRF1** | -1.676 |
|  |  | **HNRNPK** | -1.978 |  |  | **KIAA1468** | -1.676 |
|  |  | **ADH5** | -1.967 |  |  | **PPM1A** | -1.669 |
|  |  | **NFKBIA** | -1.955 |  |  | **TNF** | -1.663 |
|  |  | **ATP7A** | -1.948 |  |  | **TIRAP** | -1.662 |
|  |  | **SERPINI1** | -1.945 |  |  | **NUMB** | -1.66 |
|  |  | **ADAM17** | -1.945 |  |  | **RIPK1** | -1.655 |
|  |  | **TAX1BP1** | -1.94 |  |  | **ADRB1** | -1.654 |
|  |  | **OPTN** | -1.936 |  |  | **CASP8** | -1.652 |
|  |  | **ARF4** | -1.928 |  |  | **NOTCH2** | -1.649 |
|  |  | **VEGFC** | -1.925 |  |  | **RAB1A** | -1.648 |
|  |  | **OPA1** | -1.923 |  |  | **CKAP2** | -1.645 |
|  |  | **ILF3** | -1.919 |  |  | **TP53BP2** | -1.644 |
|  |  | **CD47** | -1.916 |  |  | **APPL1** | -1.637 |
|  |  | **CENPF** | -1.915 |  |  | **CDK2** | -1.635 |
|  |  | **SRXN1** | -1.911 |  |  | **VGF** | -1.633 |
|  |  | **ATP2B1** | -1.909 |  |  | **SPIN1** | -1.633 |
|  |  | **NFAT5** | -1.904 |  |  | **HIP2** | -1.631 |
|  |  | **MAPK1** | -1.901 |  |  | **EPHB2** | -1.628 |
|  |  | **PAFAH2** | -1.901 |  |  | **CDK5** | -1.627 |
|  |  | **CES1** | -1.891 |  |  | **WWC3** | -1.623 |
|  |  | **SNX7** | -1.883 |  |  | **CCNE1** | -1.619 |
|  |  | **SMARCA1** | -1.882 |  |  | **TMEM158** | -1.616 |
|  |  | **TEAD2** | -1.88 |  |  | **IL11RA** | -1.611 |
|  |  | **SMARCA5** | -1.879 |  |  | **MAP3K8** | -1.61 |
|  |  | **MAP3K2** | -1.87 |  |  | **BAX** | -1.608 |
|  |  | **RCAN1** | -1.858 |  |  | **SENP2** | -1.606 |
|  |  | **KPNA2** | -1.855 |  |  | **ANKRD1** | -1.602 |
|  |  | **BMI1** | -1.85 |  |  | **MYD88** | -1.602 |
|  |  | **MERTK** | -1.842 |  |  | **MAPK9** | -1.601 |
|  |  | **EXOC2** | -1.841 |  |  | **NIF3L1** | -1.597 |
|  |  | **MET** | -1.834 |  |  | **M6PR** | -1.596 |
|  |  | **DYNC1H1** | -1.833 |  |  | **ECT2** | -1.596 |
|  |  | **WAPAL** | -1.832 |  |  | **IFNAR1** | -1.595 |
|  |  | **API5** | -1.825 |  |  | **BID** | -1.594 |
|  |  | **AHR** | -1.82 |  |  | **FBXL2** | -1.594 |
|  |  | **RANBP2** | -1.818 |  |  | **HUWE1** | -1.594 |
|  |  | **EFEMP1** | -1.814 |  |  | **DEDD** | -1.592 |
|  |  | **NR2C2** | -1.813 |  |  | **TBCCD1** | -1.592 |
|  |  | **HEXB** | -1.808 |  |  | **ZFP36** | -1.591 |
|  |  | **STAT3** | -1.806 |  |  | **RANBP9** | -1.589 |
|  |  | **ANXA2** | -1.791 |  |  | **SORT1** | -1.588 |
|  |  | **NPC1** | -1.791 |  |  | **MAP3K9** | -1.587 |
|  |  | **C8orf44-SGK3/SGK3** | -1.79 |  |  | **STEAP3** | -1.586 |
|  |  | **GPHN** | -1.79 |  |  | **MOAP1** | -1.586 |
|  |  | **SON** | -1.786 |  |  | **PTPRA** | -1.583 |
|  |  | **YME1L1** | -1.784 |  |  | **ATP1A1** | -1.583 |
|  |  | **HDAC9** | -1.783 |  |  | **LATS1** | -1.58 |
|  |  | **DSP** | -1.776 |  |  | **ATF6** | -1.579 |
|  |  | **ALCAM** | -1.776 |  |  | **HAS3** | -1.579 |
|  |  | **HADHA** | -1.772 |  |  | **LATS2** | -1.579 |
|  |  | **SQSTM1** | -1.771 |  |  | **MEN1** | -1.579 |
|  |  | **PDPK1** | -1.769 |  |  | **PANX1** | -1.578 |
|  |  | **PFKM** | -1.761 |  |  | **MBP** | -1.575 |
|  |  | **CTSV** | -1.756 |  |  | **MBD1** | -1.574 |
|  |  | **LIMS1** | -1.755 |  |  | **PMEPA1** | -1.574 |
|  |  | **AURKA** | -1.755 |  |  | **CCNT1** | -1.573 |
|  |  | **ERCC5** | -1.755 |  |  | **MYO7A** | -1.573 |
|  |  | **MYO6** | -1.751 |  |  | **TFDP2** | -1.571 |
|  |  | **LGMN** | -1.748 |  |  | **CD40** | -1.568 |
|  |  | **XPO1** | -1.741 |  |  | **HOXA13** | -1.567 |
|  |  | **ERBB2** | -1.74 |  |  | **BMP6** | -1.566 |
|  |  | **LEPR** | -1.739 |  |  | **CCNL2** | -1.566 |
|  |  | **SLC12A2** | -1.739 |  |  | **SUB1** | -1.566 |
|  |  | **PPIA** | -1.736 |  |  | **STK3** | -1.563 |
|  |  | **ALDOA** | -1.736 |  |  | **TRIB2** | -1.563 |
|  |  | **YWHAB** | -1.733 |  |  | **SCRIB** | -1.562 |
|  |  | **mir-21** | -1.733 |  |  | **PLSCR1** | -1.562 |
|  |  | **ADM** | -1.732 |  |  | **RPS6KA2** | -1.56 |
|  |  | **ANXA5** | -1.73 |  |  | **STK17A** | -1.558 |
|  |  | **IGF1R** | -1.729 |  |  | **CSNK2A1** | -1.557 |
|  |  | **POT1** | -1.727 |  |  | **MAP1B** | -1.557 |
|  |  | **FAIM** | -1.72 |  |  | **RALB** | -1.556 |
|  |  | **TCERG1** | -1.719 |  |  | **TCF12** | -1.551 |
|  |  | **BAZ1A** | -1.716 |  |  | **TNFRSF12A** | -1.55 |
|  |  | **NRAS** | -1.713 |  |  | **RNPS1** | -1.55 |
|  |  | **CKAP5** | -1.712 |  |  | **E2F3** | -1.549 |
|  |  | **CDK8** | -1.71 |  |  | **CCBL1** | -1.549 |
|  |  | **CHUK** | -1.708 |  |  | **CAPN2** | -1.548 |
|  |  | **CTTN** | -1.707 |  |  | **DAB2** | -1.548 |
|  |  | **FAM134B** | -1.706 |  |  | **TRIM13** | -1.548 |
|  |  | **MCRS1** | -1.705 |  |  | **CASP6** | -1.545 |
|  |  | **CAV1** | -1.702 |  |  | **RASL10A** | -1.543 |
|  |  | **MTMR9** | -1.7 |  |  | **PERP** | -1.542 |
|  |  | **AMACR** | -1.699 |  |  | **PTGER4** | -1.538 |
|  |  | **YAP1** | -1.696 |  |  | **ACER2** | -1.536 |
|  |  | **DVL1** | -1.693 |  |  | **CUL4B** | -1.533 |
|  |  | **PTPN11** | -1.688 |  |  | **TAF1B** | -1.533 |
|  |  | **RECQL** | -1.687 |  |  | **ARL8B** | -1.532 |
|  |  | **FDFT1** | -1.687 |  |  | **OAS3** | -1.528 |
|  |  | **MSH3** | -1.686 |  |  | **ARRB1** | -1.526 |
|  |  | **HGF** | -1.685 |  |  | **ZNF148** | -1.526 |
|  |  | **NFKB2** | -1.685 |  |  | **RAC1** | -1.523 |
|  |  | **HSP90B1** | -1.683 |  |  | **KLF13** | -1.52 |
|  |  | **BIRC6** | -1.682 |  |  | **APOBEC3B** | -1.52 |
|  |  | **GCLM** | -1.681 |  |  | **NDUFAF1** | -1.52 |
|  |  | **VCP** | -1.681 |  |  | **ATP6AP2** | -1.516 |
|  |  | **SPG7** | -1.679 |  |  | **CUTL1** | -1.51 |
|  |  | **PALLD** | -1.676 |  |  | **STK24** | -1.509 |
|  |  | **PLD1** | -1.675 |  |  | **OMA1** | -1.508 |
|  |  | **RELB** | -1.674 |  |  | **EEF1A1** | -1.506 |
|  |  | **CD164** | -1.673 |  |  | **MIB1** | -1.506 |
|  |  | **CUL1** | -1.673 |  |  | **WDR48** | -1.506 |
|  |  | **PICK1** | -1.672 |  |  | **IFNGR1** | -1.504 |
|  |  | **MDM2** | -1.672 |  |  | **MIR302C** | -1.503 |
|  |  | **SLC9A4** | -1.672 |  |  | **PRMT2** | -1.502 |
|  |  | **ADNP2** | -1.671 |  |  | **TNFRSF10A** | -1.502 |
|  |  | **NCOA2** | -1.666 |  |  | **ATP7B** | -1.502 |
|  |  | **KIF3A** | -1.666 |  |  | **TJP2** | -1.501 |
|  |  | **ABCB7** | -1.665 |  |  |  |  |
|  |  | **RGN** | -1.664 |  |  |  |  |
|  |  | **TASP1** | -1.664 |  |  |  |  |
|  |  | **ABCC1** | -1.655 |  |  |  |  |
|  |  | **CENPE** | -1.652 |  |  |  |  |
|  |  | **VPS41** | -1.652 |  |  |  |  |
|  |  | **RABGGTA** | -1.652 |  |  |  |  |
|  |  | **ANXA4** | -1.649 |  |  |  |  |
|  |  | **AFG3L2** | -1.649 |  |  |  |  |
|  |  | **YWHAE** | -1.645 |  |  |  |  |
|  |  | **SP3** | -1.643 |  |  |  |  |
|  |  | **ALMS1** | -1.643 |  |  |  |  |
|  |  | **MAP2K1** | -1.639 |  |  |  |  |
|  |  | **HEYL** | -1.637 |  |  |  |  |
|  |  | **CYP26B1** | -1.636 |  |  |  |  |
|  |  | **CD151** | -1.634 |  |  |  |  |
|  |  | **PKM** | -1.63 |  |  |  |  |
|  |  | **PARP16** | -1.63 |  |  |  |  |
|  |  | **NCAPG2** | -1.628 |  |  |  |  |
|  |  | **SLCO1B3** | -1.622 |  |  |  |  |
|  |  | **TAF9B** | -1.622 |  |  |  |  |
|  |  | **PIK3R1** | -1.619 |  |  |  |  |
|  |  | **RAD50** | -1.617 |  |  |  |  |
|  |  | **TMX1** | -1.617 |  |  |  |  |
|  |  | **FGF2** | -1.613 |  |  |  |  |
|  |  | **VAMP2** | -1.613 |  |  |  |  |
|  |  | **IFIT3** | -1.613 |  |  |  |  |
|  |  | **GORASP1** | -1.611 |  |  |  |  |
|  |  | **RNF34** | -1.609 |  |  |  |  |
|  |  | **CCND1** | -1.609 |  |  |  |  |
|  |  | **AGO4** | -1.606 |  |  |  |  |
|  |  | **MAP3K4** | -1.604 |  |  |  |  |
|  |  | **CCT7** | -1.603 |  |  |  |  |
|  |  | **CDC14A** | -1.602 |  |  |  |  |
|  |  | **TSG101** | -1.595 |  |  |  |  |
|  |  | **DDR1** | -1.595 |  |  |  |  |
|  |  | **PON2** | -1.593 |  |  |  |  |
|  |  | **RDH10** | -1.593 |  |  |  |  |
|  |  | **PIAS2** | -1.59 |  |  |  |  |
|  |  | **PSMB8** | -1.59 |  |  |  |  |
|  |  | **KLK3** | -1.59 |  |  |  |  |
|  |  | **PPP1R2** | -1.589 |  |  |  |  |
|  |  | **TRAF5** | -1.589 |  |  |  |  |
|  |  | **RAP1GAP** | -1.588 |  |  |  |  |
|  |  | **RAP1B** | -1.588 |  |  |  |  |
|  |  | **IKBKAP** | -1.587 |  |  |  |  |
|  |  | **CDH1** | -1.587 |  |  |  |  |
|  |  | **PRDX3** | -1.584 |  |  |  |  |
|  |  | **NUPL1** | -1.584 |  |  |  |  |
|  |  | **DPP8** | -1.583 |  |  |  |  |
|  |  | **SLX1A/SLX1B** | -1.582 |  |  |  |  |
|  |  | **SPP1** | -1.581 |  |  |  |  |
|  |  | **OGG1** | -1.58 |  |  |  |  |
|  |  | **CALCRL** | -1.578 |  |  |  |  |
|  |  | **TCP1** | -1.577 |  |  |  |  |
|  |  | **CERKL** | -1.575 |  |  |  |  |
|  |  | **SELE** | -1.574 |  |  |  |  |
|  |  | **AGGF1** | -1.574 |  |  |  |  |
|  |  | **CCT4** | -1.573 |  |  |  |  |
|  |  | **TANK** | -1.571 |  |  |  |  |
|  |  | **NEK2** | -1.57 |  |  |  |  |
|  |  | **FER** | -1.569 |  |  |  |  |
|  |  | **METAP2** | -1.567 |  |  |  |  |
|  |  | **NPHS1** | -1.566 |  |  |  |  |
|  |  | **STAM** | -1.566 |  |  |  |  |
|  |  | **ATP2C1** | -1.566 |  |  |  |  |
|  |  | **E2F6** | -1.565 |  |  |  |  |
|  |  | **KCNMA1** | -1.565 |  |  |  |  |
|  |  | **SETX** | -1.565 |  |  |  |  |
|  |  | **PDE9A** | -1.564 |  |  |  |  |
|  |  | **HK2** | -1.563 |  |  |  |  |
|  |  | **ABCC4** | -1.562 |  |  |  |  |
|  |  | **CLCN3** | -1.558 |  |  |  |  |
|  |  | **CHKA** | -1.557 |  |  |  |  |
|  |  | **FGFRL1** | -1.556 |  |  |  |  |
|  |  | **ACSL4** | -1.555 |  |  |  |  |
|  |  | **HSPA9** | -1.555 |  |  |  |  |
|  |  | **SMO** | -1.553 |  |  |  |  |
|  |  | **FASN** | -1.553 |  |  |  |  |
|  |  | **TMBIM4** | -1.551 |  |  |  |  |
|  |  | **USP10** | -1.55 |  |  |  |  |
|  |  | **LIG4** | -1.55 |  |  |  |  |
|  |  | **TRPC1** | -1.549 |  |  |  |  |
|  |  | **TEX11** | -1.549 |  |  |  |  |
|  |  | **ITGB4** | -1.547 |  |  |  |  |
|  |  | **PDGFC** | -1.546 |  |  |  |  |
|  |  | **ASAH1** | -1.545 |  |  |  |  |
|  |  | **AGO3** | -1.545 |  |  |  |  |
|  |  | **INTS3** | -1.541 |  |  |  |  |
|  |  | **B4GALNT1** | -1.54 |  |  |  |  |
|  |  | **PRKD3** | -1.54 |  |  |  |  |
|  |  | **HOXB4** | -1.538 |  |  |  |  |
|  |  | **ATP11C** | -1.538 |  |  |  |  |
|  |  | **EGFR** | -1.535 |  |  |  |  |
|  |  | **PARD3** | -1.535 |  |  |  |  |
|  |  | **PARK2** | -1.534 |  |  |  |  |
|  |  | **DUSP10** | -1.531 |  |  |  |  |
|  |  | **JAK2** | -1.53 |  |  |  |  |
|  |  | **UGCG** | -1.527 |  |  |  |  |
|  |  | **CXADR** | -1.527 |  |  |  |  |
|  |  | **KCND2** | -1.527 |  |  |  |  |
|  |  | **HSF2** | -1.526 |  |  |  |  |
|  |  | **NEK1** | -1.525 |  |  |  |  |
|  |  | **PPID** | -1.524 |  |  |  |  |
|  |  | **RBM3** | -1.524 |  |  |  |  |
|  |  | **PAF1** | -1.523 |  |  |  |  |
|  |  | **ALDH2** | -1.521 |  |  |  |  |
|  |  | **VAPA** | -1.519 |  |  |  |  |
|  |  | **PRKCE** | -1.518 |  |  |  |  |
|  |  | **ARSB** | -1.516 |  |  |  |  |
|  |  | **GLUD1** | -1.515 |  |  |  |  |
|  |  | **PRKD1** | -1.515 |  |  |  |  |
|  |  | **TIMP1** | -1.514 |  |  |  |  |
|  |  | **KDM2B** | -1.513 |  |  |  |  |
|  |  | **CTBP2** | -1.512 |  |  |  |  |
|  |  | **PSMC1** | -1.511 |  |  |  |  |
|  |  | **FBXW7** | -1.511 |  |  |  |  |
|  |  | **RAD18** | -1.51 |  |  |  |  |
|  |  | **LCMT1** | -1.508 |  |  |  |  |
|  |  | **TPP2** | -1.505 |  |  |  |  |
|  |  | **LYNX1** | -1.504 |  |  |  |  |
|  |  | **KRT18** | -1.503 |  |  |  |  |
|  |  | **EIF4E** | -1.502 |  |  |  |  |
|  |  | **TLR6** | -1.501 |  |  |  |  |

C.

| **Expected** | | | | **Inversely related** | | | |
| --- | --- | --- | --- | --- | --- | --- | --- |
| **decreased migration** | | | | **increased migration** | | | |
| **up-genes (12)** | fold change | **down genes (123)** | fold change | **up-genes (19)** | fold change | **down genes (27)** | fold change |
| **HMOX1** | 3.189 | **EGR1** | -13.207 | **C1QBP** | 3.093 | **STAT1** | -5.293 |
| **TIMP2** | 2.349 | **CTGF** | -5.348 | **PPFIA1** | 2.365 | **ARRDC3** | -3.84 |
| **MYH9** | 2.208 | **LIN28B** | -3.856 | **ARF1** | 2.207 | **LAMC2** | -3.579 |
| **DNAJA3** | 2.179 | **RPS6KA3** | -3.8 | **DPAGT1** | 2.185 | **SPRY2** | -2.451 |
| **KRT8** | 2.034 | **AFAP1** | -3.709 | **CDC25B** | 2.173 | **RASA1** | -2.286 |
| **BRMS1** | 2.019 | **DDX58** | -3.697 | **ACTN4** | 2.107 | **CAT** | -2.286 |
| **DNAJB6** | 1.859 | **ITGA6** | -3.665 | **ETS1** | 2.086 | **FAM188A** | -2.249 |
| **CMTM8** | 1.717 | **CXCL8** | -3.59 | **S100A4** | 1.906 | **TPM3** | -2.15 |
| **SP1** | 1.601 | **AKAP11** | -3.208 | **HNRNPA2B1** | 1.838 | **ASNS** | -2.018 |
| **PDCD4** | 1.573 | **CYR61** | -3.158 | **LEP** | 1.833 | **CD47** | -1.916 |
| **BRCA1** | 1.549 | **PLAU** | -3.072 | **MTA1** | 1.752 | **ADAM9** | -1.909 |
| **mir-130** | 1.539 | **SERPINE1** | -2.973 | **SRC** | 1.692 | **PTPN1** | -1.85 |
|  |  | **F2R** | -2.95 | **ARHGEF1** | 1.658 | **TP53INP1** | -1.847 |
|  |  | **CTNNB1** | -2.941 | **LGALS1** | 1.644 | **FAF1** | -1.82 |
|  |  | **F2RL1** | -2.884 | **PTP4A2** | 1.624 | **GNAI1** | -1.796 |
|  |  | **E2F5** | -2.873 | **MDK** | 1.619 | **SACM1L** | -1.725 |
|  |  | **CD44** | -2.866 | **SHC1** | 1.578 | **PALLD** | -1.676 |
|  |  | **CDCP1** | -2.796 | **MAPK8IP3** | 1.575 | **EBI3** | -1.623 |
|  |  | **PRKCA** | -2.726 | **ORAI1** | 1.508 | **FBXL2** | -1.594 |
|  |  | **PTHLH** | -2.695 |  |  | **WDR44** | -1.592 |
|  |  | **DOCK1** | -2.674 |  |  | **RAP1GAP** | -1.588 |
|  |  | **FNBP1L** | -2.614 |  |  | **CDH1** | -1.587 |
|  |  | **JAK1** | -2.514 |  |  | **MEN1** | -1.579 |
|  |  | **ITGB1** | -2.481 |  |  | **BMP6** | -1.566 |
|  |  | **MUC1** | -2.466 |  |  | **PARK2** | -1.534 |
|  |  | **ITGAV** | -2.466 |  |  | **PRKD1** | -1.515 |
|  |  | **MAP3K7** | -2.4 |  |  | **TIMP1** | -1.514 |
|  |  | **BMPR2** | -2.396 |  |  |  |  |
|  |  | **HIF1A** | -2.374 |  |  |  |  |
|  |  | **RHOB** | -2.37 |  |  |  |  |
|  |  | **CYP2J2** | -2.339 |  |  |  |  |
|  |  | **ARFGEF1** | -2.293 |  |  |  |  |
|  |  | **KRAS** | -2.288 |  |  |  |  |
|  |  | **NRG1** | -2.255 |  |  |  |  |
|  |  | **MAP4K4** | -2.187 |  |  |  |  |
|  |  | **ILK** | -2.118 |  |  |  |  |
|  |  | **NES** | -2.109 |  |  |  |  |
|  |  | **TNFAIP8** | -2.074 |  |  |  |  |
|  |  | **ITGA2** | -2.025 |  |  |  |  |
|  |  | **GNA12** | -2.02 |  |  |  |  |
|  |  | **ANKRD28** | -2.007 |  |  |  |  |
|  |  | **DIDO1** | -2.004 |  |  |  |  |
|  |  | **CUL4A** | -1.987 |  |  |  |  |
|  |  | **ROR1** | -1.987 |  |  |  |  |
|  |  | **HNRNPK** | -1.978 |  |  |  |  |
|  |  | **ADAM17** | -1.945 |  |  |  |  |
|  |  | **SDCBP** | -1.94 |  |  |  |  |
|  |  | **VEGFC** | -1.925 |  |  |  |  |
|  |  | **GNAI3** | -1.924 |  |  |  |  |
|  |  | **WWTR1** | -1.923 |  |  |  |  |
|  |  | **ILF3** | -1.919 |  |  |  |  |
|  |  | **USP9X** | -1.909 |  |  |  |  |
|  |  | **SMAD4** | -1.903 |  |  |  |  |
|  |  | **MAPK1** | -1.901 |  |  |  |  |
|  |  | **CSE1L** | -1.869 |  |  |  |  |
|  |  | **PAK2** | -1.864 |  |  |  |  |
|  |  | **KPNA2** | -1.855 |  |  |  |  |
|  |  | **BMI1** | -1.85 |  |  |  |  |
|  |  | **APP** | -1.846 |  |  |  |  |
|  |  | **MERTK** | -1.842 |  |  |  |  |
|  |  | **MET** | -1.834 |  |  |  |  |
|  |  | **CCDC88A** | -1.827 |  |  |  |  |
|  |  | **EIF2AK2** | -1.814 |  |  |  |  |
|  |  | **RUVBL1** | -1.811 |  |  |  |  |
|  |  | **LTBP2** | -1.807 |  |  |  |  |
|  |  | **STAT3** | -1.806 |  |  |  |  |
|  |  | **ANXA2** | -1.791 |  |  |  |  |
|  |  | **FOXQ1** | -1.778 |  |  |  |  |
|  |  | **ALCAM** | -1.776 |  |  |  |  |
|  |  | **PRMT6** | -1.769 |  |  |  |  |
|  |  | **AURKA** | -1.755 |  |  |  |  |
|  |  | **ERBB2** | -1.74 |  |  |  |  |
|  |  | **SLC12A2** | -1.739 |  |  |  |  |
|  |  | **mir-21** | -1.733 |  |  |  |  |
|  |  | **IGF1R** | -1.729 |  |  |  |  |
|  |  | **MGAT5** | -1.725 |  |  |  |  |
|  |  | **NRAS** | -1.713 |  |  |  |  |
|  |  | **CTTN** | -1.707 |  |  |  |  |
|  |  | **LIMK1** | -1.704 |  |  |  |  |
|  |  | **CAV1** | -1.702 |  |  |  |  |
|  |  | **ITPR1** | -1.689 |  |  |  |  |
|  |  | **PTPN11** | -1.688 |  |  |  |  |
|  |  | **HGF** | -1.685 |  |  |  |  |
|  |  | **VCP** | -1.681 |  |  |  |  |
|  |  | **PLD1** | -1.675 |  |  |  |  |
|  |  | **TNF** | -1.663 |  |  |  |  |
|  |  | **CASP8** | -1.652 |  |  |  |  |
|  |  | **NOTCH2** | -1.649 |  |  |  |  |
|  |  | **PODXL** | -1.639 |  |  |  |  |
|  |  | **MAP2K1** | -1.639 |  |  |  |  |
|  |  | **CD151** | -1.634 |  |  |  |  |
|  |  | **VGF** | -1.633 |  |  |  |  |
|  |  | **EPHB2** | -1.628 |  |  |  |  |
|  |  | **CDK5** | -1.627 |  |  |  |  |
|  |  | **FGF2** | -1.613 |  |  |  |  |
|  |  | **MAP3K8** | -1.61 |  |  |  |  |
|  |  | **SYNM** | -1.604 |  |  |  |  |
|  |  | **ECT2** | -1.596 |  |  |  |  |
|  |  | **IFNAR1** | -1.595 |  |  |  |  |
|  |  | **RAB21** | -1.592 |  |  |  |  |
|  |  | **RANBP9** | -1.589 |  |  |  |  |
|  |  | **RAP1B** | -1.588 |  |  |  |  |
|  |  | **SPP1** | -1.581 |  |  |  |  |
|  |  | **HAS3** | -1.579 |  |  |  |  |
|  |  | **FER** | -1.569 |  |  |  |  |
|  |  | **CD40** | -1.568 |  |  |  |  |
|  |  | **RALB** | -1.556 |  |  |  |  |
|  |  | **ACSL4** | -1.555 |  |  |  |  |
|  |  | **TCF12** | -1.551 |  |  |  |  |
|  |  | **TMBIM4** | -1.551 |  |  |  |  |
|  |  | **WASL** | -1.55 |  |  |  |  |
|  |  | **CAPN2** | -1.548 |  |  |  |  |
|  |  | **IQGAP1** | -1.548 |  |  |  |  |
|  |  | **DAB2** | -1.548 |  |  |  |  |
|  |  | **PTGER4** | -1.538 |  |  |  |  |
|  |  | **EGFR** | -1.535 |  |  |  |  |
|  |  | **JAK2** | -1.53 |  |  |  |  |
|  |  | **GOLPH3** | -1.529 |  |  |  |  |
|  |  | **ARRB1** | -1.526 |  |  |  |  |
|  |  | **RAC1** | -1.523 |  |  |  |  |
|  |  | **LCK** | -1.516 |  |  |  |  |
|  |  | **CTBP2** | -1.512 |  |  |  |  |
|  |  | **CTNNAL1** | -1.504 |  |  |  |  |
