# Supplementary material for: Specificity protein (Sp) transcription factors Sp1, Sp3 and Sp4 are non-oncogene addiction genes in cancer cells
Source: Oncotarget. 2016 Mar 5;7(16):22245–56. doi: 10.18632/oncotarget.7925 (PMC5008359; doi:10.18632/oncotarget.7925)
Supplement: Supplementary file 3 [file oncotarget-07-22245-s003.docx]

Supplemental Table S2. Sp3-regulated associated with growth inhibition, cell death and inhibition of migration/invasion after Sp3 knockdown: expected and inversely regulated genes.

A.

| **Expected** | | |  | **Inversely related** | | |  |
| --- | --- | --- | --- | --- | --- | --- | --- |
| **decreased cell proliferation** | | |  | **increased cell proliferation** | | |  |
| **up-genes (58)** | fold change | **down genes (429)** | fold change | **up-genes (112)** | fold change | **down genes (244)** | fold change |
| **TXNIP** | 5.257 | **EGR1** | -15.086 | **LMNA** | 12.109 | **TRIB1** | -4.97 |
| **SREBF1** | 4.275 | **RAC1** | -8.138 | **CBX7** | 4.092 | **CAST** | -4.411 |
| **CDCA4** | 3.527 | **RRM2** | -7.011 | **SET** | 3.643 | **RNF144** | -4.078 |
| **RNH1** | 3.391 | **IL8** | -6.204 | **C1QBP** | 3.473 | **STAT1** | -4.047 |
| **HMGB3** | 3.034 | **BIRC3** | -6.182 | **DEPDC6** | 3.277 | **TNFRSF21** | -4.036 |
| **UCP2** | 2.945 | **FOS** | -5.573 | **TFF3** | 3.153 | **STEAP3** | -3.66 |
| **SIP1** | 2.815 | **LAMC1** | -5.183 | **HMGB2** | 2.987 | **PHLDA1** | -3.657 |
| **PCBP4** | 2.509 | **CTGF** | -4.591 | **HMGB1** | 2.955 | **ARRDC3** | -3.535 |
| **FBXO2** | 2.378 | **PLAU** | -4.394 | **LRPAP1** | 2.864 | **EGR2** | -3.392 |
| **SIGIRR** | 2.242 | **DUSP6** | -4.233 | **SUMO2** | 2.73 | **YWHAZ** | -3.362 |
| **MED25** | 2.232 | **PRPS2** | -4.109 | **RCE1** | 2.715 | **WASPIP** | -3.325 |
| **DACT3** | 2.223 | **DDX58** | -4.085 | **PTPMT1** | 2.69 | **SPRY2** | -3.196 |
| **NUMA1** | 2.124 | **DKK1** | -4.018 | **HOXC6** | 2.663 | **RAD17** | -3.17 |
| **DNAJA3** | 2.113 | **SDCBP** | -3.874 | **H3F3B** | 2.633 | **FRMD6** | -3.158 |
| **MXD4** | 2.018 | **VCL** | -3.829 | **NCOR2** | 2.551 | **TGFBR2** | -3.154 |
| **EIF1** | 2.017 | **CYP2J2** | -3.777 | **PLAC8** | 2.516 | **KLK3** | -3.093 |
| **MSX1** | 1.973 | **ITGB1** | -3.766 | **EMP1** | 2.435 | **CD83** | -3.068 |
| **ACTN4** | 1.946 | **NFKBIA** | -3.735 | **DIXDC1** | 2.364 | **TOM1L1** | -2.981 |
| **HSPA1B** | 1.939 | **E2F5** | -3.714 | **YWHAG** | 2.3 | **AKAP12** | -2.889 |
| **CDKN2C** | 1.935 | **OPA1** | -3.536 | **S100A4** | 2.256 | **SPRY1** | -2.87 |
| **GPR56** | 1.925 | **CXCL1** | -3.471 | **PFDN5** | 2.173 | **ASPH** | -2.842 |
| **NR2F2** | 1.864 | **F2RL1** | -3.358 | **H2AFX** | 2.155 | **TFPI** | -2.787 |
| **LGALS7** | 1.803 | **NQO1** | -3.309 | **CORO1B** | 2.131 | **BMPR2** | -2.784 |
| **TSC22D3** | 1.778 | **DOCK1** | -3.257 | **SERPINH1** | 2.094 | **DUSP5** | -2.772 |
| **MUL1** | 1.731 | **RAF1** | -3.186 | **SRF** | 2.08 | **EIF4G2** | -2.741 |
| **COMMD5** | 1.722 | **NRG1** | -3.129 | **PIM3** | 2.073 | **RB1** | -2.707 |
| **CDKN2D** | 1.717 | **SEMA4D** | -3.083 | **EWSR1** | 2.041 | **SPRY4** | -2.691 |
| **HRASLS** | 1.714 | **VASP** | -3.034 | **CBS** | 2.022 | **PPP2R2C** | -2.691 |
| **AGR2** | 1.713 | **CTNNB1** | -3.028 | **ACLY** | 2.006 | **CASP2** | -2.648 |
| **RPL26** | 1.712 | **PPP1R13L** | -3.01 | **TBP** | 2.003 | **SMAD3** | -2.627 |
| **SH2B3** | 1.705 | **KIF2A** | -2.967 | **NDEL1** | 1.973 | **PNPT1** | -2.597 |
| **DAXX** | 1.703 | **CD151** | -2.924 | **ISG15** | 1.97 | **NFE2L2** | -2.573 |
| **MTA1** | 1.691 | **BIRC2** | -2.921 | **NOP58** | 1.961 | **ABCC5** | -2.562 |
| **CBL** | 1.683 | **CCL20** | -2.862 | **TPD52** | 1.951 | **EIF2AK2** | -2.549 |
| **STRA13** | 1.68 | **NASP** | -2.853 | **RPS15A** | 1.938 | **SERINC3** | -2.518 |
| **RALBP1** | 1.655 | **SERPINE1** | -2.835 | **NACA** | 1.927 | **CAV1** | -2.515 |
| **PEBP1** | 1.63 | **EOMES** | -2.815 | **POLR2L** | 1.917 | **CAPRIN2** | -2.477 |
| **CDKN1C** | 1.62 | **PLK2** | -2.805 | **NOLC1** | 1.909 | **RASA1** | -2.476 |
| **EI24** | 1.604 | **LCN2** | -2.759 | **ATP5G1** | 1.893 | **IFNGR1** | -2.47 |
| **ATPIF1** | 1.6 | **TXNDC5** | -2.702 | **ARF1** | 1.885 | **PIAS3** | -2.463 |
| **CD24** | 1.6 | **AFAP1** | -2.7 | **LSMD1** | 1.868 | **RNASEL** | -2.461 |
| **ERF** | 1.59 | **ERCC1** | -2.684 | **MCM8** | 1.851 | **TRIB3** | -2.368 |
| **NFKBIB** | 1.581 | **ADAR** | -2.639 | **DVL2** | 1.826 | **BHLHB2** | -2.357 |
| **ARID1A** | 1.575 | **NBN** | -2.614 | **CTSD** | 1.819 | **CBFB** | -2.357 |
| **EMD** | 1.558 | **MAPK9** | -2.609 | **LGALS1** | 1.802 | **HAS3** | -2.356 |
| **MNX1** | 1.554 | **YAP1** | -2.596 | **NAP1L1** | 1.802 | **BTG2** | -2.355 |
| **PKD1** | 1.553 | **PRKAA1** | -2.582 | **SPTAN1** | 1.793 | **TFPI2** | -2.296 |
| **DBI** | 1.551 | **STAM2** | -2.568 | **ETS1** | 1.787 | **ROCK1** | -2.293 |
| **LAMA5** | 1.54 | **MCL1** | -2.526 | **CREB1** | 1.782 | **DFNA5** | -2.283 |
| **NDUFS3** | 1.539 | **DNM1L** | -2.506 | **PIP5K2B** | 1.78 | **PMP22** | -2.272 |
| **GPI** | 1.538 | **IER3** | -2.506 | **CSF2RA** | 1.777 | **KDM3B** | -2.263 |
| **UXT** | 1.536 | **SSR1** | -2.498 | **VKORC1** | 1.777 | **APBB2** | -2.252 |
| **DNAJB6** | 1.534 | **CD81** | -2.482 | **RPS9** | 1.755 | **PPP2R1B** | -2.252 |
| **GADD45A** | 1.519 | **CDC16** | -2.468 | **CDC42** | 1.731 | **NOTCH2** | -2.247 |
| **C1QL4** | 1.509 | **PIGN** | -2.447 | **DPYSL2** | 1.725 | **TICAM2** | -2.242 |
| **GNE** | 1.508 | **SGK3** | -2.441 | **E2F4** | 1.721 | **CTH** | -2.236 |
| **MAFG** | 1.502 | **JAK1** | -2.43 | **DYRK1B** | 1.716 | **G3BP1** | -2.234 |
| **IFITM1** | 1.501 | **NOC3L** | -2.425 | **RANBP1** | 1.715 | **CRLF3** | -2.228 |
|  |  | **DICER1** | -2.424 | **PSMA4** | 1.711 | **NDFIP1** | -2.216 |
|  |  | **CCNB1** | -2.424 | **AHNAK** | 1.71 | **CASP7** | -2.216 |
|  |  | **CCDC6** | -2.416 | **CRCP** | 1.707 | **TAX1BP3** | -2.213 |
|  |  | **TNF** | -2.402 | **LEP** | 1.704 | **ITGA6** | -2.21 |
|  |  | **B4GALT6** | -2.395 | **HS.57079** | 1.694 | **ROCK2** | -2.194 |
|  |  | **ADCY3** | -2.394 | **S100A6** | 1.681 | **MERTK** | -2.188 |
|  |  | **CD44** | -2.382 | **HS.535028** | 1.67 | **UBE2D3** | -2.184 |
|  |  | **REL** | -2.38 | **BASP1** | 1.663 | **CAT** | -2.164 |
|  |  | **ITGB4** | -2.377 | **MKL1** | 1.657 | **WDR48** | -2.144 |
|  |  | **NR2C2** | -2.373 | **ILF3** | 1.652 | **SEC23A** | -2.123 |
|  |  | **MAP3K11** | -2.354 | **ODC1** | 1.652 | **ERRFI1** | -2.107 |
|  |  | **PDE4D** | -2.344 | **S100A13** | 1.649 | **LIFR** | -2.1 |
|  |  | **EIF2C2** | -2.342 | **HSPA5** | 1.648 | **PKP2** | -2.097 |
|  |  | **HS.128753** | -2.337 | **CYBA** | 1.645 | **RBL2** | -2.096 |
|  |  | **CCDC115** | -2.337 | **GPR177** | 1.644 | **RNF14** | -2.086 |
|  |  | **RAB22A** | -2.332 | **CCNF** | 1.642 | **QPCT** | -2.085 |
|  |  | **RAN** | -2.33 | **CTSB** | 1.64 | **TJP1** | -2.079 |
|  |  | **SDC4** | -2.323 | **IL11** | 1.634 | **RCHY1** | -2.075 |
|  |  | **ELMO2** | -2.316 | **DIDO1** | 1.63 | **RNF10** | -2.061 |
|  |  | **USP18** | -2.311 | **VEGFB** | 1.627 | **ANXA7** | -2.048 |
|  |  | **CTTN** | -2.309 | **PPARBP** | 1.613 | **JUNB** | -2.031 |
|  |  | **ATP7A** | -2.299 | **ICMT** | 1.609 | **KIFAP3** | -2.015 |
|  |  | **CHUK** | -2.299 | **ISG20** | 1.6 | **KRIT1** | -2.004 |
|  |  | **RNMT** | -2.288 | **CDC25B** | 1.597 | **WTAP** | -1.972 |
|  |  | **TRPC1** | -2.287 | **PPL** | 1.587 | **B3GNT2** | -1.968 |
|  |  | **MAP3K7** | -2.285 | **BCL2L1** | 1.587 | **TBK1** | -1.966 |
|  |  | **ICAM1** | -2.285 | **CDK6** | 1.581 | **CUL2** | -1.949 |
|  |  | **LATS2** | -2.276 | **SHC1** | 1.58 | **APPL1** | -1.947 |
|  |  | **BIRC5** | -2.274 | **MGAT3** | 1.579 | **PTPN12** | -1.934 |
|  |  | **GFM1** | -2.27 | **MXD3** | 1.575 | **S100A11** | -1.932 |
|  |  | **CX3CL1** | -2.264 | **NME3** | 1.574 | **DAB2** | -1.919 |
|  |  | **PPP3CB** | -2.249 | **ELAVL1** | 1.574 | **SFN** | -1.916 |
|  |  | **TGIF1** | -2.236 | **SELK** | 1.573 | **PARG** | -1.915 |
|  |  | **MLL3** | -2.234 | **STX3** | 1.571 | **ZFP36** | -1.914 |
|  |  | **IL13RA1** | -2.222 | **MCM4** | 1.549 | **TNFAIP3** | -1.913 |
|  |  | **NUP98** | -2.219 | **DCLRE1A** | 1.547 | **TUSC2** | -1.907 |
|  |  | **MAP2K1** | -2.213 | **SEC61G** | 1.54 | **OSBPL2** | -1.907 |
|  |  | **FOSL1** | -2.21 | **FASN** | 1.536 | **PPM1A** | -1.897 |
|  |  | **LIN28B** | -2.204 | **AES** | 1.529 | **PLOD2** | -1.894 |
|  |  | **CENPF** | -2.199 | **PTGDS** | 1.527 | **KLF10** | -1.893 |
|  |  | **SOX8** | -2.198 | **SNX12** | 1.527 | **MAP3K7IP3** | -1.889 |
|  |  | **HEYL** | -2.175 | **SUMO1** | 1.523 | **CASP3** | -1.884 |
|  |  | **CSNK1G3** | -2.175 | **GLRX** | 1.521 | **SPRED1** | -1.882 |
|  |  | **NFKB1** | -2.167 | **NSA2** | 1.521 | **DYRK1A** | -1.877 |
|  |  | **CDK2** | -2.158 | **HES6** | 1.52 | **SGPL1** | -1.877 |
|  |  | **STAT4** | -2.128 | **MVP** | 1.518 | **ATG7** | -1.869 |
|  |  | **EBI3** | -2.121 | **PEG10** | 1.518 | **ELF1** | -1.868 |
|  |  | **SLC12A2** | -2.114 | **CISD1** | 1.518 | **OAS3** | -1.862 |
|  |  | **PDPK1** | -2.104 | **COX17** | 1.514 | **FBXO11** | -1.856 |
|  |  | **SMO** | -2.098 | **SLC44A4** | 1.514 | **MIB1** | -1.849 |
|  |  | **ACVR1** | -2.095 | **SLC25A33** | 1.51 | **INPPL1** | -1.849 |
|  |  | **LBR** | -2.09 | **EDF1** | 1.508 | **ASNS** | -1.844 |
|  |  | **DDX5** | -2.089 | **ULK1** | 1.501 | **PTX3** | -1.843 |
|  |  | **SGK1** | -2.077 | **ABCG1** | 1.5 | **PCYOX1** | -1.843 |
|  |  | **AMACR** | -2.07 |  |  | **PPARA** | -1.841 |
|  |  | **TPR** | -2.064 |  |  | **ABLIM1** | -1.833 |
|  |  | **UPP1** | -2.06 |  |  | **IRF1** | -1.833 |
|  |  | **RIPK1** | -2.06 |  |  | **PPARG** | -1.831 |
|  |  | **PIK3R2** | -2.06 |  |  | **DCBLD2** | -1.829 |
|  |  | **PIK3CA** | -2.054 |  |  | **TES** | -1.826 |
|  |  | **SLC20A1** | -2.054 |  |  | **CHEK2** | -1.824 |
|  |  | **FAT4** | -2.053 |  |  | **TRIM24** | -1.803 |
|  |  | **CUL4A** | -2.051 |  |  | **SMAD4** | -1.8 |
|  |  | **ZNF16** | -2.05 |  |  | **BAT3** | -1.795 |
|  |  | **IDE** | -2.049 |  |  | **CTNNBIP1** | -1.794 |
|  |  | **PTPN3** | -2.044 |  |  | **VHL** | -1.794 |
|  |  | **NCCRP1** | -2.027 |  |  | **E2F7** | -1.786 |
|  |  | **CUL1** | -2.025 |  |  | **ULK2** | -1.784 |
|  |  | **CDC14B** | -2.022 |  |  | **CCNG2** | -1.781 |
|  |  | **CXCL2** | -2.021 |  |  | **NPTX1** | -1.78 |
|  |  | **PTHLH** | -2.018 |  |  | **ABTB1** | -1.777 |
|  |  | **DSG2** | -2.015 |  |  | **TNFRSF1B** | -1.771 |
|  |  | **HS.570988** | -2.008 |  |  | **CYLD** | -1.771 |
|  |  | **IDH2** | -2.008 |  |  | **LIPA** | -1.77 |
|  |  | **YES1** | -2.004 |  |  | **VPS39** | -1.766 |
|  |  | **VGF** | -2.002 |  |  | **P4HA2** | -1.758 |
|  |  | **TIMP1** | -2.002 |  |  | **ARID4B** | -1.754 |
|  |  | **SPTBN1** | -2.002 |  |  | **IQGAP1** | -1.751 |
|  |  | **PHIP** | -1.999 |  |  | **NME6** | -1.746 |
|  |  | **MKNK1** | -1.998 |  |  | **USP16** | -1.741 |
|  |  | **S1PR3** | -1.994 |  |  | **TRIM21** | -1.739 |
|  |  | **DDX21** | -1.992 |  |  | **JARID2** | -1.736 |
|  |  | **USP47** | -1.99 |  |  | **GEM** | -1.736 |
|  |  | **GAB2** | -1.989 |  |  | **VAMP7** | -1.733 |
|  |  | **DUSP10** | -1.986 |  |  | **PARP10** | -1.723 |
|  |  | **UBE2A** | -1.985 |  |  | **FAM188A** | -1.722 |
|  |  | **HSPA4** | -1.984 |  |  | **IL28A** | -1.722 |
|  |  | **NAE1** | -1.982 |  |  | **DUSP1** | -1.72 |
|  |  | **HNRPK** | -1.981 |  |  | **PAK1IP1** | -1.72 |
|  |  | **ARFGAP3** | -1.98 |  |  | **CD40** | -1.717 |
|  |  | **MINA** | -1.976 |  |  | **TANK** | -1.717 |
|  |  | **ADAM17** | -1.975 |  |  | **AKTIP** | -1.717 |
|  |  | **CIAO1** | -1.972 |  |  | **ARID4A** | -1.715 |
|  |  | **SEMA4C** | -1.971 |  |  | **CYP2S1** | -1.712 |
|  |  | **PAPSS2** | -1.967 |  |  | **TNFRSF1A** | -1.708 |
|  |  | **CLN3** | -1.966 |  |  | **DNER** | -1.708 |
|  |  | **E2F3** | -1.964 |  |  | **HS.371609** | -1.7 |
|  |  | **NFYA** | -1.962 |  |  | **PPP2R5C** | -1.695 |
|  |  | **ARFGEF1** | -1.959 |  |  | **SRM** | -1.694 |
|  |  | **CYR61** | -1.957 |  |  | **EPHX2** | -1.69 |
|  |  | **SERTAD1** | -1.953 |  |  | **RAPGEF2** | -1.688 |
|  |  | **TRIM39** | -1.95 |  |  | **HIAT1** | -1.688 |
|  |  | **KIAA1429** | -1.944 |  |  | **EFNA1** | -1.684 |
|  |  | **TNFAIP8** | -1.942 |  |  | **OBFC2A** | -1.68 |
|  |  | **PTPRA** | -1.941 |  |  | **CDKN1A** | -1.678 |
|  |  | **SUV420H1** | -1.94 |  |  | **AXIN1** | -1.676 |
|  |  | **RPS6KA3** | -1.939 |  |  | **SESN1** | -1.675 |
|  |  | **CD164** | -1.937 |  |  | **SERPINA3** | -1.675 |
|  |  | **NCK1** | -1.935 |  |  | **CBLB** | -1.673 |
|  |  | **LAP3** | -1.934 |  |  | **DDX3X** | -1.669 |
|  |  | **PRKAR1A** | -1.933 |  |  | **PROS1** | -1.668 |
|  |  | **TIRAP** | -1.933 |  |  | **PPP2CA** | -1.667 |
|  |  | **PIK3C2A** | -1.931 |  |  | **TLR6** | -1.666 |
|  |  | **ZMYM2** | -1.931 |  |  | **TP53BP2** | -1.659 |
|  |  | **GCH1** | -1.93 |  |  | **CASK** | -1.659 |
|  |  | **UGCG** | -1.928 |  |  | **ENPP1** | -1.657 |
|  |  | **OPTN** | -1.926 |  |  | **ITPR1** | -1.653 |
|  |  | **CSNK2A1** | -1.924 |  |  | **RHOB** | -1.651 |
|  |  | **ITGAV** | -1.924 |  |  | **VLDLR** | -1.648 |
|  |  | **ACTB** | -1.921 |  |  | **PHF14** | -1.647 |
|  |  | **P4HA1** | -1.911 |  |  | **KEAP1** | -1.644 |
|  |  | **CITED2** | -1.909 |  |  | **STX2** | -1.641 |
|  |  | **PRMT6** | -1.908 |  |  | **RANBP9** | -1.641 |
|  |  | **KPNA2** | -1.905 |  |  | **PXMP3** | -1.639 |
|  |  | **IMPACT** | -1.904 |  |  | **MORC3** | -1.622 |
|  |  | **EXOC5** | -1.899 |  |  | **GABPB2** | -1.62 |
|  |  | **AMD1** | -1.898 |  |  | **MINPP1** | -1.62 |
|  |  | **GAD1** | -1.897 |  |  | **DYNC1H1** | -1.617 |
|  |  | **NPC1** | -1.897 |  |  | **TOP1** | -1.615 |
|  |  | **SPAST** | -1.896 |  |  | **KLF6** | -1.611 |
|  |  | **ZNF259** | -1.893 |  |  | **ADIPOR1** | -1.603 |
|  |  | **PRKCA** | -1.889 |  |  | **TAP1** | -1.602 |
|  |  | **DDX17** | -1.887 |  |  | **CAPRIN1** | -1.6 |
|  |  | **CCPG1** | -1.887 |  |  | **B3GNT5** | -1.599 |
|  |  | **RAB5A** | -1.883 |  |  | **LAT2** | -1.592 |
|  |  | **FOXQ1** | -1.88 |  |  | **SAV1** | -1.59 |
|  |  | **MAPK1** | -1.879 |  |  | **AATF** | -1.585 |
|  |  | **FNDC3B** | -1.879 |  |  | **TMEM127** | -1.584 |
|  |  | **MYD88** | -1.878 |  |  | **RNF4** | -1.58 |
|  |  | **KIAA0261** | -1.876 |  |  | **UBIAD1** | -1.579 |
|  |  | **TFDP1** | -1.875 |  |  | **FOXF2** | -1.578 |
|  |  | **ITGA2** | -1.873 |  |  | **RND3** | -1.577 |
|  |  | **ARHGAP1** | -1.873 |  |  | **CTNNA1** | -1.576 |
|  |  | **ACTL6A** | -1.873 |  |  | **PARK2** | -1.575 |
|  |  | **JUN** | -1.871 |  |  | **SMYD2** | -1.574 |
|  |  | **XRCC5** | -1.856 |  |  | **MXD1** | -1.572 |
|  |  | **STK38L** | -1.848 |  |  | **CSNK1A1** | -1.571 |
|  |  | **ADAM9** | -1.842 |  |  | **ABI1** | -1.569 |
|  |  | **FAS** | -1.837 |  |  | **RHOH** | -1.569 |
|  |  | **TLN1** | -1.837 |  |  | **CTCF** | -1.566 |
|  |  | **SS18** | -1.833 |  |  | **CSE1L** | -1.566 |
|  |  | **EML4** | -1.831 |  |  | **KIF23** | -1.565 |
|  |  | **KRAS** | -1.824 |  |  | **IGFBP4** | -1.559 |
|  |  | **PDGFC** | -1.82 |  |  | **IL28B** | -1.558 |
|  |  | **PPP1CA** | -1.818 |  |  | **FKTN** | -1.551 |
|  |  | **SHMT1** | -1.816 |  |  | **CDK8** | -1.549 |
|  |  | **DLGAP5** | -1.813 |  |  | **BACH2** | -1.546 |
|  |  | **AKIRIN1** | -1.812 |  |  | **TAF6** | -1.546 |
|  |  | **DDAH1** | -1.81 |  |  | **STRN** | -1.541 |
|  |  | **NAMPT** | -1.808 |  |  | **ABCC1** | -1.541 |
|  |  | **KIF20B** | -1.807 |  |  | **PTPN2** | -1.54 |
|  |  | **RBM3** | -1.798 |  |  | **TOPBP1** | -1.534 |
|  |  | **GLDC** | -1.797 |  |  | **RNF111** | -1.534 |
|  |  | **VEGFC** | -1.796 |  |  | **H2AFY** | -1.534 |
|  |  | **MIR21** | -1.795 |  |  | **IFT57** | -1.531 |
|  |  | **RECQL** | -1.794 |  |  | **RB1CC1** | -1.523 |
|  |  | **API5** | -1.794 |  |  | **PBRM1** | -1.523 |
|  |  | **STK39** | -1.793 |  |  | **TCF12** | -1.522 |
|  |  | **HS.334831** | -1.789 |  |  | **HNRNPL** | -1.522 |
|  |  | **CBX2** | -1.785 |  |  | **HES1** | -1.522 |
|  |  | **BCAT1** | -1.782 |  |  | **IVNS1ABP** | -1.519 |
|  |  | **LEPR** | -1.779 |  |  | **EMILIN2** | -1.518 |
|  |  | **LARP1** | -1.778 |  |  | **CDC2L1** | -1.516 |
|  |  | **RGPD8** | -1.776 |  |  | **IFIT3** | -1.515 |
|  |  | **RFC1** | -1.775 |  |  | **RBM5** | -1.514 |
|  |  | **BMPR1A** | -1.775 |  |  | **ATF2** | -1.513 |
|  |  | **ADAM10** | -1.774 |  |  | **FBLN1** | -1.513 |
|  |  | **CDC2L6** | -1.774 |  |  | **KRT19** | -1.511 |
|  |  | **ZBED1** | -1.774 |  |  | **SOX17** | -1.511 |
|  |  | **CD46** | -1.773 |  |  | **MAPT** | -1.51 |
|  |  | **ASCC3** | -1.769 |  |  | **CTBP2** | -1.508 |
|  |  | **ALCAM** | -1.768 |  |  | **DNM2** | -1.506 |
|  |  | **WRN** | -1.767 |  |  | **SOCS6** | -1.506 |
|  |  | **ABI2** | -1.765 |  |  | **DCUN1D3** | -1.504 |
|  |  | **CDC2** | -1.761 |  |  | **ATG5** | -1.503 |
|  |  | **AHR** | -1.759 |  |  | **MCRS1** | -1.502 |
|  |  | **POT1** | -1.759 |  |  |  |  |
|  |  | **VANGL1** | -1.758 |  |  |  |  |
|  |  | **ATMIN** | -1.758 |  |  |  |  |
|  |  | **EXTL3** | -1.758 |  |  |  |  |
|  |  | **BPNT1** | -1.756 |  |  |  |  |
|  |  | **MAP2K5** | -1.755 |  |  |  |  |
|  |  | **APP** | -1.751 |  |  |  |  |
|  |  | **RCAN1** | -1.75 |  |  |  |  |
|  |  | **PHCA** | -1.75 |  |  |  |  |
|  |  | **STAT5B** | -1.749 |  |  |  |  |
|  |  | **WWTR1** | -1.746 |  |  |  |  |
|  |  | **GCLC** | -1.744 |  |  |  |  |
|  |  | **LTB** | -1.743 |  |  |  |  |
|  |  | **FBXW11** | -1.737 |  |  |  |  |
|  |  | **NFKB2** | -1.737 |  |  |  |  |
|  |  | **SMARCB1** | -1.734 |  |  |  |  |
|  |  | **PLD1** | -1.734 |  |  |  |  |
|  |  | **CLIP1** | -1.732 |  |  |  |  |
|  |  | **MAP4K4** | -1.729 |  |  |  |  |
|  |  | **FER** | -1.721 |  |  |  |  |
|  |  | **USP22** | -1.721 |  |  |  |  |
|  |  | **PDE5A** | -1.718 |  |  |  |  |
|  |  | **ULBP1** | -1.717 |  |  |  |  |
|  |  | **GOLPH3** | -1.717 |  |  |  |  |
|  |  | **CERK** | -1.717 |  |  |  |  |
|  |  | **PSME3** | -1.716 |  |  |  |  |
|  |  | **FKBP1B** | -1.715 |  |  |  |  |
|  |  | **CCNDBP1** | -1.714 |  |  |  |  |
|  |  | **TRIO** | -1.712 |  |  |  |  |
|  |  | **PTGER4** | -1.712 |  |  |  |  |
|  |  | **USO1** | -1.712 |  |  |  |  |
|  |  | **HMGA1** | -1.711 |  |  |  |  |
|  |  | **FER1L3** | -1.71 |  |  |  |  |
|  |  | **F2R** | -1.706 |  |  |  |  |
|  |  | **LSM1** | -1.704 |  |  |  |  |
|  |  | **NCOA2** | -1.703 |  |  |  |  |
|  |  | **HMGCR** | -1.701 |  |  |  |  |
|  |  | **KATNA1** | -1.701 |  |  |  |  |
|  |  | **BCL2A1** | -1.7 |  |  |  |  |
|  |  | **PLCE1** | -1.7 |  |  |  |  |
|  |  | **RAB28** | -1.699 |  |  |  |  |
|  |  | **AURKA** | -1.697 |  |  |  |  |
|  |  | **FGFR4** | -1.693 |  |  |  |  |
|  |  | **KIF18A** | -1.688 |  |  |  |  |
|  |  | **MCMDC1** | -1.686 |  |  |  |  |
|  |  | **TOP1MT** | -1.686 |  |  |  |  |
|  |  | **ERBB2** | -1.683 |  |  |  |  |
|  |  | **PTPN13** | -1.681 |  |  |  |  |
|  |  | **BIRC6** | -1.68 |  |  |  |  |
|  |  | **SLC24A6** | -1.677 |  |  |  |  |
|  |  | **PLCG2** | -1.676 |  |  |  |  |
|  |  | **RIMS3** | -1.675 |  |  |  |  |
|  |  | **ERO1LB** | -1.673 |  |  |  |  |
|  |  | **KIAA0776** | -1.673 |  |  |  |  |
|  |  | **EXT1** | -1.671 |  |  |  |  |
|  |  | **HAS2** | -1.67 |  |  |  |  |
|  |  | **ZEB1** | -1.67 |  |  |  |  |
|  |  | **TP53BP1** | -1.667 |  |  |  |  |
|  |  | **ZNF451** | -1.667 |  |  |  |  |
|  |  | **RNF20** | -1.664 |  |  |  |  |
|  |  | **ANGPTL4** | -1.663 |  |  |  |  |
|  |  | **RALA** | -1.663 |  |  |  |  |
|  |  | **RFC3** | -1.663 |  |  |  |  |
|  |  | **EGFR** | -1.661 |  |  |  |  |
|  |  | **EPS8** | -1.659 |  |  |  |  |
|  |  | **MSH3** | -1.657 |  |  |  |  |
|  |  | **TAF9L** | -1.657 |  |  |  |  |
|  |  | **CALCRL** | -1.656 |  |  |  |  |
|  |  | **GPR3** | -1.652 |  |  |  |  |
|  |  | **GCNT2** | -1.646 |  |  |  |  |
|  |  | **PAK2** | -1.645 |  |  |  |  |
|  |  | **FIS1** | -1.645 |  |  |  |  |
|  |  | **MALT1** | -1.643 |  |  |  |  |
|  |  | **WWC1** | -1.642 |  |  |  |  |
|  |  | **FKBP1A** | -1.64 |  |  |  |  |
|  |  | **GNAQ** | -1.638 |  |  |  |  |
|  |  | **NEDD4L** | -1.637 |  |  |  |  |
|  |  | **PPIA** | -1.636 |  |  |  |  |
|  |  | **PIK3CB** | -1.636 |  |  |  |  |
|  |  | **KIF11** | -1.636 |  |  |  |  |
|  |  | **BARD1** | -1.635 |  |  |  |  |
|  |  | **OSBPL1A** | -1.63 |  |  |  |  |
|  |  | **NFATC3** | -1.629 |  |  |  |  |
|  |  | **RBCK1** | -1.626 |  |  |  |  |
|  |  | **CD59** | -1.625 |  |  |  |  |
|  |  | **RIPK2** | -1.624 |  |  |  |  |
|  |  | **MTDH** | -1.623 |  |  |  |  |
|  |  | **RAD50** | -1.619 |  |  |  |  |
|  |  | **ANXA2** | -1.619 |  |  |  |  |
|  |  | **DLL1** | -1.615 |  |  |  |  |
|  |  | **ACVR2A** | -1.614 |  |  |  |  |
|  |  | **C1GALT1C1** | -1.614 |  |  |  |  |
|  |  | **PLS3** | -1.608 |  |  |  |  |
|  |  | **CDC2L5** | -1.607 |  |  |  |  |
|  |  | **CNOT2** | -1.607 |  |  |  |  |
|  |  | **FNTB** | -1.606 |  |  |  |  |
|  |  | **E2F6** | -1.606 |  |  |  |  |
|  |  | **XRCC4** | -1.606 |  |  |  |  |
|  |  | **FGFR1OP** | -1.603 |  |  |  |  |
|  |  | **FYN** | -1.601 |  |  |  |  |
|  |  | **MLL5** | -1.601 |  |  |  |  |
|  |  | **ETV4** | -1.6 |  |  |  |  |
|  |  | **CCT2** | -1.597 |  |  |  |  |
|  |  | **TTLL4** | -1.596 |  |  |  |  |
|  |  | **ECT2** | -1.593 |  |  |  |  |
|  |  | **UBR5** | -1.592 |  |  |  |  |
|  |  | **EPAS1** | -1.59 |  |  |  |  |
|  |  | **PRCC** | -1.59 |  |  |  |  |
|  |  | **PRKD3** | -1.59 |  |  |  |  |
|  |  | **STT3B** | -1.59 |  |  |  |  |
|  |  | **NOD2** | -1.586 |  |  |  |  |
|  |  | **IFNAR2** | -1.586 |  |  |  |  |
|  |  | **CAPN2** | -1.585 |  |  |  |  |
|  |  | **CAMK2D** | -1.585 |  |  |  |  |
|  |  | **EIF2C3** | -1.584 |  |  |  |  |
|  |  | **STK24** | -1.583 |  |  |  |  |
|  |  | **CCNA2** | -1.583 |  |  |  |  |
|  |  | **TRADD** | -1.582 |  |  |  |  |
|  |  | **RAB1A** | -1.58 |  |  |  |  |
|  |  | **MAP3K7IP2** | -1.579 |  |  |  |  |
|  |  | **PIK3R1** | -1.578 |  |  |  |  |
|  |  | **GPR172B** | -1.578 |  |  |  |  |
|  |  | **STAG1** | -1.578 |  |  |  |  |
|  |  | **RPRD1B** | -1.578 |  |  |  |  |
|  |  | **PIK3C2B** | -1.576 |  |  |  |  |
|  |  | **MAPK13** | -1.572 |  |  |  |  |
|  |  | **DNAJA2** | -1.572 |  |  |  |  |
|  |  | **SLC2A1** | -1.571 |  |  |  |  |
|  |  | **SOCS2** | -1.568 |  |  |  |  |
|  |  | **RHOQ** | -1.568 |  |  |  |  |
|  |  | **JAK2** | -1.568 |  |  |  |  |
|  |  | **NUDCD3** | -1.564 |  |  |  |  |
|  |  | **THOC1** | -1.564 |  |  |  |  |
|  |  | **MAP3K4** | -1.563 |  |  |  |  |
|  |  | **TESK1** | -1.562 |  |  |  |  |
|  |  | **STAT6** | -1.561 |  |  |  |  |
|  |  | **TYMS** | -1.56 |  |  |  |  |
|  |  | **MSRA** | -1.558 |  |  |  |  |
|  |  | **EIF2B2** | -1.557 |  |  |  |  |
|  |  | **C1GALT1** | -1.557 |  |  |  |  |
|  |  | **SFRS5** | -1.556 |  |  |  |  |
|  |  | **MUC1** | -1.556 |  |  |  |  |
|  |  | **RECK** | -1.555 |  |  |  |  |
|  |  | **GJC1** | -1.552 |  |  |  |  |
|  |  | **ARHGEF6** | -1.551 |  |  |  |  |
|  |  | **ARNTL** | -1.55 |  |  |  |  |
|  |  | **RBBP9** | -1.55 |  |  |  |  |
|  |  | **STEAP2** | -1.547 |  |  |  |  |
|  |  | **HGF** | -1.545 |  |  |  |  |
|  |  | **KDM5B** | -1.543 |  |  |  |  |
|  |  | **AKR1C3** | -1.543 |  |  |  |  |
|  |  | **WASF2** | -1.54 |  |  |  |  |
|  |  | **TBX1** | -1.54 |  |  |  |  |
|  |  | **SMC3** | -1.54 |  |  |  |  |
|  |  | **BLM** | -1.538 |  |  |  |  |
|  |  | **MMP25** | -1.537 |  |  |  |  |
|  |  | **TICAM1** | -1.536 |  |  |  |  |
|  |  | **CISD2** | -1.533 |  |  |  |  |
|  |  | **CD2AP** | -1.532 |  |  |  |  |
|  |  | **TDG** | -1.531 |  |  |  |  |
|  |  | **TADA3** | -1.529 |  |  |  |  |
|  |  | **RNF144B** | -1.528 |  |  |  |  |
|  |  | **NFS1** | -1.525 |  |  |  |  |
|  |  | **SLC30A6** | -1.524 |  |  |  |  |
|  |  | **AKAP13** | -1.523 |  |  |  |  |
|  |  | **HIP1** | -1.52 |  |  |  |  |
|  |  | **BUB3** | -1.519 |  |  |  |  |
|  |  | **NRIP1** | -1.519 |  |  |  |  |
|  |  | **ARRB2** | -1.519 |  |  |  |  |
|  |  | **YEATS4** | -1.519 |  |  |  |  |
|  |  | **CASP6** | -1.519 |  |  |  |  |
|  |  | **RCL1** | -1.519 |  |  |  |  |
|  |  | **FRAP1** | -1.518 |  |  |  |  |
|  |  | **AKT2** | -1.516 |  |  |  |  |
|  |  | **ACSL4** | -1.515 |  |  |  |  |
|  |  | **CXCR4** | -1.515 |  |  |  |  |
|  |  | **PFKFB3** | -1.514 |  |  |  |  |
|  |  | **PALLD** | -1.512 |  |  |  |  |
|  |  | **AGGF1** | -1.507 |  |  |  |  |
|  |  | **EZH2** | -1.503 |  |  |  |  |
|  |  | **MAP2K4** | -1.503 |  |  |  |  |
|  |  | **ST5** | -1.502 |  |  |  |  |
|  |  | **GLB1** | -1.502 |  |  |  |  |
|  |  | **ASPM** | -1.501 |  |  |  |  |
|  |  | **STK38** | -1.501 |  |  |  |  |

B.

| **Expected** | | | | **Inversely related** | | | |
| --- | --- | --- | --- | --- | --- | --- | --- |
| **increased cell death** | | | | **decreased cell death** | | | |
| **up-genes (66)** | fold change | **down genes (393)** | fold change | **up-genes (94)** | fold change | **down genes (288)** | fold change |
| **TXNIP** | 5.257 | **RRM2** | -7.011 | **NUAK1** | 3.768 | **EGR1** | -15.086 |
| **SREBF1** | 4.275 | **IL8** | -6.204 | **DEPDC6** | 3.277 | **RAC1** | -8.138 |
| **SET** | 3.643 | **BIRC3** | -6.182 | **TFF3** | 3.153 | **CD69** | -7.186 |
| **HMGB2** | 2.987 | **TRIB1** | -4.97 | **LRPAP1** | 2.864 | **FOS** | -5.573 |
| **HMGB1** | 2.955 | **CTGF** | -4.591 | **SIP1** | 2.815 | **MX1** | -5.51 |
| **SMARCC1** | 2.9 | **CAST** | -4.411 | **STX8** | 2.777 | **GULP1** | -4.962 |
| **ITGB3BP** | 2.747 | **PLAU** | -4.394 | **PTPMT1** | 2.69 | **B4GALT5** | -4.263 |
| **NCOR2** | 2.551 | **CDCP1** | -4 | **HOXC6** | 2.663 | **DUSP6** | -4.233 |
| **EMP1** | 2.435 | **PPP2R2A** | -3.901 | **PLAC8** | 2.516 | **DDX58** | -4.085 |
| **FAM162A** | 2.329 | **WEE1** | -3.845 | **DAG1** | 2.452 | **STAT1** | -4.047 |
| **MYH9** | 2.289 | **VCL** | -3.829 | **DDIT4** | 2.389 | **TNFRSF21** | -4.036 |
| **H2AFX** | 2.155 | **CYP2J2** | -3.777 | **FBXO2** | 2.378 | **OLR1** | -4.027 |
| **NUMA1** | 2.124 | **NFKBIA** | -3.735 | **YWHAG** | 2.3 | **DKK1** | -4.018 |
| **DNAJA3** | 2.113 | **OPA1** | -3.536 | **S100A4** | 2.256 | **NFKBIZ** | -3.78 |
| **DPM3** | 2.007 | **SP3** | -3.521 | **ACO2** | 2.249 | **ITGB1** | -3.766 |
| **ACLY** | 2.006 | **CXCL1** | -3.471 | **SIGIRR** | 2.242 | **STEAP3** | -3.66 |
| **BOK** | 2.003 | **SRXN1** | -3.422 | **TAOK3** | 2.196 | **PHLDA1** | -3.657 |
| **ISG15** | 1.97 | **EGR2** | -3.392 | **HSPBP1** | 2.146 | **IFIH1** | -3.606 |
| **CDKN2C** | 1.935 | **SIAH2** | -3.389 | **RGS10** | 2.127 | **PRMT2** | -3.414 |
| **EGLN2** | 1.882 | **YWHAZ** | -3.362 | **WDR4** | 2.114 | **F2RL1** | -3.358 |
| **IL17RD** | 1.873 | **SLC4A7** | -3.353 | **SERPINH1** | 2.094 | **WASPIP** | -3.325 |
| **LAMP1** | 1.843 | **STK40** | -3.272 | **SRF** | 2.08 | **NQO1** | -3.309 |
| **IL10** | 1.834 | **SPRY2** | -3.196 | **MEF2D** | 2.078 | **DOCK1** | -3.257 |
| **HS1BP3** | 1.825 | **RAF1** | -3.186 | **PIM3** | 2.073 | **FRMD6** | -3.158 |
| **TGFBI** | 1.808 | **TGFBR2** | -3.154 | **CAMK2G** | 2.064 | **NRG1** | -3.129 |
| **LGALS7** | 1.803 | **VPS4B** | -3.147 | **EWSR1** | 2.041 | **PERP** | -2.924 |
| **LGALS1** | 1.802 | **NEK6** | -3.142 | **INTS1** | 2.028 | **TFPI** | -2.787 |
| **ETS1** | 1.787 | **LGMN** | -3.136 | **CBS** | 2.022 | **ANTXR2** | -2.773 |
| **RASSF6** | 1.787 | **KLK3** | -3.093 | **TBP** | 2.003 | **CTSL1** | -2.763 |
| **HIST1H1C** | 1.781 | **RRM2B** | -3.053 | **MSX1** | 1.973 | **LCN2** | -2.759 |
| **PRELID1** | 1.781 | **VASP** | -3.034 | **NDEL1** | 1.973 | **IL1R1** | -2.75 |
| **CSF2RA** | 1.777 | **CTNNB1** | -3.028 | **TPD52** | 1.951 | **AFAP1** | -2.7 |
| **PTRH2** | 1.754 | **PPP1R13L** | -3.01 | **HSPA1B** | 1.939 | **CASP2** | -2.648 |
| **FAU** | 1.737 | **GLUD1** | -2.966 | **PINK1** | 1.915 | **GPR37** | -2.639 |
| **CDC42** | 1.731 | **CD151** | -2.924 | **NOLC1** | 1.909 | **SMAD3** | -2.627 |
| **PRPS1** | 1.727 | **BIRC2** | -2.921 | **LSMD1** | 1.868 | **MAPK9** | -2.609 |
| **HRASLS** | 1.714 | **AKAP12** | -2.889 | **DYNLL1** | 1.854 | **PNPT1** | -2.597 |
| **AGR2** | 1.713 | **SERPINE1** | -2.835 | **TUB** | 1.812 | **STAM2** | -2.568 |
| **DAXX** | 1.703 | **EOMES** | -2.815 | **DMC1** | 1.803 | **EIF2AK2** | -2.549 |
| **S100A6** | 1.681 | **PLK2** | -2.805 | **CREB1** | 1.782 | **SERINC3** | -2.518 |
| **ENDOG** | 1.666 | **EIF4G2** | -2.741 | **UBQLN1** | 1.778 | **ATP2A2** | -2.512 |
| **CYBA** | 1.645 | **VAMP3** | -2.717 | **TSC22D3** | 1.778 | **DNM1L** | -2.506 |
| **CTSB** | 1.64 | **RB1** | -2.707 | **NKX6-2** | 1.774 | **IER3** | -2.506 |
| **ADRM1** | 1.633 | **TXNDC5** | -2.702 | **TRIAP1** | 1.743 | **CAPRIN2** | -2.477 |
| **TMEM107** | 1.631 | **ERCC1** | -2.684 | **E2F4** | 1.721 | **RASA1** | -2.476 |
| **PEBP1** | 1.63 | **ADAR** | -2.639 | **CDKN2D** | 1.717 | **IFNGR1** | -2.47 |
| **CDKN1C** | 1.62 | **NBN** | -2.614 | **NUF2** | 1.717 | **PIAS3** | -2.463 |
| **PPARBP** | 1.613 | **YAP1** | -2.596 | **LEP** | 1.704 | **RNASEL** | -2.461 |
| **CD24** | 1.6 | **PRKAA1** | -2.582 | **CALM3** | 1.695 | **JAK1** | -2.43 |
| **PRR7** | 1.586 | **NFE2L2** | -2.573 | **CBL** | 1.683 | **CCNB1** | -2.424 |
| **NFKBIB** | 1.581 | **AADACL1** | -2.568 | **PI4KB** | 1.667 | **CCDC6** | -2.416 |
| **CDK6** | 1.581 | **ABCC5** | -2.562 | **CBX4** | 1.662 | **TNF** | -2.402 |
| **SHC1** | 1.58 | **MCL1** | -2.526 | **MKL1** | 1.657 | **SCP2** | -2.396 |
| **NME3** | 1.574 | **CAV1** | -2.515 | **ILF3** | 1.652 | **TRIB3** | -2.368 |
| **UBE2L3** | 1.549 | **ERC1** | -2.476 | **ODC1** | 1.652 | **ZFYVE16** | -2.362 |
| **BRMS1** | 1.548 | **TPM3** | -2.476 | **STXBP1** | 1.648 | **HAS3** | -2.356 |
| **MRPL41** | 1.545 | **PIGA** | -2.451 | **HSPA5** | 1.648 | **MAP3K11** | -2.354 |
| **LAMA5** | 1.54 | **SGK3** | -2.441 | **TNNI3** | 1.639 | **PARP14** | -2.35 |
| **AES** | 1.529 | **ANXA4** | -2.427 | **IL11** | 1.634 | **LGALS8** | -2.334 |
| **PTGDS** | 1.527 | **DICER1** | -2.424 | **DIDO1** | 1.63 | **RAB22A** | -2.332 |
| **PRAF2** | 1.527 | **ABCE1** | -2.389 | **LMO4** | 1.627 | **OAS1** | -2.332 |
| **SUMO1** | 1.523 | **MBTPS1** | -2.388 | **VEGFB** | 1.627 | **SDC4** | -2.323 |
| **MAP3K12** | 1.522 | **CD44** | -2.382 | **ICMT** | 1.609 | **SLC25A24** | -2.323 |
| **GADD45A** | 1.519 | **REL** | -2.38 | **PLEC1** | 1.606 | **TFPI2** | -2.296 |
| **MVP** | 1.518 | **ITGB4** | -2.377 | **EI24** | 1.604 | **ICAM1** | -2.285 |
| **MT1F** | 1.503 | **NR2C2** | -2.373 | **OSCAR** | 1.602 | **LATS2** | -2.276 |
|  |  | **HIPK3** | -2.362 | **ERF** | 1.59 | **ZFAND5** | -2.273 |
|  |  | **BHLHB2** | -2.357 | **BCL2L1** | 1.587 | **PMP22** | -2.272 |
|  |  | **CBFB** | -2.357 | **MGAT3** | 1.579 | **CX3CL1** | -2.264 |
|  |  | **BTG2** | -2.355 | **YWHAE** | 1.576 | **USP12** | -2.251 |
|  |  | **ADH5** | -2.353 | **MXD3** | 1.575 | **NOTCH2** | -2.247 |
|  |  | **SELE** | -2.349 | **ARID1A** | 1.575 | **TICAM2** | -2.242 |
|  |  | **CES1** | -2.348 | **ELAVL1** | 1.574 | **CTH** | -2.236 |
|  |  | **PDE4D** | -2.344 | **GADD45GIP1** | 1.563 | **ICAM3** | -2.236 |
|  |  | **EIF2C2** | -2.342 | **EMD** | 1.558 | **TJP2** | -2.228 |
|  |  | **HS.128753** | -2.337 | **TAF10** | 1.553 | **CASP7** | -2.216 |
|  |  | **CYB5A** | -2.321 | **PKD1** | 1.553 | **XPR1** | -2.216 |
|  |  | **USP18** | -2.311 | **BCL2L12** | 1.552 | **FOSL1** | -2.21 |
|  |  | **CTTN** | -2.309 | **SEC61G** | 1.54 | **ITGA6** | -2.21 |
|  |  | **ATP7A** | -2.299 | **CALR** | 1.539 | **ROCK2** | -2.194 |
|  |  | **CHUK** | -2.299 | **GPI** | 1.538 | **MAP2K3** | -2.193 |
|  |  | **TRPC1** | -2.287 | **WFS1** | 1.537 | **BID** | -2.178 |
|  |  | **MAP3K7** | -2.285 | **UXT** | 1.536 | **PRKDC** | -2.171 |
|  |  | **SMARCA5** | -2.285 | **FASN** | 1.536 | **ELF4** | -2.159 |
|  |  | **SRI** | -2.279 | **DNAJB6** | 1.534 | **CDK2** | -2.158 |
|  |  | **BIRC5** | -2.274 | **HBXIP** | 1.531 | **CCDC109A** | -2.149 |
|  |  | **IPPK** | -2.263 | **EBAG9** | 1.53 | **SP110** | -2.147 |
|  |  | **PPP2R1B** | -2.252 | **MAPKAP1** | 1.529 | **WDR48** | -2.144 |
|  |  | **PPP3CB** | -2.249 | **HSPE1** | 1.521 | **STAT4** | -2.128 |
|  |  | **HSD17B4** | -2.229 | **PEG10** | 1.518 | **ATG4A** | -2.095 |
|  |  | **MTM1** | -2.229 | **APRT** | 1.514 | **DDX5** | -2.089 |
|  |  | **MAP2K1** | -2.213 | **GPX4** | 1.504 | **CKAP2** | -2.069 |
|  |  | **CENPF** | -2.199 | **MAFG** | 1.502 | **RIPK1** | -2.06 |
|  |  | **SOX8** | -2.198 | **ABCG1** | 1.5 | **PIK3R2** | -2.06 |
|  |  | **RELB** | -2.197 |  |  | **IDE** | -2.049 |
|  |  | **HADHA** | -2.197 |  |  | **NSMAF** | -2.038 |
|  |  | **IFI6** | -2.195 |  |  | **TMEM158** | -2.027 |
|  |  | **MERTK** | -2.188 |  |  | **SESN2** | -2.019 |
|  |  | **ZNF184** | -2.177 |  |  | **DSG2** | -2.015 |
|  |  | **HEYL** | -2.175 |  |  | **SENP2** | -2.008 |
|  |  | **SERP1** | -2.168 |  |  | **YES1** | -2.004 |
|  |  | **NFKB1** | -2.167 |  |  | **VGF** | -2.002 |
|  |  | **CAT** | -2.164 |  |  | **SPTBN1** | -2.002 |
|  |  | **HS.532698** | -2.162 |  |  | **HMOX2** | -1.996 |
|  |  | **MSRB3** | -2.161 |  |  | **ZAK** | -1.993 |
|  |  | **PDE4B** | -2.127 |  |  | **NAE1** | -1.982 |
|  |  | **SLC12A2** | -2.114 |  |  | **SLC25A38** | -1.979 |
|  |  | **PDPK1** | -2.104 |  |  | **WTAP** | -1.972 |
|  |  | **RABGGTA** | -2.103 |  |  | **E2F3** | -1.964 |
|  |  | **SMO** | -2.098 |  |  | **SNAP25** | -1.957 |
|  |  | **RBL2** | -2.096 |  |  | **TIA1** | -1.955 |
|  |  | **PAFAH2** | -2.093 |  |  | **APPL1** | -1.947 |
|  |  | **DSP** | -2.088 |  |  | **PTPRA** | -1.941 |
|  |  | **SGK1** | -2.077 |  |  | **OMA1** | -1.939 |
|  |  | **CCNI** | -2.077 |  |  | **NCK1** | -1.935 |
|  |  | **AMACR** | -2.07 |  |  | **PRKAR1A** | -1.933 |
|  |  | **FIGNL1** | -2.066 |  |  | **TIRAP** | -1.933 |
|  |  | **TPR** | -2.064 |  |  | **GCH1** | -1.93 |
|  |  | **PIK3CA** | -2.054 |  |  | **PPP2R5A** | -1.925 |
|  |  | **SLC20A1** | -2.054 |  |  | **CSNK2A1** | -1.924 |
|  |  | **FAT4** | -2.053 |  |  | **TBCCD1** | -1.922 |
|  |  | **ZNF16** | -2.05 |  |  | **DAB2** | -1.919 |
|  |  | **ANXA7** | -2.048 |  |  | **PARG** | -1.915 |
|  |  | **HS.24119** | -2.037 |  |  | **ZFP36** | -1.914 |
|  |  | **UBR2** | -2.032 |  |  | **PDCD6IP** | -1.899 |
|  |  | **JUNB** | -2.031 |  |  | **PPM1A** | -1.897 |
|  |  | **CUL1** | -2.025 |  |  | **CASP3** | -1.884 |
|  |  | **PON2** | -2.023 |  |  | **ZMYND11** | -1.88 |
|  |  | **CXCL2** | -2.021 |  |  | **MYD88** | -1.878 |
|  |  | **PTHLH** | -2.018 |  |  | **SGPL1** | -1.877 |
|  |  | **KIFAP3** | -2.015 |  |  | **TFDP1** | -1.875 |
|  |  | **GLO1** | -2.01 |  |  | **ACTL6A** | -1.873 |
|  |  | **HS.570988** | -2.008 |  |  | **MAP3K8** | -1.871 |
|  |  | **IDH2** | -2.008 |  |  | **JUN** | -1.871 |
|  |  | **TIMP1** | -2.002 |  |  | **ATG7** | -1.869 |
|  |  | **PHIP** | -1.999 |  |  | **OAS3** | -1.862 |
|  |  | **MKNK1** | -1.998 |  |  | **RFK** | -1.856 |
|  |  | **S1PR3** | -1.994 |  |  | **WWC3** | -1.855 |
|  |  | **NSF** | -1.992 |  |  | **MIB1** | -1.849 |
|  |  | **USP47** | -1.99 |  |  | **PSEN2** | -1.848 |
|  |  | **GAB2** | -1.989 |  |  | **QKI** | -1.845 |
|  |  | **DUSP10** | -1.986 |  |  | **TLR1** | -1.84 |
|  |  | **HSPA4** | -1.984 |  |  | **TFDP2** | -1.84 |
|  |  | **HNRPK** | -1.981 |  |  | **FAS** | -1.837 |
|  |  | **ADAM17** | -1.975 |  |  | **IRF1** | -1.833 |
|  |  | **TBK1** | -1.966 |  |  | **THAP1** | -1.833 |
|  |  | **TOP2B** | -1.966 |  |  | **ANKRD1** | -1.831 |
|  |  | **CLN3** | -1.966 |  |  | **PPARG** | -1.831 |
|  |  | **TAX1BP1** | -1.963 |  |  | **KRAS** | -1.824 |
|  |  | **NFYA** | -1.962 |  |  | **CHEK2** | -1.824 |
|  |  | **CYR61** | -1.957 |  |  | **PPP1CA** | -1.818 |
|  |  | **SCARB2** | -1.95 |  |  | **ATP1A1** | -1.807 |
|  |  | **TRIM39** | -1.95 |  |  | **SMAD4** | -1.8 |
|  |  | **CUL2** | -1.949 |  |  | **FAF1** | -1.798 |
|  |  | **TNFAIP8** | -1.942 |  |  | **BAT3** | -1.795 |
|  |  | **RPS6KA3** | -1.939 |  |  | **VHL** | -1.794 |
|  |  | **SOX21** | -1.939 |  |  | **TSC22D2** | -1.791 |
|  |  | **CD164** | -1.937 |  |  | **ULK2** | -1.784 |
|  |  | **S100A11** | -1.932 |  |  | **GSR** | -1.78 |
|  |  | **ZMYM2** | -1.931 |  |  | **NPTX1** | -1.78 |
|  |  | **PPP1R2** | -1.93 |  |  | **GMCL1** | -1.773 |
|  |  | **UGCG** | -1.928 |  |  | **TNFRSF1B** | -1.771 |
|  |  | **OPTN** | -1.926 |  |  | **CYLD** | -1.771 |
|  |  | **ITGAV** | -1.924 |  |  | **LIPA** | -1.77 |
|  |  | **BBS2** | -1.918 |  |  | **CCNC** | -1.763 |
|  |  | **SFN** | -1.916 |  |  | **CDC2** | -1.761 |
|  |  | **DCAF7** | -1.914 |  |  | **MAP2K5** | -1.755 |
|  |  | **APIP** | -1.914 |  |  | **APP** | -1.751 |
|  |  | **TNFAIP3** | -1.913 |  |  | **XPC** | -1.75 |
|  |  | **CITED2** | -1.909 |  |  | **STAP2** | -1.744 |
|  |  | **KPNA2** | -1.905 |  |  | **LTB** | -1.743 |
|  |  | **LRP8** | -1.898 |  |  | **TRIM21** | -1.739 |
|  |  | **NPC1** | -1.897 |  |  | **UACA** | -1.732 |
|  |  | **ZNF259** | -1.893 |  |  | **SH3RF1** | -1.732 |
|  |  | **PRKCA** | -1.889 |  |  | **MAP4K4** | -1.729 |
|  |  | **THOC6** | -1.889 |  |  | **PKN2** | -1.727 |
|  |  | **HDAC9** | -1.882 |  |  | **PDE5A** | -1.718 |
|  |  | **MAPK1** | -1.879 |  |  | **ULBP1** | -1.717 |
|  |  | **KIAA0261** | -1.876 |  |  | **CD40** | -1.717 |
|  |  | **ITGA2** | -1.873 |  |  | **PSME3** | -1.716 |
|  |  | **ARHGAP1** | -1.873 |  |  | **PTGER4** | -1.712 |
|  |  | **DEK** | -1.87 |  |  | **SLC47A1** | -1.71 |
|  |  | **XRCC5** | -1.856 |  |  | **SDC1** | -1.709 |
|  |  | **ATP2B1** | -1.852 |  |  | **ATG16L1** | -1.709 |
|  |  | **ASNS** | -1.844 |  |  | **TNFRSF1A** | -1.708 |
|  |  | **PPARA** | -1.841 |  |  | **F2R** | -1.706 |
|  |  | **VPS13A** | -1.84 |  |  | **ATXN3** | -1.705 |
|  |  | **SERPINI1** | -1.833 |  |  | **HS.371609** | -1.7 |
|  |  | **VPS41** | -1.823 |  |  | **HS.4988** | -1.698 |
|  |  | **PDGFC** | -1.82 |  |  | **ALDH1A3** | -1.697 |
|  |  | **GRB10** | -1.82 |  |  | **FGFR4** | -1.693 |
|  |  | **KIF14** | -1.816 |  |  | **MBD1** | -1.689 |
|  |  | **ANXA5** | -1.814 |  |  | **M6PR** | -1.688 |
|  |  | **SETX** | -1.814 |  |  | **RAPGEF2** | -1.688 |
|  |  | **SPG7** | -1.812 |  |  | **PTPN13** | -1.681 |
|  |  | **NAMPT** | -1.808 |  |  | **AXIN1** | -1.676 |
|  |  | **TRIM24** | -1.803 |  |  | **DDX3X** | -1.669 |
|  |  | **RBM3** | -1.798 |  |  | **APOBEC3B** | -1.667 |
|  |  | **VEGFC** | -1.796 |  |  | **PPP2CA** | -1.667 |
|  |  | **BCL2L13** | -1.795 |  |  | **GFPT1** | -1.666 |
|  |  | **MIR21** | -1.795 |  |  | **TP53BP2** | -1.659 |
|  |  | **RECQL** | -1.794 |  |  | **TNFRSF10A** | -1.657 |
|  |  | **CTNNBIP1** | -1.794 |  |  | **ITPR1** | -1.653 |
|  |  | **API5** | -1.794 |  |  | **RHOB** | -1.651 |
|  |  | **HS.334831** | -1.789 |  |  | **PAK2** | -1.645 |
|  |  | **DHX9** | -1.788 |  |  | **FIS1** | -1.645 |
|  |  | **E2F7** | -1.786 |  |  | **PRKRA** | -1.645 |
|  |  | **SON** | -1.782 |  |  | **KEAP1** | -1.644 |
|  |  | **LEPR** | -1.779 |  |  | **MALT1** | -1.643 |
|  |  | **NPHS1** | -1.777 |  |  | **EBF4** | -1.643 |
|  |  | **RGPD8** | -1.776 |  |  | **RANBP9** | -1.641 |
|  |  | **RFC1** | -1.775 |  |  | **FKBP1A** | -1.64 |
|  |  | **CD46** | -1.773 |  |  | **GNAQ** | -1.638 |
|  |  | **LASS6** | -1.769 |  |  | **SEC23B** | -1.637 |
|  |  | **ALCAM** | -1.768 |  |  | **BARD1** | -1.635 |
|  |  | **AHR** | -1.759 |  |  | **NLRX1** | -1.631 |
|  |  | **POT1** | -1.759 |  |  | **DEPDC1** | -1.63 |
|  |  | **ATMIN** | -1.758 |  |  | **MIR302C** | -1.629 |
|  |  | **IRF2** | -1.752 |  |  | **ARHGEF7** | -1.624 |
|  |  | **ERCC5** | -1.751 |  |  | **RIPK2** | -1.624 |
|  |  | **RCAN1** | -1.75 |  |  | **SUB1** | -1.621 |
|  |  | **STAT5B** | -1.749 |  |  | **DDX20** | -1.62 |
|  |  | **SLC40A1** | -1.749 |  |  | **SRPX** | -1.619 |
|  |  | **PRKAA2** | -1.745 |  |  | **TOP1** | -1.615 |
|  |  | **GCLC** | -1.744 |  |  | **FYN** | -1.601 |
|  |  | **SLC9A4** | -1.743 |  |  | **ECT2** | -1.593 |
|  |  | **SNX7** | -1.741 |  |  | **SAV1** | -1.59 |
|  |  | **UBE2V1** | -1.737 |  |  | **IFNAR2** | -1.586 |
|  |  | **NFKB2** | -1.737 |  |  | **CAPN2** | -1.585 |
|  |  | **GEM** | -1.736 |  |  | **STK24** | -1.583 |
|  |  | **FBXL5** | -1.734 |  |  | **HIP2** | -1.583 |
|  |  | **PLD1** | -1.734 |  |  | **TRADD** | -1.582 |
|  |  | **PARP16** | -1.732 |  |  | **ATP6AP2** | -1.582 |
|  |  | **CCT6A** | -1.729 |  |  | **RAB1A** | -1.58 |
|  |  | **LASS2** | -1.724 |  |  | **MAL** | -1.579 |
|  |  | **IL28A** | -1.722 |  |  | **FOXF2** | -1.578 |
|  |  | **FER** | -1.721 |  |  | **STAG1** | -1.578 |
|  |  | **USP22** | -1.721 |  |  | **MELK** | -1.578 |
|  |  | **DUSP1** | -1.72 |  |  | **CTNNA1** | -1.576 |
|  |  | **PLS1** | -1.718 |  |  | **MAPK13** | -1.572 |
|  |  | **DPYD** | -1.718 |  |  | **RHOH** | -1.569 |
|  |  | **TANK** | -1.717 |  |  | **CSE1L** | -1.566 |
|  |  | **CERK** | -1.717 |  |  | **THOC1** | -1.564 |
|  |  | **RNF19A** | -1.716 |  |  | **ARL8B** | -1.562 |
|  |  | **SQSTM1** | -1.716 |  |  | **ING3** | -1.561 |
|  |  | **XPO1** | -1.714 |  |  | **CCNT1** | -1.558 |
|  |  | **NEK1** | -1.714 |  |  | **SPATA2** | -1.558 |
|  |  | **HMGA1** | -1.711 |  |  | **RECK** | -1.555 |
|  |  | **TCERG1** | -1.706 |  |  | **KLRF1** | -1.555 |
|  |  | **TMBIM6** | -1.703 |  |  | **TRIM13** | -1.554 |
|  |  | **NCOA2** | -1.703 |  |  | **SH2D4A** | -1.553 |
|  |  | **HMGCR** | -1.701 |  |  | **ARHGEF6** | -1.551 |
|  |  | **BCL2A1** | -1.7 |  |  | **SIK1** | -1.547 |
|  |  | **PSMB8** | -1.699 |  |  | **BACH2** | -1.546 |
|  |  | **RAB28** | -1.699 |  |  | **TAF6** | -1.546 |
|  |  | **AURKA** | -1.697 |  |  | **AKR1C3** | -1.543 |
|  |  | **PPP2R5C** | -1.695 |  |  | **PTPN2** | -1.54 |
|  |  | **NDST1** | -1.695 |  |  | **BLM** | -1.538 |
|  |  | **EFNA1** | -1.684 |  |  | **TICAM1** | -1.536 |
|  |  | **ERBB2** | -1.683 |  |  | **ARHGEF3** | -1.532 |
|  |  | **GPHN** | -1.681 |  |  | **IFT57** | -1.531 |
|  |  | **OBFC2A** | -1.68 |  |  | **RNF144B** | -1.528 |
|  |  | **BIRC6** | -1.68 |  |  | **DEDD** | -1.524 |
|  |  | **CDKN1A** | -1.678 |  |  | **TCF12** | -1.522 |
|  |  | **PLCG2** | -1.676 |  |  | **HES1** | -1.522 |
|  |  | **FAM134B** | -1.676 |  |  | **SHQ1** | -1.522 |
|  |  | **SERPINA3** | -1.675 |  |  | **TRMT11** | -1.521 |
|  |  | **CBLB** | -1.673 |  |  | **HIP1** | -1.52 |
|  |  | **PEX11B** | -1.672 |  |  | **CASP6** | -1.519 |
|  |  | **HAS2** | -1.67 |  |  | **EMILIN2** | -1.518 |
|  |  | **ZEB1** | -1.67 |  |  | **CXCR4** | -1.515 |
|  |  | **PROS1** | -1.668 |  |  | **AIFM1** | -1.514 |
|  |  | **TP53BP1** | -1.667 |  |  | **RBM5** | -1.514 |
|  |  | **TLR6** | -1.666 |  |  | **ATF2** | -1.513 |
|  |  | **PDE9A** | -1.666 |  |  | **MAPT** | -1.51 |
|  |  | **VAC14** | -1.664 |  |  | **MCOLN3** | -1.51 |
|  |  | **ANGPTL4** | -1.663 |  |  | **LASS5** | -1.51 |
|  |  | **AVEN** | -1.663 |  |  | **IL17RA** | -1.508 |
|  |  | **PRKAG1** | -1.661 |  |  | **DNM2** | -1.506 |
|  |  | **EGFR** | -1.661 |  |  | **REPS2** | -1.506 |
|  |  | **MSH3** | -1.657 |  |  | **DCUN1D3** | -1.504 |
|  |  | **TAF9L** | -1.657 |  |  | **SPAG5** | -1.503 |
|  |  | **CALCRL** | -1.656 |  |  | **ZNF274** | -1.503 |
|  |  | **ST3GAL1** | -1.656 |  |  | **ATG5** | -1.503 |
|  |  | **FSTL1** | -1.654 |  |  | **MAP2K4** | -1.503 |
|  |  | **BRE** | -1.651 |  |  | **CCBL1** | -1.502 |
|  |  | **HLA-F** | -1.647 |  |  | **ADRB1** | -1.501 |
|  |  | **PPIA** | -1.636 |  |  | **STK38** | -1.501 |
|  |  | **PIK3CB** | -1.636 |  |  |  |  |
|  |  | **KIF11** | -1.636 |  |  |  |  |
|  |  | **CHMP2A** | -1.634 |  |  |  |  |
|  |  | **GLIS3** | -1.628 |  |  |  |  |
|  |  | **RBCK1** | -1.626 |  |  |  |  |
|  |  | **UBA3** | -1.625 |  |  |  |  |
|  |  | **CD59** | -1.625 |  |  |  |  |
|  |  | **FANCL** | -1.625 |  |  |  |  |
|  |  | **MTDH** | -1.623 |  |  |  |  |
|  |  | **ZFP82** | -1.623 |  |  |  |  |
|  |  | **IFNGR2** | -1.62 |  |  |  |  |
|  |  | **RAD50** | -1.619 |  |  |  |  |
|  |  | **ANXA2** | -1.619 |  |  |  |  |
|  |  | **SEPN1** | -1.619 |  |  |  |  |
|  |  | **DYNC1H1** | -1.617 |  |  |  |  |
|  |  | **IREB2** | -1.616 |  |  |  |  |
|  |  | **DLL1** | -1.615 |  |  |  |  |
|  |  | **KLF6** | -1.611 |  |  |  |  |
|  |  | **CPEB4** | -1.611 |  |  |  |  |
|  |  | **RHBDD1** | -1.61 |  |  |  |  |
|  |  | **EPB41** | -1.608 |  |  |  |  |
|  |  | **CNOT2** | -1.607 |  |  |  |  |
|  |  | **E2F6** | -1.606 |  |  |  |  |
|  |  | **XRCC4** | -1.606 |  |  |  |  |
|  |  | **BAZ1A** | -1.605 |  |  |  |  |
|  |  | **CAPRIN1** | -1.6 |  |  |  |  |
|  |  | **ATP2B4** | -1.599 |  |  |  |  |
|  |  | **HEXB** | -1.594 |  |  |  |  |
|  |  | **PCID2** | -1.593 |  |  |  |  |
|  |  | **NPAS2** | -1.592 |  |  |  |  |
|  |  | **EXOC2** | -1.592 |  |  |  |  |
|  |  | **CCDC47** | -1.592 |  |  |  |  |
|  |  | **CKAP5** | -1.592 |  |  |  |  |
|  |  | **EPAS1** | -1.59 |  |  |  |  |
|  |  | **PRCC** | -1.59 |  |  |  |  |
|  |  | **PRKD3** | -1.59 |  |  |  |  |
|  |  | **FBXO5** | -1.587 |  |  |  |  |
|  |  | **SFRS2B** | -1.587 |  |  |  |  |
|  |  | **AATF** | -1.585 |  |  |  |  |
|  |  | **EIF2C3** | -1.584 |  |  |  |  |
|  |  | **MAN2A1** | -1.582 |  |  |  |  |
|  |  | **DCTN1** | -1.581 |  |  |  |  |
|  |  | **RNF4** | -1.58 |  |  |  |  |
|  |  | **MAP3K7IP2** | -1.579 |  |  |  |  |
|  |  | **PIK3R1** | -1.578 |  |  |  |  |
|  |  | **RND3** | -1.577 |  |  |  |  |
|  |  | **PARK2** | -1.575 |  |  |  |  |
|  |  | **GPN1** | -1.574 |  |  |  |  |
|  |  | **MXD1** | -1.572 |  |  |  |  |
|  |  | **SLC2A1** | -1.571 |  |  |  |  |
|  |  | **PAF1** | -1.57 |  |  |  |  |
|  |  | **SOCS2** | -1.568 |  |  |  |  |
|  |  | **JAK2** | -1.568 |  |  |  |  |
|  |  | **CTCF** | -1.566 |  |  |  |  |
|  |  | **NUDCD3** | -1.564 |  |  |  |  |
|  |  | **MAP3K4** | -1.563 |  |  |  |  |
|  |  | **STAT6** | -1.561 |  |  |  |  |
|  |  | **SLC29A2** | -1.56 |  |  |  |  |
|  |  | **TYMS** | -1.56 |  |  |  |  |
|  |  | **IGFBP4** | -1.559 |  |  |  |  |
|  |  | **MUC1** | -1.556 |  |  |  |  |
|  |  | **LIN7C** | -1.555 |  |  |  |  |
|  |  | **GJC1** | -1.552 |  |  |  |  |
|  |  | **CDK8** | -1.549 |  |  |  |  |
|  |  | **CERKL** | -1.549 |  |  |  |  |
|  |  | **MYO6** | -1.548 |  |  |  |  |
|  |  | **MCPH1** | -1.547 |  |  |  |  |
|  |  | **SPINT1** | -1.545 |  |  |  |  |
|  |  | **HGF** | -1.545 |  |  |  |  |
|  |  | **ADA** | -1.544 |  |  |  |  |
|  |  | **RRAS2** | -1.544 |  |  |  |  |
|  |  | **TBC1D15** | -1.543 |  |  |  |  |
|  |  | **ABCC1** | -1.541 |  |  |  |  |
|  |  | **TOPBP1** | -1.534 |  |  |  |  |
|  |  | **CISD2** | -1.533 |  |  |  |  |
|  |  | **PPID** | -1.532 |  |  |  |  |
|  |  | **MTMR2** | -1.532 |  |  |  |  |
|  |  | **CD2AP** | -1.532 |  |  |  |  |
|  |  | **ATP11C** | -1.532 |  |  |  |  |
|  |  | **DPP8** | -1.526 |  |  |  |  |
|  |  | **TMX1** | -1.524 |  |  |  |  |
|  |  | **RB1CC1** | -1.523 |  |  |  |  |
|  |  | **ARRB2** | -1.519 |  |  |  |  |
|  |  | **IVNS1ABP** | -1.519 |  |  |  |  |
|  |  | **YEATS4** | -1.519 |  |  |  |  |
|  |  | **FRAP1** | -1.518 |  |  |  |  |
|  |  | **AKT2** | -1.516 |  |  |  |  |
|  |  | **ACSL4** | -1.515 |  |  |  |  |
|  |  | **IFIT3** | -1.515 |  |  |  |  |
|  |  | **PLTP** | -1.515 |  |  |  |  |
|  |  | **FBLN1** | -1.513 |  |  |  |  |
|  |  | **PALLD** | -1.512 |  |  |  |  |
|  |  | **SORBS2** | -1.512 |  |  |  |  |
|  |  | **KRT19** | -1.511 |  |  |  |  |
|  |  | **CTBP2** | -1.508 |  |  |  |  |
|  |  | **WASF1** | -1.507 |  |  |  |  |
|  |  | **AGGF1** | -1.507 |  |  |  |  |
|  |  | **TXNRD1** | -1.506 |  |  |  |  |
|  |  | **ATP2C1** | -1.506 |  |  |  |  |
|  |  | **PCNA** | -1.505 |  |  |  |  |
|  |  | **EZH2** | -1.503 |  |  |  |  |
|  |  | **GSTA2** | -1.503 |  |  |  |  |
|  |  | **EFEMP1** | -1.502 |  |  |  |  |
|  |  | **MCRS1** | -1.502 |  |  |  |  |

C.

| **Expected** | | |  | **Inversely related** | | |  |
| --- | --- | --- | --- | --- | --- | --- | --- |
| **decreased migration** | | |  | **increased migration** | | |  |
| **up-genes (7)** | fold change | **down genes (122)** | fold change | **up-genes (22)** | fold change | **down genes (23)** | fold change |
| ***MYH9*** | 2.289 | **EGR1** | -15.086 | **C1QBP** | 3.473 | **RNF144** | -4.078 |
| ***DNAJA3*** | 2.113 | **RAC1** | -8.138 | **DPAGT1** | 2.397 | **STAT1** | -4.047 |
| ***DPYSL2*** | 1.725 | **IL8** | -6.204 | **S100A4** | 2.256 | **ARRDC3** | -3.535 |
| ***CMTM8*** | 1.706 | **CTGF** | -4.591 | **ACTN4** | 1.946 | **LAMC2** | -3.497 |
| ***BRMS1*** | 1.548 | **PLAU** | -4.394 | **ARF1** | 1.885 | **SPRY2** | -3.196 |
| ***LAMA5*** | 1.54 | **DDX58** | -4.085 | **DVL2** | 1.826 | **VASP** | -3.034 |
| ***DNAJB6*** | 1.534 | **CDCP1** | -4 | **PPFIA1** | 1.821 | **TPM3** | -2.476 |
|  |  | **SDCBP** | -3.874 | **LGALS1** | 1.802 | **RASA1** | -2.476 |
|  |  | **CYP2J2** | -3.777 | **ETS1** | 1.787 | **CAT** | -2.164 |
|  |  | **ITGB1** | -3.766 | **MAPK8IP3** | 1.765 | **EBI3** | -2.121 |
|  |  | **E2F5** | -3.714 | **CDC42** | 1.731 | **TIMP1** | -2.002 |
|  |  | **F2RL1** | -3.358 | **DYRK1B** | 1.716 | **ASNS** | -1.844 |
|  |  | **DOCK1** | -3.257 | **LEP** | 1.704 | **ADAM9** | -1.842 |
|  |  | **SSH1** | -3.222 | **MTA1** | 1.691 | **FAF1** | -1.798 |
|  |  | **NRG1** | -3.129 | **HS.535028** | 1.67 | **SMARCB1** | -1.734 |
|  |  | **CTNNB1** | -3.028 | **ILF3** | 1.652 | **FAM188A** | -1.722 |
|  |  | **CD151** | -2.924 | **DIDO1** | 1.63 | **WDR44** | -1.679 |
|  |  | **CCL20** | -2.862 | **VEGFB** | 1.627 | **GNAI1** | -1.618 |
|  |  | **SERPINE1** | -2.835 | **CD24** | 1.6 | **PARK2** | -1.575 |
|  |  | **BMPR2** | -2.784 | **CDC25B** | 1.597 | **IGFBP4** | -1.559 |
|  |  | **AFAP1** | -2.7 | **SHC1** | 1.58 | **ARHGAP21** | -1.519 |
|  |  | **SMAD3** | -2.627 | **HES6** | 1.52 | **PALLD** | -1.512 |
|  |  | **EIF2AK2** | -2.549 |  |  | **KRT19** | -1.511 |
|  |  | **ACTA2** | -2.542 |  |  |  |  |
|  |  | **CAV1** | -2.515 |  |  |  |  |
|  |  | **CD81** | -2.482 |  |  |  |  |
|  |  | **JAK1** | -2.43 |  |  |  |  |
|  |  | **TNF** | -2.402 |  |  |  |  |
|  |  | **CD44** | -2.382 |  |  |  |  |
|  |  | **HAS3** | -2.356 |  |  |  |  |
|  |  | **HS.128753** | -2.337 |  |  |  |  |
|  |  | **CTTN** | -2.309 |  |  |  |  |
|  |  | **MAP3K7** | -2.285 |  |  |  |  |
|  |  | **CX3CL1** | -2.264 |  |  |  |  |
|  |  | **NOTCH2** | -2.247 |  |  |  |  |
|  |  | **MAP2K1** | -2.213 |  |  |  |  |
|  |  | **ITGA6** | -2.21 |  |  |  |  |
|  |  | **FOSL1** | -2.21 |  |  |  |  |
|  |  | **LIN28B** | -2.204 |  |  |  |  |
|  |  | **MERTK** | -2.188 |  |  |  |  |
|  |  | **SLC12A2** | -2.114 |  |  |  |  |
|  |  | **CUL4A** | -2.051 |  |  |  |  |
|  |  | **RAB21** | -2.018 |  |  |  |  |
|  |  | **PTHLH** | -2.018 |  |  |  |  |
|  |  | **VGF** | -2.002 |  |  |  |  |
|  |  | **S1PR3** | -1.994 |  |  |  |  |
|  |  | **GAB2** | -1.989 |  |  |  |  |
|  |  | **HNRPK** | -1.981 |  |  |  |  |
|  |  | **ADAM17** | -1.975 |  |  |  |  |
|  |  | **ARFGEF1** | -1.959 |  |  |  |  |
|  |  | **CYR61** | -1.957 |  |  |  |  |
|  |  | **TNFAIP8** | -1.942 |  |  |  |  |
|  |  | **RPS6KA3** | -1.939 |  |  |  |  |
|  |  | **ITGAV** | -1.924 |  |  |  |  |
|  |  | **DAB2** | -1.919 |  |  |  |  |
|  |  | **SFN** | -1.916 |  |  |  |  |
|  |  | **PRMT6** | -1.908 |  |  |  |  |
|  |  | **KPNA2** | -1.905 |  |  |  |  |
|  |  | **PRKCA** | -1.889 |  |  |  |  |
|  |  | **FOXQ1** | -1.88 |  |  |  |  |
|  |  | **MAPK1** | -1.879 |  |  |  |  |
|  |  | **ITGA2** | -1.873 |  |  |  |  |
|  |  | **JUN** | -1.871 |  |  |  |  |
|  |  | **MAP3K8** | -1.871 |  |  |  |  |
|  |  | **KRAS** | -1.824 |  |  |  |  |
|  |  | **SMAD4** | -1.8 |  |  |  |  |
|  |  | **VEGFC** | -1.796 |  |  |  |  |
|  |  | **MIR21** | -1.795 |  |  |  |  |
|  |  | **ADAM10** | -1.774 |  |  |  |  |
|  |  | **ALCAM** | -1.768 |  |  |  |  |
|  |  | **ANKRD28** | -1.757 |  |  |  |  |
|  |  | **FNBP1L** | -1.753 |  |  |  |  |
|  |  | **APP** | -1.751 |  |  |  |  |
|  |  | **IQGAP1** | -1.751 |  |  |  |  |
|  |  | **WWTR1** | -1.746 |  |  |  |  |
|  |  | **PLD1** | -1.734 |  |  |  |  |
|  |  | **VAMP7** | -1.733 |  |  |  |  |
|  |  | **MAP4K4** | -1.729 |  |  |  |  |
|  |  | **FER** | -1.721 |  |  |  |  |
|  |  | **GOLPH3** | -1.717 |  |  |  |  |
|  |  | **CD40** | -1.717 |  |  |  |  |
|  |  | **PTGER4** | -1.712 |  |  |  |  |
|  |  | **TRIO** | -1.712 |  |  |  |  |
|  |  | **USP9X** | -1.709 |  |  |  |  |
|  |  | **F2R** | -1.706 |  |  |  |  |
|  |  | **GNAI3** | -1.699 |  |  |  |  |
|  |  | **HS.4988** | -1.698 |  |  |  |  |
|  |  | **AURKA** | -1.697 |  |  |  |  |
|  |  | **ERBB2** | -1.683 |  |  |  |  |
|  |  | **CDKN1A** | -1.678 |  |  |  |  |
|  |  | **ZEB1** | -1.67 |  |  |  |  |
|  |  | **HAS2** | -1.67 |  |  |  |  |
|  |  | **EGFR** | -1.661 |  |  |  |  |
|  |  | **ITPR1** | -1.653 |  |  |  |  |
|  |  | **RHOB** | -1.651 |  |  |  |  |
|  |  | **WASL** | -1.648 |  |  |  |  |
|  |  | **PAK2** | -1.645 |  |  |  |  |
|  |  | **RANBP9** | -1.641 |  |  |  |  |
|  |  | **MTDH** | -1.623 |  |  |  |  |
|  |  | **ANXA2** | -1.619 |  |  |  |  |
|  |  | **LMO7** | -1.61 |  |  |  |  |
|  |  | **FYN** | -1.601 |  |  |  |  |
|  |  | **ETV4** | -1.6 |  |  |  |  |
|  |  | **ECT2** | -1.593 |  |  |  |  |
|  |  | **CAPN2** | -1.585 |  |  |  |  |
|  |  | **LTBP2** | -1.58 |  |  |  |  |
|  |  | **SLC2A1** | -1.571 |  |  |  |  |
|  |  | **JAK2** | -1.568 |  |  |  |  |
|  |  | **CSE1L** | -1.566 |  |  |  |  |
|  |  | **MUC1** | -1.556 |  |  |  |  |
|  |  | **AKAP11** | -1.551 |  |  |  |  |
|  |  | **HGF** | -1.545 |  |  |  |  |
|  |  | **CTNNAL1** | -1.526 |  |  |  |  |
|  |  | **TCF12** | -1.522 |  |  |  |  |
|  |  | **ARRB2** | -1.519 |  |  |  |  |
|  |  | **FRAP1** | -1.518 |  |  |  |  |
|  |  | **CXCR4** | -1.515 |  |  |  |  |
|  |  | **ACSL4** | -1.515 |  |  |  |  |
|  |  | **CTBP2** | -1.508 |  |  |  |  |
|  |  | **WASF1** | -1.507 |  |  |  |  |
|  |  | **DNM2** | -1.506 |  |  |  |  |
|  |  | **EZH2** | -1.503 |  |  |  |  |
