# Supplementary material for: Specificity protein (Sp) transcription factors Sp1, Sp3 and Sp4 are non-oncogene addiction genes in cancer cells
Source: Oncotarget. 2016 Mar 5;7(16):22245–56. doi: 10.18632/oncotarget.7925 (PMC5008359; doi:10.18632/oncotarget.7925)
Supplement: Supplementary file 4 [file oncotarget-07-22245-s004.docx]

Supplemental Table S1. Sp1-regulated associated with growth inhibition, cell death and inhibition of migration/invasion after Sp1 knockdown: expected and inversely regulated genes.

A.

| **Expected** | | | | **Inversely related** | | | |
| --- | --- | --- | --- | --- | --- | --- | --- |
| **decreased cell proliferation** | | | | **increased cell proliferation** | | | |
| **up-genes (118)** | fold change | **down genes (241)** | fold change | **up-genes (150)** | fold change | **down genes (120)** | fold change |
| **IFITM1** | 12.322 | **RRM2** | -7.791 | **LMNA** | 9.997 | **CASP3** | -3.784 |
| **HMOX1** | 7.399 | **VIM** | -5.703 | **ISG15** | 5.775 | **NFIB** | -3.751 |
| **CEBPD** | 5.371 | **DKK1** | -4.643 | **C1QBP** | 4.647 | **SIRT2** | -3.049 |
| **SOD2** | 5.022 | **EGR1** | -4.506 | **CXCL2** | 4.516 | **SPRY1** | -2.903 |
| **OAS3** | 4.22 | **FOS** | -4.319 | **CBX7** | 4.368 | **TCF12** | -2.839 |
| **IFNL1** | 4.039 | **PKM** | -4.043 | **CX3CL1** | 4.048 | **ARRDC3** | -2.72 |
| **RNH1** | 4.007 | **CCNB1** | -3.893 | **ISG20** | 3.98 | **NFIX** | -2.667 |
| **MSX1** | 3.958 | **CD44** | -3.853 | **CCL5** | 3.871 | **RND3** | -2.651 |
| **GEMIN2** | 3.761 | **BMI1** | -3.772 | **BATF2** | 3.571 | **ABCC5** | -2.643 |
| **IFIT3** | 3.75 | **DUSP6** | -3.68 | **ETS1** | 3.545 | **TJP1** | -2.634 |
| **IL32** | 3.678 | **CDK19** | -3.586 | **RCE1** | 3.201 | **KLK3** | -2.632 |
| **ABCC3** | 3.603 | **CITED2** | -3.579 | **SET** | 3.167 | **EIF4G2** | -2.619 |
| **TIMP2** | 3.59 | **PDE5A** | -3.301 | **TNF** | 3.013 | **TRIB1** | -2.587 |
| **NPTX1** | 3.572 | **CENPF** | -3.165 | **NOTCH4** | 2.904 | **PSRC1** | -2.542 |
| **CXCL10** | 3.57 | **KIF20A** | -3.119 | **NOLC1** | 2.76 | **AKAP12** | -2.536 |
| **SERPINA3** | 3.451 | **PXN** | -3.096 | **SRPK2** | 2.753 | **NRP1** | -2.48 |
| **CD83** | 3.429 | **CCND3** | -2.962 | **UBD** | 2.732 | **SLC9A3R1** | -2.433 |
| **TNFAIP3** | 3.066 | **KIF11** | -2.927 | **NDEL1** | 2.707 | **LIN9** | -2.367 |
| **CDCA4** | 2.909 | **ASPM** | -2.887 | **SERPINH1** | 2.695 | **KIF23** | -2.156 |
| **PTX3** | 2.81 | **TOP2A** | -2.876 | **EBI3** | 2.681 | **FRMD6** | -2.094 |
| **GPR56** | 2.782 | **DDAH1** | -2.847 | **CCL2** | 2.649 | **SPRY2** | -2.073 |
| **DACT3** | 2.685 | **EOMES** | -2.712 | **ANTXR1** | 2.64 | **KIF2C** | -2.066 |
| **GBP1** | 2.664 | **SS18** | -2.698 | **RAC2** | 2.57 | **PLOD2** | -2.063 |
| **LEPROT** | 2.637 | **MAP3K7** | -2.693 | **LRPAP1** | 2.531 | **CDKN3** | -2.053 |
| **COMMD5** | 2.596 | **EGFR** | -2.562 | **STAT5A** | 2.468 | **ASPH** | -2.036 |
| **CBL** | 2.56 | **RAP1B** | -2.559 | **TPD52** | 2.428 | **SRGAP2** | -1.99 |
| **STC1** | 2.49 | **SLC2A1** | -2.53 | **UPP1** | 2.424 | **SESN1** | -1.986 |
| **EIF1** | 2.471 | **BIRC5** | -2.525 | **S100A4** | 2.414 | **TGFBR2** | -1.966 |
| **TAP1** | 2.422 | **CCNA2** | -2.515 | **DIXDC1** | 2.407 | **WASPIP** | -1.943 |
| **IFNL2** | 2.42 | **PRKCA** | -2.515 | **PTPMT1** | 2.395 | **CBFB** | -1.929 |
| **PARP10** | 2.395 | **HIF1A** | -2.482 | **CYP1B1** | 2.32 | **HS.130036** | -1.927 |
| **PHLPP2** | 2.366 | **BUB1** | -2.457 | **CBS** | 2.316 | **RBL2** | -1.897 |
| **MUL1** | 2.343 | **HMMR** | -2.446 | **EMP1** | 2.271 | **PHC1** | -1.894 |
| **IRF1** | 2.31 | **USP47** | -2.431 | **SDC4** | 2.252 | **MIB1** | -1.861 |
| **HRASLS** | 2.294 | **DLGAP5** | -2.398 | **PTP4A3** | 2.247 | **CHES1** | -1.85 |
| **BECN1** | 2.285 | **IRS1** | -2.37 | **SHC1** | 2.232 | **HIPK2** | -1.85 |
| **RALBP1** | 2.277 | **AURKA** | -2.368 | **MVP** | 2.184 | **EGR2** | -1.842 |
| **PLSCR1** | 2.267 | **PAPSS2** | -2.334 | **BCL2L1** | 2.147 | **APBB2** | -1.837 |
| **RARRES3** | 2.248 | **KIF20B** | -2.318 | **NAP1L1** | 2.135 | **TFAM** | -1.823 |
| **TNFRSF9** | 2.211 | **DICER1** | -2.301 | **CTSD** | 2.132 | **GJA1** | -1.821 |
| **UCP2** | 2.179 | **NEK2** | -2.297 | **SUMO2** | 2.117 | **CCNG2** | -1.787 |
| **GAL** | 2.172 | **CYR61** | -2.284 | **MGAT3** | 2.108 | **RB1** | -1.786 |
| **TNFRSF14** | 2.162 | **MAPK6** | -2.25 | **YWHAG** | 2.096 | **TP53BP2** | -1.776 |
| **SIGIRR** | 2.162 | **AGO2** | -2.238 | **CCRK** | 2.095 | **TFPI** | -1.762 |
| **SOCS1** | 2.153 | **CDCA8** | -2.234 | **CD320** | 2.078 | **ITGA6** | -1.757 |
| **EI24** | 2.151 | **PPIA** | -2.23 | **EWSR1** | 2.031 | **ERRFI1** | -1.747 |
| **DAXX** | 2.144 | **TESK1** | -2.215 | **FSTL3** | 2.028 | **WNK2** | -1.738 |
| **PMP22** | 2.12 | **STAT5B** | -2.211 | **SRF** | 2.018 | **CAT** | -1.729 |
| **RASSF5** | 2.108 | **P4HA1** | -2.201 | **TBP** | 2.002 | **TRIM24** | -1.709 |
| **CAMK2N1** | 2.052 | **SPTBN1** | -2.188 | **ARHGEF2** | 1.991 | **ATG5** | -1.701 |
| **MAFG** | 2.038 | **CDK1** | -2.184 | **CORO1B** | 1.99 | **PKP2** | -1.697 |
| **GNE** | 2.014 | **TPX2** | -2.147 | **FEZ1** | 1.98 | **FOXF2** | -1.693 |
| **DNAJA3** | 2.011 | **ERCC1** | -2.115 | **NCOR2** | 1.973 | **RASA1** | -1.692 |
| **SMPD1** | 2.009 | **PBK** | -2.106 | **RALGDS** | 1.953 | **FBXO11** | -1.686 |
| **IFI16** | 2.008 | **RPRD1B** | -2.098 | **CCL20** | 1.95 | **SPRED1** | -1.685 |
| **SMARCA2** | 1.994 | **TTK** | -2.084 | **GBP2** | 1.947 | **HNRNPA1** | -1.675 |
| **TNFRSF1B** | 1.973 | **YBX1** | -2.083 | **IGF2BP3** | 1.946 | **USP10** | -1.672 |
| **LRRC32** | 1.969 | **RGS2** | -2.075 | **MAP1B** | 1.943 | **NDFIP1** | -1.67 |
| **IFITM3** | 1.962 | **PLXNB2** | -2.066 | **BCL3** | 1.938 | **KLHL13** | -1.667 |
| **TRAF1** | 1.959 | **LRP5** | -2.036 | **PSMA4** | 1.934 | **B3GNT2** | -1.666 |
| **MED25** | 1.934 | **TACC3** | -2.036 | **MYD88** | 1.924 | **HS.371609** | -1.659 |
| **IFNL3** | 1.923 | **RAB22A** | -2.018 | **SLC7A5** | 1.913 | **RAD17** | -1.653 |
| **NDRG4** | 1.898 | **EGLN1** | -2.008 | **CDK4** | 1.911 | **MAP2K1IP1** | -1.649 |
| **NUMA1** | 1.893 | **ECT2** | -1.989 | **STMN3** | 1.908 | **ENPP1** | -1.642 |
| **RPS6KA2** | 1.889 | **PIK3R1** | -1.986 | **ARF1** | 1.903 | **G3BP1** | -1.64 |
| **FBXO2** | 1.847 | **EPAS1** | -1.985 | **ATP5G1** | 1.903 | **TOP1** | -1.637 |
| **DIABLO** | 1.843 | **FAT4** | -1.983 | **ST5** | 1.898 | **STARD13** | -1.621 |
| **TRIM22** | 1.835 | **PPP1R13L** | -1.979 | **ID3** | 1.898 | **RAD21** | -1.618 |
| **NACC2** | 1.818 | **KIF18A** | -1.976 | **STX3** | 1.895 | **CTBP2** | -1.618 |
| **IGFBP6** | 1.813 | **NFIA** | -1.971 | **PRNP** | 1.857 | **FOXA2** | -1.607 |
| **EFNB3** | 1.812 | **CDC20** | -1.966 | **DCLRE1A** | 1.85 | **CMA1** | -1.602 |
| **DKK3** | 1.805 | **TRIB2** | -1.943 | **JAG1** | 1.837 | **NAB1** | -1.601 |
| **PCBP4** | 1.79 | **TTLL4** | -1.935 | **HMGB1** | 1.811 | **TES** | -1.594 |
| **QPCT** | 1.77 | **NUDT1** | -1.917 | **NACA** | 1.806 | **RANBP9** | -1.591 |
| **GADD45A** | 1.755 | **CTGF** | -1.906 | **NOD2** | 1.794 | **PLXNA3** | -1.583 |
| **NKX3-1** | 1.753 | **NRARP** | -1.9 | **ICAM1** | 1.793 | **RTN4** | -1.583 |
| **EPHA2** | 1.749 | **TRPC1** | -1.889 | **ABCG1** | 1.77 | **CASP7** | -1.582 |
| **PMAIP1** | 1.745 | **LBR** | -1.886 | **TAGLN2** | 1.769 | **MIR1-2** | -1.58 |
| **GDF15** | 1.736 | **RPS6KB1** | -1.872 | **LCN2** | 1.764 | **SMPD2** | -1.573 |
| **LIMK1** | 1.7 | **CSNK1G3** | -1.862 | **NCOA4** | 1.763 | **H2AFY** | -1.57 |
| **CCL3L3** | 1.698 | **PTK2** | -1.858 | **ALG13** | 1.754 | **PMEPA1** | -1.567 |
| **STAT2** | 1.692 | **PIP4K2A** | -1.856 | **LAP3** | 1.742 | **KLF10** | -1.566 |
| **NDUFS3** | 1.675 | **CCDC88A** | -1.855 | **LIF** | 1.736 | **CHEK2** | -1.562 |
| **SREBF1** | 1.672 | **UIMC1** | -1.854 | **ICOSLG** | 1.731 | **SEMA3A** | -1.561 |
| **ATF3** | 1.666 | **LAMC1** | -1.854 | **CSPG5** | 1.73 | **DAB2** | -1.559 |
| **B2M** | 1.665 | **TAB2** | -1.852 | **GCLC** | 1.728 | **SUV39H1** | -1.558 |
| **RAP1GAP** | 1.64 | **MBP** | -1.848 | **BAMBI** | 1.726 | **CTCF** | -1.557 |
| **ACTN4** | 1.638 | **HGF** | -1.847 | **PRMT5** | 1.722 | **SIAH1** | -1.554 |
| **PHF14** | 1.626 | **ATMIN** | -1.845 | **CDK6** | 1.714 | **COL4A1** | -1.553 |
| **LPIN1** | 1.619 | **FRS2** | -1.838 | **NFKB2** | 1.707 | **MERTK** | -1.553 |
| **BST2** | 1.612 | **ERO1LB** | -1.824 | **SKP2** | 1.706 | **SEC23A** | -1.553 |
| **IRX3** | 1.61 | **TGIF1** | -1.822 | **PSMB10** | 1.705 | **MIR203** | -1.552 |
| **INCA1** | 1.609 | **FBXW11** | -1.808 | **GPC1** | 1.695 | **ADORA3** | -1.55 |
| **TSC1** | 1.606 | **RACGAP1** | -1.806 | **ATM** | 1.695 | **MIR138-1** | -1.549 |
| **TP53I11** | 1.593 | **ZIC2** | -1.803 | **PPL** | 1.688 | **EMX2** | -1.541 |
| **ARHGDIB** | 1.58 | **CDCA2** | -1.803 | **IL15** | 1.681 | **APRIN** | -1.54 |
| **RHOB** | 1.579 | **GBF1** | -1.793 | **XBP1** | 1.681 | **TNFRSF8** | -1.538 |
| **JUND** | 1.579 | **ANXA2** | -1.791 | **MNAT1** | 1.679 | **CXADR** | -1.537 |
| **ZMPSTE24** | 1.569 | **ITGA2** | -1.789 | **NP** | 1.677 | **CNKSR1** | -1.535 |
| **SIRPA** | 1.566 | **GNAI2** | -1.788 | **PRKAR2A** | 1.672 | **PATZ1** | -1.533 |
| **NFKBIB** | 1.563 | **FOXQ1** | -1.783 | **CDC25B** | 1.67 | **P4HA2** | -1.533 |
| **LITAF** | 1.55 | **PTGER4** | -1.781 | **CXCL1** | 1.667 | **FAM188A** | -1.529 |
| **STAT1** | 1.549 | **NFYA** | -1.777 | **CISD1** | 1.666 | **CASK** | -1.526 |
| **BAG6** | 1.548 | **NBN** | -1.766 | **PLAT** | 1.664 | **TSPYL2** | -1.525 |
| **EEF1E1** | 1.546 | **STAG1** | -1.763 | **SLC25A4** | 1.658 | **MTBP** | -1.524 |
| **CEACAM1** | 1.545 | **ZMYM2** | -1.762 | **SNX12** | 1.651 | **SPRY4** | -1.519 |
| **UXT** | 1.544 | **BLM** | -1.76 | **MED28** | 1.646 | **CAST** | -1.518 |
| **NUP62** | 1.54 | **HAS2** | -1.758 | **SPTAN1** | 1.644 | **RUNX1** | -1.518 |
| **TSC22D3** | 1.539 | **KPNA2** | -1.757 | **MST1** | 1.638 | **BMP5** | -1.517 |
| **HSPA1A/HSPA1B** | 1.527 | **PTP4A2** | -1.746 | **GPR177** | 1.636 | **ADRA1A** | -1.516 |
| **NUPR1** | 1.527 | **PTPN3** | -1.744 | **SRC** | 1.634 | **CD55** | -1.514 |
| **E2F7** | 1.524 | **DVL3** | -1.744 | **ESRRA** | 1.621 | **KRT7** | -1.512 |
| **SAT1** | 1.522 | **RNF126** | -1.733 | **DPYSL2** | 1.618 | **DUX4** | -1.512 |
| **BIN1** | 1.521 | **SUV420H1** | -1.713 | **ZFP36L1** | 1.617 | **IL1RN** | -1.51 |
| **PHB** | 1.515 | **NCAPG** | -1.71 | **WDR12** | 1.613 | **CAPRIN1** | -1.509 |
| **LRIG1** | 1.515 | **PRPS2** | -1.705 | **NFKB1** | 1.611 | **ARID4A** | -1.504 |
| **NAIF1** | 1.511 | **H2AFX** | -1.703 | **IL15RA** | 1.606 | **CNN2** | -1.502 |
| **AGTRAP** | 1.508 | **PYCARD** | -1.702 | **DYRK1B** | 1.605 | **BACH2** | -1.501 |
|  |  | **MAD2L1** | -1.7 | **NOP58** | 1.605 | **CNTN1** | -1.5 |
|  |  | **MKI67** | -1.7 | **PDCL3** | 1.6 | **PTPN2** | -1.5 |
|  |  | **NRG1** | -1.697 | **TFE3** | 1.593 |  |  |
|  |  | **INO80** | -1.694 | **CNPY2** | 1.59 |  |  |
|  |  | **ELMO2** | -1.693 | **USP18** | 1.59 |  |  |
|  |  | **TYMS** | -1.692 | **NFKBIA** | 1.588 |  |  |
|  |  | **ABL1** | -1.689 | **TXLNA** | 1.588 |  |  |
|  |  | **FOXM1** | -1.688 | **RAB33A** | 1.583 |  |  |
|  |  | **POLR3E** | -1.687 | **DUSP12** | 1.577 |  |  |
|  |  | **NFAT5** | -1.686 | **TSPAN3** | 1.577 |  |  |
|  |  | **RAN** | -1.683 | **MYH14** | 1.562 |  |  |
|  |  | **NCOA2** | -1.683 | **POLR2L** | 1.56 |  |  |
|  |  | **NDRG1** | -1.68 | **PDCD10** | 1.558 |  |  |
|  |  | **KCNMA1** | -1.677 | **PPP1R15A** | 1.557 |  |  |
|  |  | **YBX3** | -1.676 | **ALS2** | 1.556 |  |  |
|  |  | **FGFR4** | -1.675 | **TNFSF10** | 1.555 |  |  |
|  |  | **NFS1** | -1.674 | **CD58** | 1.546 |  |  |
|  |  | **HGS** | -1.672 | **TNFSF9** | 1.545 |  |  |
|  |  | **AGO4** | -1.67 | **COPS2** | 1.534 |  |  |
|  |  | **BCAR3** | -1.666 | **TNFAIP8** | 1.529 |  |  |
|  |  | **PLAG1** | -1.666 | **SLC3A2** | 1.529 |  |  |
|  |  | **NASP** | -1.661 | **TOP1MT** | 1.525 |  |  |
|  |  | **GAD1** | -1.659 | **VEGFB** | 1.519 |  |  |
|  |  | **MALT1** | -1.656 | **VGF** | 1.517 |  |  |
|  |  | **DOCK1** | -1.656 | **TRNP1** | 1.517 |  |  |
|  |  | **PAK2** | -1.654 | **RNASEH2B** | 1.516 |  |  |
|  |  | **DVL2** | -1.653 | **BCL2A1** | 1.515 |  |  |
|  |  | **AKR1C1/AKR1C2** | -1.652 | **CLN3** | 1.514 |  |  |
|  |  | **ATAD2** | -1.651 | **CDC42** | 1.512 |  |  |
|  |  | **PRKAA1** | -1.651 | **AKIRIN2** | 1.508 |  |  |
|  |  | **KMT2C** | -1.651 | **RCAN1** | 1.506 |  |  |
|  |  | **ACVR2A** | -1.648 | **CDK5R1** | 1.502 |  |  |
|  |  | **JAK2** | -1.642 |  |  |  |  |
|  |  | **PDK1** | -1.638 |  |  |  |  |
|  |  | **ROR1** | -1.638 |  |  |  |  |
|  |  | **TXNDC5** | -1.637 |  |  |  |  |
|  |  | **YAP1** | -1.632 |  |  |  |  |
|  |  | **F2RL1** | -1.631 |  |  |  |  |
|  |  | **NR2C2** | -1.63 |  |  |  |  |
|  |  | **LSM1** | -1.63 |  |  |  |  |
|  |  | **GPN3** | -1.629 |  |  |  |  |
|  |  | **USP7** | -1.627 |  |  |  |  |
|  |  | **CDC45** | -1.619 |  |  |  |  |
|  |  | **SLC29A1** | -1.618 |  |  |  |  |
|  |  | **PAK4** | -1.617 |  |  |  |  |
|  |  | **ARFGAP3** | -1.603 |  |  |  |  |
|  |  | **BUB3** | -1.602 |  |  |  |  |
|  |  | **MAPK10** | -1.602 |  |  |  |  |
|  |  | **DIDO1** | -1.601 |  |  |  |  |
|  |  | **LDHA** | -1.6 |  |  |  |  |
|  |  | **PTPN13** | -1.6 |  |  |  |  |
|  |  | **F2R** | -1.599 |  |  |  |  |
|  |  | **PTTG1** | -1.595 |  |  |  |  |
|  |  | **ACTB** | -1.593 |  |  |  |  |
|  |  | **AURKB** | -1.593 |  |  |  |  |
|  |  | **KIF2A** | -1.591 |  |  |  |  |
|  |  | **MSRA** | -1.59 |  |  |  |  |
|  |  | **CAMK4** | -1.589 |  |  |  |  |
|  |  | **WWTR1** | -1.588 |  |  |  |  |
|  |  | **MCM4** | -1.587 |  |  |  |  |
|  |  | **LDLRAP1** | -1.586 |  |  |  |  |
|  |  | **PLK1** | -1.583 |  |  |  |  |
|  |  | **BAIAP2L1** | -1.583 |  |  |  |  |
|  |  | **DHFR** | -1.581 |  |  |  |  |
|  |  | **AKT2** | -1.579 |  |  |  |  |
|  |  | **NEDD4L** | -1.578 |  |  |  |  |
|  |  | **TEAD4** | -1.577 |  |  |  |  |
|  |  | **B4GALT6** | -1.575 |  |  |  |  |
|  |  | **KRAS** | -1.573 |  |  |  |  |
|  |  | **AR** | -1.57 |  |  |  |  |
|  |  | **TBX1** | -1.567 |  |  |  |  |
|  |  | **PDGFC** | -1.566 |  |  |  |  |
|  |  | **SERPINB9** | -1.566 |  |  |  |  |
|  |  | **PCGF2** | -1.561 |  |  |  |  |
|  |  | **IL13RA1** | -1.561 |  |  |  |  |
|  |  | **ITGB1** | -1.558 |  |  |  |  |
|  |  | **LIN28B** | -1.551 |  |  |  |  |
|  |  | **NTS** | -1.549 |  |  |  |  |
|  |  | **CHN1** | -1.545 |  |  |  |  |
|  |  | **POLH** | -1.543 |  |  |  |  |
|  |  | **KDM5B** | -1.541 |  |  |  |  |
|  |  | **E2F8** | -1.54 |  |  |  |  |
|  |  | **IL2RG** | -1.539 |  |  |  |  |
|  |  | **AGO3** | -1.538 |  |  |  |  |
|  |  | **TP63** | -1.537 |  |  |  |  |
|  |  | **CREB1** | -1.536 |  |  |  |  |
|  |  | **ID4** | -1.534 |  |  |  |  |
|  |  | **LCK** | -1.534 |  |  |  |  |
|  |  | **CIZ1** | -1.534 |  |  |  |  |
|  |  | **SMN1/SMN2** | -1.533 |  |  |  |  |
|  |  | **CTTN** | -1.532 |  |  |  |  |
|  |  | **SH3KBP1** | -1.531 |  |  |  |  |
|  |  | **EPS8** | -1.53 |  |  |  |  |
|  |  | **MCM10** | -1.53 |  |  |  |  |
|  |  | **MCL1** | -1.529 |  |  |  |  |
|  |  | **AKT3** | -1.527 |  |  |  |  |
|  |  | **AKIRIN1** | -1.525 |  |  |  |  |
|  |  | **CAMKK1** | -1.524 |  |  |  |  |
|  |  | **AFAP1** | -1.522 |  |  |  |  |
|  |  | **TCF4** | -1.521 |  |  |  |  |
|  |  | **DDX17** | -1.52 |  |  |  |  |
|  |  | **RTKN2** | -1.519 |  |  |  |  |
|  |  | **HOXB3** | -1.518 |  |  |  |  |
|  |  | **SGK1** | -1.516 |  |  |  |  |
|  |  | **MEF2C** | -1.515 |  |  |  |  |
|  |  | **CDC16** | -1.514 |  |  |  |  |
|  |  | **FGF10** | -1.514 |  |  |  |  |
|  |  | **ABCC9** | -1.513 |  |  |  |  |
|  |  | **CDT1** | -1.513 |  |  |  |  |
|  |  | **CALB1** | -1.51 |  |  |  |  |
|  |  | **RIPK1** | -1.509 |  |  |  |  |
|  |  | **PKP3** | -1.509 |  |  |  |  |
|  |  | **PIK3R3** | -1.508 |  |  |  |  |
|  |  | **ACSL4** | -1.508 |  |  |  |  |
|  |  | **UBE2C** | -1.508 |  |  |  |  |
|  |  | **NUP98** | -1.508 |  |  |  |  |
|  |  | **mir-154** | -1.507 |  |  |  |  |
|  |  | **VASP** | -1.507 |  |  |  |  |
|  |  | **RAF1** | -1.504 |  |  |  |  |
|  |  | **VEGFC** | -1.503 |  |  |  |  |
|  |  | **ST6GAL1** | -1.502 |  |  |  |  |
|  |  | **F11R** | -1.501 |  |  |  |  |
|  |  | **CHEK1** | -1.5 |  |  |  |  |

B.

| **Expected** | | | | **Inversely related** | | | |
| --- | --- | --- | --- | --- | --- | --- | --- |
| **Increased cell death** | | | | **Decreased cell death** | | | |
| **Up-genes**  **(140)** | Fold change | **Down genes**  **(223)** | Fold change | **Up-genes**  **(149)**  **90** | Fold change | **Down genes**  **(141)** | Fold change |
| **ISG15** | 5.775 | **RRM2** | -7.791 | **HMOX1** | 7.399 | **NME4** | -5.372 |
| **OAS1** | 5.763 | **STK40** | -4.272 | **IFI6** | 5.463 | **DKK1** | -4.643 |
| **CEBPD** | 5.371 | **PKM2** | -4.043 | **SOD2** | 5.022 | **EGR1** | -4.506 |
| **MX1** | 4.743 | **SLC4A7** | -3.856 | **CXCL2** | 4.516 | **FOS** | -4.319 |
| **OAS3** | 4.220 | **CD44** | -3.853 | **LGALS3BP** | 4.241 | **CCNB1** | -3.893 |
| **CX3CL1** | 4.048 | **BMI1** | -3.772 | **IL29** | 4.039 | **CASP3** | -3.784 |
| **NPTX1** | 3.572 | **NFIB** | -3.751 | **MSX1** | 3.958 | **DUSP6** | -3.680 |
| **CXCL10** | 3.570 | **CITED2** | -3.579 | **STX8** | 3.903 | **PDE5A** | -3.301 |
| **ETS1** | 3.545 | **DEK** | -3.429 | **CCL5** | 3.871 | **SIRT2** | -3.049 |
| **HS1BP3** | 3.536 | **PCNA** | -3.224 | **SIP1** | 3.761 | **DNASE1L1** | -3.038 |
| **IFIH1** | 3.332 | **CENPF** | -3.165 | **IFIT3** | 3.750 | **TOP2A** | -2.876 |
| **SMARCC1** | 3.297 | **PXN** | -3.096 | **IL32** | 3.678 | **TCF12** | -2.839 |
| **SET** | 3.167 | **CCND3** | -2.962 | **ABCC3** | 3.603 | **TFDP2** | -2.646 |
| **TNF** | 3.013 | **KIF11** | -2.927 | **TIMP2** | 3.590 | **APOBEC3B** | -2.518 |
| **NOTCH4** | 2.904 | **EOMES** | -2.712 | **SERPINA3** | 3.451 | **HMMR** | -2.446 |
| **NFKBIZ** | 2.775 | **MAP3K7** | -2.693 | **SELE** | 3.414 | **SPAG5** | -2.398 |
| **SRPK2** | 2.753 | **NFIX** | -2.667 | **TUB** | 3.155 | **ANTXR2** | -2.383 |
| **UBD** | 2.732 | **RND3** | -2.651 | **TNFAIP3** | 3.066 | **SLC25A24** | -2.320 |
| **BOK** | 2.709 | **ABCC5** | -2.643 | **HSPBP1** | 2.927 | **ZFYVE16** | -2.307 |
| **CASP4** | 2.672 | **KLK3** | -2.632 | **DAG1** | 2.771 | **ZAK** | -2.283 |
| **ANTXR1** | 2.640 | **EIF4G2** | -2.619 | **NOLC1** | 2.760 | **CCDC109A** | -2.260 |
| **ADORA2A** | 2.600 | **TRIB1** | -2.587 | **NDEL1** | 2.707 | **ATP2A2** | -2.192 |
| **RAC2** | 2.570 | **EGFR** | -2.562 | **HAND1** | 2.707 | **SPTBN1** | -2.188 |
| **STC1** | 2.490 | **RAP1B** | -2.559 | **SERPINH1** | 2.695 | **CDC2** | -2.184 |
| **ZC3H12A** | 2.471 | **PON2** | -2.548 | **CCL2** | 2.649 | **UACA** | -2.156 |
| **ITGB3BP** | 2.384 | **AKAP12** | -2.536 | **CBL** | 2.560 | **FRMD6** | -2.094 |
| **CYP1B1** | 2.320 | **SLC2A1** | -2.530 | **LRPAP1** | 2.531 | **GULP1** | -2.088 |
| **IRF1** | 2.310 | **BIRC5** | -2.525 | **PCTP** | 2.523 | **TTK** | -2.084 |
| **HRASLS** | 2.294 | **PRKCA** | -2.515 | **STAT5A** | 2.468 | **CKAP2** | -2.054 |
| **BECN1** | 2.285 | **HIF1A** | -2.482 | **TRIAP1** | 2.438 | **CDKN3** | -2.053 |
| **SP110** | 2.277 | **NRP1** | -2.480 | **TPD52** | 2.428 | **LRP5** | -2.036 |
| **EMP1** | 2.271 | **USP47** | -2.431 | **IL28A** | 2.420 | **ELF4** | -2.022 |
| **PLSCR1** | 2.267 | **NEK6** | -2.426 | **S100A4** | 2.414 | **RAB22A** | -2.018 |
| **PI3** | 2.266 | **NDC80** | -2.415 | **PSMB8** | 2.407 | **FAF1** | -1.993 |
| **MYH9** | 2.255 | **KIF14** | -2.384 | **PTPMT1** | 2.395 | **ECT2** | -1.989 |
| **SDC4** | 2.252 | **IRS1** | -2.370 | **ACO2** | 2.371 | **SNAP25** | -1.980 |
| **SHC1** | 2.232 | **AURKA** | -2.368 | **LMO4** | 2.318 | **CDC20** | -1.966 |
| **TNFRSF9** | 2.211 | **SOX21** | -2.322 | **CBS** | 2.316 | **WASPIP** | -1.943 |
| **MVP** | 2.184 | **DICER1** | -2.301 | **GBA** | 2.230 | **TRIB2** | -1.943 |
| **DAXX** | 2.144 | **NEK2** | -2.297 | **GAL** | 2.172 | **TRAF7** | -1.941 |
| **IFIT2** | 2.121 | **CYR61** | -2.284 | **SIGIRR** | 2.162 | **HS.130036** | -1.927 |
| **PMP22** | 2.120 | **EIF2C2** | -2.238 | **SOCS1** | 2.153 | **ST3GAL3** | -1.914 |
| **RNF13** | 2.118 | **PPIA** | -2.230 | **EI24** | 2.151 | **TRPS1** | -1.913 |
| **FSTL3** | 2.028 | **STAT5B** | -2.211 | **BCL2L1** | 2.147 | **FBXO32** | -1.910 |
| **DNAJA3** | 2.011 | **CENPE** | -2.197 | **WFS1** | 2.137 | **SCP2** | -1.900 |
| **SMPD1** | 2.009 | **CCT3** | -2.181 | **MGAT3** | 2.108 | **FAM72A** | -1.875 |
| **IFI16** | 2.008 | **TUBB3** | -2.150 | **YWHAG** | 2.096 | **BUB1B** | -1.867 |
| **PRELID1** | 2.005 | **TPX2** | -2.147 | **EBAG9** | 2.091 | **MIB1** | -1.861 |
| **SMARCA2** | 1.994 | **DHRS2** | -2.132 | **ECGF1** | 2.090 | **CCDC88A** | -1.855 |
| **LAMP1** | 1.975 | **NT5E** | -2.120 | **OSCAR** | 2.088 | **HIPK2** | -1.850 |
| **TNFRSF1B** | 1.973 | **SRI** | -2.120 | **BEX2** | 2.082 | **MBP** | -1.848 |
| **NCOR2** | 1.973 | **ERCC1** | -2.115 | **PINK1** | 2.062 | **XPR1** | -1.842 |
| **MAP3K8** | 1.958 | **PBK** | -2.106 | **WDR4** | 2.045 | **RFK** | -1.832 |
| **MAP1B** | 1.943 | **SORBS2** | -2.105 | **MAFG** | 2.038 | **TIA1** | -1.822 |
| **XAF1** | 1.940 | **CCT5** | -2.088 | **EWSR1** | 2.031 | **CDC25C** | -1.810 |
| **MYD88** | 1.924 | **YBX1** | -2.083 | **MAD2L2** | 2.028 | **CABLES2** | -1.799 |
| **CDK4** | 1.911 | **SPRY2** | -2.073 | **RGS10** | 2.022 | **MAP3K9** | -1.785 |
| **ID3** | 1.898 | **STIL** | -2.041 | **SRF** | 2.018 | **PTGER4** | -1.781 |
| **PARP14** | 1.897 | **TACC3** | -2.036 | **TBP** | 2.002 | **TP53BP2** | -1.776 |
| **NUMA1** | 1.893 | **EGLN1** | -2.008 | **ARHGEF2** | 1.991 | **STAG1** | -1.763 |
| **RPS6KA2** | 1.889 | **HIPK3** | -1.993 | **TAOK3** | 1.978 | **TFPI** | -1.762 |
| **OGDH** | 1.888 | **PIK3R1** | -1.986 | **TRAF1** | 1.959 | **BLM** | -1.760 |
| **IRAK2** | 1.882 | **EPAS1** | -1.985 | **BCL3** | 1.938 | **ITGA6** | -1.757 |
| **PRNP** | 1.857 | **FAT4** | -1.983 | **CAMK2G** | 1.920 | **TSC22D2** | -1.750 |
| **DIABLO** | 1.843 | **MED29** | -1.983 | **PLEC1** | 1.916 | **MELK** | -1.729 |
| **PTRH2** | 1.836 | **PPP1R13L** | -1.979 | **NUAK1** | 1.890 | **MCM7** | -1.723 |
| **CANX** | 1.829 | **TGFBR2** | -1.966 | **SCYL1** | 1.861 | **CD69** | -1.707 |
| **SFT2D2** | 1.821 | **CENPA** | -1.965 | **STXBP1** | 1.850 | **H2AFX** | -1.703 |
| **NACC2** | 1.818 | **CKAP5** | -1.947 | **FBXO2** | 1.847 | **PYCARD** | -1.702 |
| **IGFBP6** | 1.813 | **CBFB** | -1.929 | **MAPKAP1** | 1.844 | **ATG5** | -1.701 |
| **HMGB1** | 1.811 | **NUDT1** | -1.917 | **GCLM** | 1.837 | **MAD2L1** | -1.700 |
| **DKK3** | 1.805 | **GPHN** | -1.909 | **JAG1** | 1.837 | **HS.551128** | -1.697 |
| **ICAM1** | 1.793 | **CTGF** | -1.906 | **BCL2L2** | 1.837 | **FOXF2** | -1.693 |
| **OLR1** | 1.786 | **RBL2** | -1.897 | **BFAR** | 1.800 | **RASA1** | -1.692 |
| **IRF7** | 1.774 | **TRPC1** | -1.889 | **MEF2D** | 1.797 | **ABL1** | -1.689 |
| **TGFBI** | 1.772 | **FBXO5** | -1.873 | **SH3GLB1** | 1.795 | **FGFR4** | -1.675 |
| **LCN2** | 1.764 | **RPS6KB1** | -1.872 | **PEA15** | 1.779 | **PPP2R5A** | -1.660 |
| **B4GALT5** | 1.760 | **PTK2** | -1.858 | **SLC40A1** | 1.779 | **HS.371609** | -1.659 |
| **GADD45A** | 1.755 | **UIMC1** | -1.854 | **ABCG1** | 1.770 | **MALT1** | -1.656 |
| **NKX3-1** | 1.753 | **MAP3K7IP2** | -1.852 | **TAGLN2** | 1.769 | **DOCK1** | -1.656 |
| **CMIP** | 1.751 | **HGF** | -1.847 | **NCOA4** | 1.763 | **PAK2** | -1.654 |
| **EPHA2** | 1.749 | **ATMIN** | -1.845 | **HLA-F** | 1.761 | **SDC1** | -1.638 |
| **EIF2S1** | 1.747 | **PIAS2** | -1.844 | **NAGLU** | 1.760 | **PDK1** | -1.638 |
| **PMAIP1** | 1.745 | **EGR2** | -1.842 | **GDF15** | 1.736 | **TOP1** | -1.637 |
| **LIF** | 1.736 | **HS.334831** | -1.838 | **ICOSLG** | 1.731 | **ATN1** | -1.632 |
| **CSTB** | 1.725 | **TFAM** | -1.823 | **GCLC** | 1.728 | **PERP** | -1.632 |
| **CDK6** | 1.714 | **CDCP1** | -1.816 | **PRMT5** | 1.722 | **F2RL1** | -1.631 |
| **SHISA5** | 1.709 | **RACGAP1** | -1.806 | **CLCN7** | 1.709 | **USP7** | -1.627 |
| **DRAM1** | 1.708 | **CDCA2** | -1.803 | **NFKB2** | 1.707 | **RAD21** | -1.618 |
| **CCL3L3** | 1.698 | **GJB2** | -1.796 | **SKP2** | 1.706 | **SLC29A1** | -1.618 |
| **GPC1** | 1.695 | **ANXA2** | -1.791 | **PSMB10** | 1.705 | **REPS2** | -1.616 |
| **ATM** | 1.695 | **ITGA2** | -1.789 | **CYB5A** | 1.699 | **TLR1** | -1.609 |
| **STAT2** | 1.692 | **RB1** | -1.786 | **HBXIP** | 1.690 | **STAP2** | -1.607 |
| **IL15** | 1.681 | **NFYA** | -1.777 | **XBP1** | 1.681 | **MAPK10** | -1.602 |
| **SREBF1** | 1.672 | **NSF** | -1.777 | **MNAT1** | 1.679 | **LDHA** | -1.600 |
| **ZNF622** | 1.668 | **TBL1XR1** | -1.771 | **NP** | 1.677 | **PTPN13** | -1.600 |
| **ATF3** | 1.666 | **NBN** | -1.766 | **RPS24** | 1.672 | **F2R** | -1.599 |
| **B2M** | 1.665 | **ZMYM2** | -1.762 | **ARID3B** | 1.668 | **PTTG1** | -1.595 |
| **PLAT** | 1.664 | **HAS2** | -1.758 | **CXCL1** | 1.667 | **RANBP9** | -1.591 |
| **RFXANK** | 1.658 | **KPNA2** | -1.757 | **BCL2L13** | 1.664 | **CAMK4** | -1.589 |
| **SLC25A4** | 1.658 | **PTP4A2** | -1.746 | **CHMP4B** | 1.660 | **RTN4** | -1.583 |
| **BRMS1** | 1.657 | **HADHA** | -1.746 | **RAB32** | 1.658 | **CASP7** | -1.582 |
| **HRASLS3** | 1.645 | **SPG7** | -1.740 | **MED28** | 1.646 | **CCBL1** | -1.581 |
| **ADI1** | 1.638 | **PSIP1** | -1.734 | **RAP1GAP** | 1.640 | **MIR1-2** | -1.580 |
| **PPP1R15B** | 1.631 | **FBXL5** | -1.733 | **MST1** | 1.638 | **SLAMF6** | -1.579 |
| **EFHC1** | 1.627 | **DMD** | -1.731 | **DDAH2** | 1.635 | **KRAS** | -1.573 |
| **MLKL** | 1.625 | **CAT** | -1.729 | **SRC** | 1.634 | **SMPD2** | -1.573 |
| **MLLT11** | 1.620 | **TOP2B** | -1.719 | **STRADB** | 1.623 | **AR** | -1.570 |
| **PRR7** | 1.617 | **SERP1** | -1.715 | **INTS1** | 1.619 | **PMEPA1** | -1.567 |
| **AKAP1** | 1.614 | **TRIM24** | -1.709 | **NFKB1** | 1.611 | **CHEK2** | -1.562 |
| **SDHC** | 1.613 | **GLUD1** | -1.705 | **IL15RA** | 1.606 | **SEMA3A** | -1.561 |
| **DUSP2** | 1.612 | **MKI67** | -1.700 | **TSC1** | 1.606 | **DAB2** | -1.559 |
| **TP53I11** | 1.593 | **TYMS** | -1.692 | **FLOT2** | 1.605 | **ITGB1** | -1.558 |
| **CNPY2** | 1.590 | **NUPL1** | -1.691 | **APH1B** | 1.604 | **SIAH1** | -1.554 |
| **CTSL1** | 1.587 | **FOXM1** | -1.688 | **DNAJA1** | 1.599 | **MIR203** | -1.552 |
| **MTCP1** | 1.583 | **NFAT5** | -1.686 | **USP18** | 1.590 | **ADORA3** | -1.550 |
| **ARHGDIB** | 1.580 | **NCOA2** | -1.683 | **DDIT4** | 1.589 | **MIR138-1** | -1.549 |
| **RHOB** | 1.579 | **NDRG1** | -1.680 | **NFKBIA** | 1.588 | **CAPN10** | -1.545 |
| **DEDD2** | 1.569 | **KCNMA1** | -1.677 | **JUND** | 1.579 | **POLH** | -1.543 |
| **SIRPA** | 1.566 | **CSDA** | -1.676 | **SNCB** | 1.568 | **IL2RG** | -1.539 |
| **NFKBIB** | 1.563 | **CCT6A** | -1.676 | **UBQLN1** | 1.560 | **TNFRSF8** | -1.538 |
| **RBM43** | 1.558 | **PLK4** | -1.674 | **PDCD10** | 1.558 | **DEPDC1** | -1.537 |
| **PPP1R15A** | 1.557 | **USP10** | -1.672 | **ALS2** | 1.556 | **ZMYND11** | -1.536 |
| **TNFSF10** | 1.555 | **EIF2C4** | -1.670 | **WDR68** | 1.555 | **CNKSR1** | -1.535 |
| **STAT1** | 1.549 | **TNFRSF10D** | -1.659 | **IFNGR2** | 1.554 | **RRM1** | -1.534 |
| **BAT3** | 1.548 | **DPP8** | -1.656 | **ATP5S** | 1.553 | **SH2D4A** | -1.534 |
| **EEF1E1** | 1.546 | **SPC25** | -1.655 | **CEACAM1** | 1.545 | **PRKDC** | -1.533 |
| **TNFSF9** | 1.545 | **FIGNL1** | -1.652 | **UXT** | 1.544 | **SH3KBP1** | -1.531 |
| **HCST** | 1.542 | **ATAD2** | -1.651 | **NFE2L1** | 1.544 | **SCRIB** | -1.530 |
| **CHMP5** | 1.542 | **PRKAA1** | -1.651 | **VOPP1** | 1.542 | **AFAP1** | -1.522 |
| **MCOLN2** | 1.542 | **WEE1** | -1.646 | **MBOAT7** | 1.541 | **RUNX1** | -1.518 |
| **NUPR1** | 1.527 | **TPM3** | -1.646 | **NUP62** | 1.540 | **PCDHGA3** | -1.514 |
| **HTATIP2** | 1.525 | **CCNI** | -1.643 | **TSC22D3** | 1.539 | **CDT1** | -1.513 |
| **SAT1** | 1.522 | **JAK2** | -1.642 | **BCL2L12** | 1.536 | **RIPK1** | -1.509 |
| **BIN1** | 1.521 | **CES1** | -1.640 | **TNFAIP8** | 1.529 | **ULBP3** | -1.508 |
| **VGF** | 1.517 | **HS.128753** | -1.638 | **SLC3A2** | 1.529 | **ING3** | -1.506 |
| **CDC42** | 1.512 | **TXNDC5** | -1.637 | **HSPA1B** | 1.527 | **SELL** | -1.506 |
| **TMEM158** | 1.506 | **BCKDK** | -1.637 | **HSPE1** | 1.527 | **TRIM13** | -1.506 |
| **SNN** | 1.500 | **ZNF184** | -1.634 | **E2F7** | 1.524 | **PHF17** | -1.503 |
|  |  | **YAP1** | -1.632 | **GRINA** | 1.524 | **BACH2** | -1.501 |
|  |  | **HLTF** | -1.632 | **VEGFB** | 1.519 | **PTPN2** | -1.500 |
|  |  | **CASC5** | -1.631 | **PI4KB** | 1.517 |  |  |
|  |  | **NR2C2** | -1.630 | **BCL2A1** | 1.515 |  |  |
|  |  | **CDC45L** | -1.619 | **PHB** | 1.515 |  |  |
|  |  | **DPYD** | -1.618 | **CLN3** | 1.514 |  |  |
|  |  | **CIT** | -1.618 | **TNIP2** | 1.507 |  |  |
|  |  | **ELMO1** | -1.618 | **RCAN1** | 1.506 |  |  |
|  |  | **RAD18** | -1.618 | **DAD1** | 1.503 |  |  |
|  |  | **CTBP2** | -1.618 | **CDK5R1** | 1.502 |  |  |
|  |  | **PAK4** | -1.617 |  |  |  |  |
|  |  | **FOXA2** | -1.607 |  |  |  |  |
|  |  | **DIDO1** | -1.601 |  |  |  |  |
|  |  | **LGMN** | -1.586 |  |  |  |  |
|  |  | **PLK1** | -1.583 |  |  |  |  |
|  |  | **AKT2** | -1.579 |  |  |  |  |
|  |  | **NCAPG2** | -1.577 |  |  |  |  |
|  |  | **PDGFC** | -1.566 |  |  |  |  |
|  |  | **SERPINB9** | -1.566 |  |  |  |  |
|  |  | **CDC14A** | -1.562 |  |  |  |  |
|  |  | **EDNRB** | -1.558 |  |  |  |  |
|  |  | **CTCF** | -1.557 |  |  |  |  |
|  |  | **CASZ1** | -1.555 |  |  |  |  |
|  |  | **ASAH2** | -1.554 |  |  |  |  |
|  |  | **COL4A1** | -1.553 |  |  |  |  |
|  |  | **MERTK** | -1.553 |  |  |  |  |
|  |  | **NTS** | -1.549 |  |  |  |  |
|  |  | **FOXB1** | -1.545 |  |  |  |  |
|  |  | **BLVRA** | -1.543 |  |  |  |  |
|  |  | **SP3** | -1.542 |  |  |  |  |
|  |  | **EMX2** | -1.541 |  |  |  |  |
|  |  | **GABRR1** | -1.540 |  |  |  |  |
|  |  | **E2F8** | -1.540 |  |  |  |  |
|  |  | **EXOC2** | -1.538 |  |  |  |  |
|  |  | **EIF2C3** | -1.538 |  |  |  |  |
|  |  | **TP73L** | -1.537 |  |  |  |  |
|  |  | **CXADR** | -1.537 |  |  |  |  |
|  |  | **CREB1** | -1.536 |  |  |  |  |
|  |  | **SMN1** | -1.533 |  |  |  |  |
|  |  | **CTTN** | -1.532 |  |  |  |  |
|  |  | **MCM10** | -1.530 |  |  |  |  |
|  |  | **MIR135A2** | -1.529 |  |  |  |  |
|  |  | **MCL1** | -1.529 |  |  |  |  |
|  |  | **AKT3** | -1.527 |  |  |  |  |
|  |  | **ABCC4** | -1.524 |  |  |  |  |
|  |  | **MTBP** | -1.524 |  |  |  |  |
|  |  | **CAMKK1** | -1.524 |  |  |  |  |
|  |  | **CP** | -1.524 |  |  |  |  |
|  |  | **PDE4B** | -1.522 |  |  |  |  |
|  |  | **TCF4** | -1.521 |  |  |  |  |
|  |  | **LRP2** | -1.519 |  |  |  |  |
|  |  | **NHEJ1** | -1.519 |  |  |  |  |
|  |  | **CAST** | -1.518 |  |  |  |  |
|  |  | **BMP5** | -1.517 |  |  |  |  |
|  |  | **ABCE1** | -1.517 |  |  |  |  |
|  |  | **SGK1** | -1.516 |  |  |  |  |
|  |  | **ADRA1A** | -1.516 |  |  |  |  |
|  |  | **MEF2C** | -1.515 |  |  |  |  |
|  |  | **CLEC5A** | -1.515 |  |  |  |  |
|  |  | **FGF10** | -1.514 |  |  |  |  |
|  |  | **SIAH2** | -1.514 |  |  |  |  |
|  |  | **CD55** | -1.514 |  |  |  |  |
|  |  | **RABGGTB** | -1.511 |  |  |  |  |
|  |  | **VPS13A** | -1.511 |  |  |  |  |
|  |  | **IL1RN** | -1.510 |  |  |  |  |
|  |  | **CALB1** | -1.510 |  |  |  |  |
|  |  | **NOVA1** | -1.509 |  |  |  |  |
|  |  | **CAPRIN1** | -1.509 |  |  |  |  |
|  |  | **PKP3** | -1.509 |  |  |  |  |
|  |  | **ACSL4** | -1.508 |  |  |  |  |
|  |  | **UBE2C** | -1.508 |  |  |  |  |
|  |  | **DNAJB1** | -1.507 |  |  |  |  |
|  |  | **MIR655** | -1.507 |  |  |  |  |
|  |  | **VASP** | -1.507 |  |  |  |  |
|  |  | **PARD3** | -1.505 |  |  |  |  |
|  |  | **HS.24119** | -1.505 |  |  |  |  |
|  |  | **RAF1** | -1.504 |  |  |  |  |
|  |  | **PDZK1** | -1.503 |  |  |  |  |
|  |  | **VEGFC** | -1.503 |  |  |  |  |
|  |  | **ST6GAL1** | -1.502 |  |  |  |  |
|  |  | **NEK1** | -1.502 |  |  |  |  |
|  |  | **MOBKL2C** | -1.501 |  |  |  |  |
|  |  | **CHEK1** | -1.500 |  |  |  |  |

C.

| **Expected** | | | | **Inversely related** | | | |
| --- | --- | --- | --- | --- | --- | --- | --- |
| **decreased migration** | | | | **increased migration** | | | |
| **up-genes (19)** | **increased migration** | **down genes (65)** | fold change | **up-genes (36)** | fold change | **down genes (19)** | fold change |
| **HMOX1** | 7.399 | **VIM** | -5.703 | **SOD2** | 5.022 | **ARRDC3** | -2.72 |
| **TIMP2** | 3.59 | **EGR1** | -4.506 | **C1QBP** | 4.647 | **NRP1** | -2.48 |
| **EBI3** | 2.681 | **CD44** | -3.853 | **CX3CL1** | 4.048 | **IRS1** | -2.37 |
| **MYH9** | 2.255 | **BMI1** | -3.772 | **CCL5** | 3.871 | **SPRY2** | -2.073 |
| **CMTM8** | 2.139 | **PXN** | -3.096 | **CXCL10** | 3.57 | **FAF1** | -1.993 |
| **IFIT2** | 2.121 | **TCF12** | -2.839 | **ETS1** | 3.545 | **GJA1** | -1.821 |
| **DNAJA3** | 2.011 | **MAP3K7** | -2.693 | **DPAGT1** | 3.026 | **CAT** | -1.729 |
| **IL15** | 1.681 | **EGFR** | -2.562 | **TNF** | 3.013 | **GNAI1** | -1.705 |
| **ATF3** | 1.666 | **RAP1B** | -2.559 | **CCL2** | 2.649 | **RASA1** | -1.692 |
| **BRMS1** | 1.657 | **SLC2A1** | -2.53 | **S100A4** | 2.414 | **NDRG1** | -1.68 |
| **RAP1GAP** | 1.64 | **PRKCA** | -2.515 | **PTP4A3** | 2.247 | **TPM3** | -1.646 |
| **DPYSL2** | 1.618 | **HIF1A** | -2.482 | **SHC1** | 2.232 | **MIR1-2** | -1.58 |
| **TSC1** | 1.606 | **HMMR** | -2.446 | **MAP3K8** | 1.958 | **CHST10** | -1.578 |
| **ARHGDIB** | 1.58 | **SLC9A3R1** | -2.433 | **CCL20** | 1.95 | **SEMA3A** | -1.561 |
| **SIRPA** | 1.566 | **AURKA** | -2.368 | **STMN3** | 1.908 | **TP73L** | -1.537 |
| **TNFSF10** | 1.555 | **CYR61** | -2.284 | **ARF1** | 1.903 | **FAM188A** | -1.529 |
| **STAT1** | 1.549 | **ECT2** | -1.989 | **IGFBP6** | 1.813 | **MTBP** | -1.524 |
| **HTATIP2** | 1.525 | **CTGF** | -1.906 | **EFNB3** | 1.812 | **LAMC2** | -1.509 |
| **GRINA** | 1.524 | **PPFIA1** | -1.889 | **EPHA2** | 1.749 | **VASP** | -1.507 |
|  |  | **PTK2** | -1.858 | **GDF15** | 1.736 |  |  |
|  |  | **CCDC88A** | -1.855 | **PRMT5** | 1.722 |  |  |
|  |  | **HGF** | -1.847 | **LIMK1** | 1.7 |  |  |
|  |  | **CDCP1** | -1.816 | **CDC25B** | 1.67 |  |  |
|  |  | **ANXA2** | -1.791 | **HRASLS3** | 1.645 |  |  |
|  |  | **ITGA2** | -1.789 | **ACTN4** | 1.638 |  |  |
|  |  | **FOXQ1** | -1.783 | **MST1** | 1.638 |  |  |
|  |  | **PTGER4** | -1.781 | **SRC** | 1.634 |  |  |
|  |  | **HAS2** | -1.758 | **ESRRA** | 1.621 |  |  |
|  |  | **KPNA2** | -1.757 | **IL15RA** | 1.606 |  |  |
|  |  | **ITGA6** | -1.757 | **DYRK1B** | 1.605 |  |  |
|  |  | **PTP4A2** | -1.746 | **RHOB** | 1.579 |  |  |
|  |  | **SSH1** | -1.708 | **TNFAIP8** | 1.529 |  |  |
|  |  | **NRG1** | -1.697 | **VEGFB** | 1.519 |  |  |
|  |  | **FOXM1** | -1.688 | **VGF** | 1.517 |  |  |
|  |  | **BCAR3** | -1.666 | **PHB** | 1.515 |  |  |
|  |  | **DOCK1** | -1.656 | **CDC42** | 1.512 |  |  |
|  |  | **PAK2** | -1.654 |  |  |  |  |
|  |  | **DVL2** | -1.653 |  |  |  |  |
|  |  | **JAK2** | -1.642 |  |  |  |  |
|  |  | **PDK1** | -1.638 |  |  |  |  |
|  |  | **ROR1** | -1.638 |  |  |  |  |
|  |  | **F2RL1** | -1.631 |  |  |  |  |
|  |  | **ELMO1** | -1.618 |  |  |  |  |
|  |  | **CTBP2** | -1.618 |  |  |  |  |
|  |  | **LMO7** | -1.618 |  |  |  |  |
|  |  | **DIDO1** | -1.601 |  |  |  |  |
|  |  | **F2R** | -1.599 |  |  |  |  |
|  |  | **PTTG1** | -1.595 |  |  |  |  |
|  |  | **RANBP9** | -1.591 |  |  |  |  |
|  |  | **WWTR1** | -1.588 |  |  |  |  |
|  |  | **KRAS** | -1.573 |  |  |  |  |
|  |  | **AR** | -1.57 |  |  |  |  |
|  |  | **DAB2** | -1.559 |  |  |  |  |
|  |  | **RAP2A** | -1.559 |  |  |  |  |
|  |  | **ITGB1** | -1.558 |  |  |  |  |
|  |  | **MERTK** | -1.553 |  |  |  |  |
|  |  | **LIN28B** | -1.551 |  |  |  |  |
|  |  | **LCK** | -1.534 |  |  |  |  |
|  |  | **CTTN** | -1.532 |  |  |  |  |
|  |  | **AKT3** | -1.527 |  |  |  |  |
|  |  | **AFAP1** | -1.522 |  |  |  |  |
|  |  | **FGF10** | -1.514 |  |  |  |  |
|  |  | **ACSL4** | -1.508 |  |  |  |  |
|  |  | **VEGFC** | -1.503 |  |  |  |  |
|  |  | **ST6GAL1** | -1.502 |  |  |  |  |
